# Supplementary material for: Finite-temperature toroidal moment amenable to direct observation in an Fe10Dy10 molecular ring
Source: Nat Commun. 2026 Jul 24;17:9030. doi: 10.1038/s41467-026-75612-6 (PMC13507247; doi:10.1038/s41467-026-75612-6)
Supplement: Supplementary file 1 [file 41467_2026_75612_MOESM1_ESM.pdf]

Supplementary Information: Finite-Temperature Toroidal Moment Amenable to Direct Observation in an $Fe_{10}Dy_{10}$ Molecular Ring

Alessandro Soncini[1*], Kieran Hymas[2], Jonas Braun[3,4,5], Yannik F. Schneider[3], Simone Calvello[1], Amer Baniodeh[3], Yanhua Lan[3], Wolfgang Wernsdorfer[5,6], Marco Affronte[7], Christopher E. Anson[3], Annie K. Powell[3,4,5*]

[1*]Department of Chemical Sciences, University of Padova, Via Marzolo 1, Padova, 35123, Italy.
[2]Commonwealth Scientific and Industrial Research Organisation, CSIRO, Clayton, 3168, Victoria, Australia.
[3]Institute of Inorganic Chemistry, Karlsruhe Institute of Technology (KIT), Kaiserstr. 12, Karlsruhe, 76131, Germany.
[4]Institute of Nanotechnology, Karlsruhe Institute of Technology (KIT), Kaiserstr. 12, Karlsruhe, 76131, Germany.
[5]Institute for Quantum Materials and Technologies, Karlsruhe Institute of Technology (KIT), Kaiserstr. 12, Karlsruhe, 76131, Germany.
[6]Physikalisches Institut, Karlsruhe Institute of Technology (KIT), Kaiserstr. 12, Karlsruhe, 76131, Germany.
[7]Dipartimento di Scienze Fisiche, Informatiche e Matematiche, University of Modena and Reggio Emilia, Via Campi 213A, Modena, 41125, Italy.

*Corresponding author(s). E-mail(s): alessandro.soncini@unipd.it; annie.powell@kit.edu;

Contents

1 Supplementary Note 1: Computational Data **3**

2 Supplementary Note 2: Toroidal and Magnetic Spectrum **13**

3 Supplementary Note 3: Synthesis **14**

4 Supplementary Note 4: AC SQUID Magnetometry **15**

5 Supplementary Note 5: MicroSQUID **16**

6 Supplementary Note 6: Calculation of the Magnetoelectric Effect Tensor in $Fe_{10}Dy_{10}$ **18**

6.1 The effective single-ion electric Hamiltonian 19

6.1.1 Electric Hamiltonian in the coupled Irreducible Tensor Representation 22

6.1.2 Matrix elements of the electric Hamiltonian within the ground spin-orbit multiplet 24

6.2 Local and Global Magnetoelectric tensors 27

6.3 Magnetoelectric tensor: numerical evaluation 30

7 Supplementary Note 7: Dynamical accumulation of toroidal polarisation **37**

7.1 Effective model for the accumulation of toroidal polarisation 37

7.2 Dynamical simulations of toroidal polarisation in $Fe_{10}Dy_{10}$ 40

7.2.1 Laser heating and pulse-train thermal accumulation 43

8 χ^2 analysis of the exchange coupling parameters J_A, J_B and J_C **47**

1 Supplementary Note 1: Computational Data

As reported in the Methods section of the manuscript, subsection 3.1, we performed Configurationally Averaged Hartree Fock (CAHF)/Complete Active Space Configuration Interaction-Spin Orbit (CASCI-SO) calculations on the five symmetry unique $[Dy^{III}Fe_2^{III}\ (MeTeaH^{2-})(MeTea^{3-})_2(CH_3O^-)_2NO_3^-]^{2-}$ (extended) fragments of the $Fe_{10}Dy_{10}$ molecular wheel using the in-house developed ab initio quantum chemistry code CERES [1, 2], a methodology which allows for the full representation of the spin-orbit coupling problem in the complete basis of the 2002 states arising from full set of $4f^9$ Russell-Saunders terms. In the following we report ten tables, two for each of the five fragments, reporting the obtained energy spectrum for the eight lowest lying Kramers Doublets (KD) describing the splitting of the ion's $^6H_{15/2}$ ground spin-orbit multiplet and all the calculated $g-$tensors for each KD (Tables S1 to S5), and the Cartesian coordinates of the five fragments employed in the calculations (Tables S6 to S10).

Table S1 Energy levels (E in cm^{-1}), g-tensor principal values (g_X, g_Y, g_Z), and magnetic axes ℓ for Dy(III) centre 1. Lower-case x, y, z denote the molecular Cartesian frame; upper-case X, Y, Z denote the magnetic axes frame.

Level	E (cm^{-1})	g_X	g_Y	g_Z	ℓ_X	ℓ_Y	ℓ_Z	
1	0.0000	0.0513	0.1079	19.5543	0.6606	0.0266	-0.7503	x
					0.4621	-0.8021	0.3784	y
					0.5917	0.5966	0.5422	z
2	104.9188	1.4336	3.2376	15.1467	-0.7489	-0.4732	-0.4640	x
					-0.5313	0.8471	-0.0064	y
					-0.3961	-0.2417	0.8858	z
3	159.3273	0.0425	3.6814	13.2187	-0.8349	0.5326	-0.1385	x
					0.3661	0.7255	0.5827	y
					0.4109	0.4358	-0.8008	z
4	245.9281	2.6952	7.3032	9.0668	0.5825	-0.7105	0.3948	x
					-0.7508	-0.2841	0.5964	y
					0.3116	0.6438	0.6989	z
5	350.8657	1.6897	2.5212	12.9296	0.4253	-0.1921	-0.8844	x
					0.6376	0.7571	0.1422	y
					-0.6423	0.6244	-0.4445	z
6	430.7699	0.4442	4.1951	12.8282	0.9705	0.0212	0.2401	x
					-0.2100	0.5633	0.7991	y
					-0.1183	-0.8260	0.5512	z
7	471.1037	1.4812	3.3161	13.7486	-0.8044	-0.4058	-0.4339	x
					0.5840	-0.6740	-0.4524	y
					0.1089	0.6173	-0.7792	z
8	508.6755	0.4560	2.6369	16.7674	0.0956	-0.6436	-0.7594	x
					-0.1087	0.7516	-0.6507	y
					0.9895	0.1448	0.0019	z

Table S2 Energy levels (E in cm^{-1}), g-tensor principal values (g_X, g_Y, g_Z), and magnetic axes ℓ for Dy(III) centre 2. Lower-case x, y, z denote the molecular Cartesian frame; upper-case X, Y, Z denote the magnetic axes frame.

Level	E (cm^{-1})	g_X	g_Y	g_Z	ℓ_X	ℓ_Y	ℓ_Z	
1	0.0000	0.0739	0.1696	19.3989	-0.6751	0.7239	-0.1423	x
					0.2088	0.3725	0.9042	y
					0.7076	0.5807	-0.4026	z
2	109.9774	1.6588	4.1751	14.0786	0.6252	0.7327	-0.2688	x
					-0.0877	-0.2763	-0.9571	y
					-0.7755	0.6219	-0.1085	z
3	156.9658	1.8326	3.1368	12.6125	0.1309	-0.8073	-0.5755	x
					-0.7464	0.3018	-0.5931	y
					0.6525	0.5072	-0.5631	z
4	243.9130	1.6464	4.8045	11.0403	-0.7540	0.5337	-0.3831	x
					-0.0561	0.5287	0.8469	y
					-0.6545	-0.6600	0.3687	z
5	305.3286	2.5094	4.0827	11.8639	-0.7852	0.6186	-0.0297	x
					-0.6172	-0.7855	-0.0453	y
					-0.0513	-0.0173	0.9985	z
6	372.1516	1.0320	2.4707	16.0101	0.5362	0.6930	-0.4820	x
					-0.8427	0.4066	-0.3529	y
					-0.0486	0.5954	0.8020	z
7	434.6479	0.1362	0.5869	18.1414	0.8994	-0.3853	-0.2066	x
					-0.1727	-0.7472	0.6418	y
					0.4016	0.5415	0.7385	z
8	485.3261	0.3935	0.6378	18.5629	0.1299	0.2232	-0.9661	x
					-0.6000	0.7934	0.1026	y
					0.7894	0.5663	0.2369	z

Table S3 Energy levels (E in cm^{-1}), g-tensor principal values (g_X, g_Y, g_Z), and magnetic axes ℓ for Dy(III) centre 3. Lower-case x, y, z denote the molecular Cartesian frame; upper-case X, Y, Z denote the magnetic axes frame.

Level	E (cm^{-1})	g_X	g_Y	g_Z	ℓ_X	ℓ_Y	ℓ_Z	
1	0.0000	0.1966	0.5761	19.0879	0.0571	-0.4645	-0.8837	x
					-0.9976	0.0084	-0.0688	y
					0.0394	0.8855	-0.4629	z
2	60.2817	1.3496	2.5570	16.1305	-0.5151	0.8305	-0.2118	x
					-0.8547	-0.5162	0.0547	y
					-0.0639	0.2092	0.9758	z
3	107.5464	0.3789	2.6666	13.5211	-0.2560	0.2621	-0.9305	x
					-0.3631	0.8660	0.3438	y
					0.8959	0.4259	-0.1266	z
4	168.0204	3.0391	6.5057	10.2668	0.3005	0.8381	-0.4553	x
					0.1280	0.4376	0.8900	y
					0.9452	-0.3257	0.0242	z
5	243.0220	1.6532	2.9395	12.8906	-0.2935	-0.9139	-0.2805	x
					-0.5064	0.3975	-0.7652	y
					0.8108	-0.0826	-0.5795	z
6	316.5837	1.4895	5.2113	11.3402	-0.6522	-0.7577	0.0218	x
					0.0562	-0.0197	0.9982	y
					-0.7560	0.6523	0.0554	z
7	340.2952	1.7217	4.3676	14.1119	0.3201	0.8156	-0.4820	x
					-0.2629	0.5652	0.7819	y
					0.9102	-0.1236	0.3954	z
8	384.5561	0.4714	1.2639	17.8304	0.6031	0.7680	-0.2154	x
					-0.5301	0.1842	-0.8277	y
					-0.5960	0.6134	0.5182	z

Table S4 Energy levels (E in cm^{-1}), g-tensor principal values (g_X, g_Y, g_Z), and magnetic axes ℓ for Dy(III) centre 4. Lower-case x, y, z denote the molecular Cartesian frame; upper-case X, Y, Z denote the magnetic axes frame.

Level	E (cm^{-1})	g_X	g_Y	g_Z	ℓ_X	ℓ_Y	ℓ_Z	
1	0.0000	0.2246	0.6788	18.8523	-0.9917	0.0037	-0.1286	x
					0.0537	0.9202	-0.3877	y
					-0.1169	0.3914	0.9128	z
2	64.2084	1.4697	2.3150	15.6634	0.8999	-0.4351	-0.0293	x
					0.0984	0.1373	0.9856	y
					-0.4249	-0.8898	0.1664	z
3	112.0278	0.8123	2.5048	13.4126	0.0877	0.9587	-0.2705	x
					0.5393	0.1826	0.8221	y
					0.8376	-0.2179	-0.5010	z
4	172.0512	1.8078	4.3641	11.6547	-0.0809	0.4853	-0.8706	x
					0.5274	0.7620	0.3758	y
					0.8458	-0.4288	-0.3176	z
5	241.7640	2.4895	4.4367	11.1259	-0.4469	0.6840	-0.5766	x
					-0.8910	-0.2824	0.3556	y
					0.0804	0.6726	0.7356	z
6	304.2819	0.9727	2.5583	15.3999	0.0498	0.6670	-0.7434	x
					0.1691	0.7279	0.6645	y
					0.9843	-0.1588	-0.0766	z
7	333.3431	1.7829	4.6767	13.5146	-0.3294	-0.1034	-0.9385	x
					-0.2612	0.9652	-0.0146	y
					-0.9073	-0.2403	0.3449	z
8	423.7091	0.1932	0.5165	18.9094	-0.4561	-0.3261	-0.8280	x
					0.8857	-0.0752	-0.4582	y
					-0.0872	0.9423	-0.3231	z

Table S5 Energy levels (E in cm^{-1}), g-tensor principal values (g_X, g_Y, g_Z), and magnetic axes ℓ for Dy(III) centre 5. Lower-case x, y, z denote the molecular Cartesian frame; upper-case X, Y, Z denote the magnetic axes frame.

Level	E (cm^{-1})	g_X	g_Y	g_Z	ℓ_X	ℓ_Y	ℓ_Z	
1	0.0000	0.0006	0.0045	19.6428	0.7739	-0.3155	0.5492	x
					-0.6311	-0.3110	0.7106	y
					0.0533	0.8965	0.4398	z
2	147.4923	0.1981	0.2648	16.7278	0.1428	-0.6423	-0.7530	x
					-0.7561	0.4201	-0.5018	y
					0.6386	0.6411	-0.4256	z
3	270.8987	1.6195	2.0257	15.6096	-0.2420	-0.1813	-0.9532	x
					-0.9366	-0.2128	0.2783	y
					0.2533	-0.9601	0.1183	z
4	333.4562	1.5166	5.8988	8.7713	-0.3239	-0.3564	-0.8764	x
					0.6952	-0.7179	0.0350	y
					0.6417	0.5980	-0.4803	z
5	445.5660	1.3922	4.1935	8.1047	0.8930	-0.2408	-0.3801	x
					0.3013	-0.3075	0.9026	y
					-0.3342	-0.9206	-0.2021	z
6	499.8369	0.3695	3.3657	14.2427	0.2309	0.6899	-0.6861	x
					0.9528	-0.0173	0.3032	y
					0.1973	-0.7237	-0.6613	z
7	553.1442	0.9772	4.1750	9.7386	0.0210	0.9807	-0.1943	x
					0.9707	0.0266	0.2389	y
					0.2395	-0.1936	-0.9514	z
8	607.4521	1.2112	4.9364	15.0897	-0.9066	-0.2661	-0.3275	x
					0.0384	0.7208	-0.6920	y
					-0.4202	0.6400	0.6433	z

Table S6 Cartesian coordinates (in Å) of the Dy(III) molecular fragment for centre 1.

Atom	x	y	z
Dy	0.000000	0.000000	0.000000
Ga	0.301026	-2.222745	-2.552862
N	-2.173398	1.057926	-0.919286
C	-3.150469	1.209321	0.185114
H	-3.678240	2.032909	0.034869
H	-3.777081	0.442405	0.163650
C	-2.538500	1.277968	1.515857
H	-2.106430	2.159589	1.640737
H	-3.236016	1.174499	2.210498
O	-1.579224	0.248709	1.644639
C	-2.695564	0.178901	-1.969052
H	-2.260380	0.409396	-2.827596
H	-3.668044	0.340037	-2.070516
C	-2.471166	-1.249766	-1.682220
H	-3.039463	-1.530002	-0.921237
H	-2.723372	-1.791244	-2.470519
O	-1.105008	-1.468322	-1.368071
C	-1.754425	2.328632	-1.502706
H	-1.570894	2.958402	-0.761236
H	-2.518056	2.694474	-2.013930
C	-0.587116	2.325701	-2.384665
H	-0.919608	2.130334	-3.307600
C	0.146904	3.629024	-2.451007
H	0.890680	3.554514	-3.087110
H	0.496463	3.853499	-1.563194
H	-0.465038	4.336012	-2.749546
O	0.326261	1.260465	-2.029540
H	0.606099	0.526858	-2.614911
N	2.371912	-2.672709	-3.274429
C	3.332295	-1.977038	-2.429543
H	4.012872	-1.549626	-3.009061
H	3.797336	-2.643476	-1.863685
C	2.737781	-0.942305	-1.555389
H	3.331065	-0.776097	-0.780748
H	2.641124	-0.095344	-2.058808
O	1.435462	-1.380607	-1.085141
C	2.510737	-4.139531	-3.200282
H	3.469279	-4.386830	-3.169062
H	2.109484	-4.557360	-4.004192
C	1.814235	-4.635098	-1.967100
H	1.823501	-5.625123	-1.945637
H	2.273627	-4.298716	-1.157337
O	0.470463	-4.154447	-2.008076
C	2.342430	-2.132596	-4.646148
H	1.829061	-2.744194	-5.231519
H	3.266245	-2.072621	-4.999322
C	1.690869	-0.727844	-4.661758
H	2.370473	-0.070069	-4.341756
C	1.365636	-0.400928	-6.051039
H	0.944140	0.482961	-6.088112
H	0.749197	-1.072905	-6.410067
H	2.187618	-0.392555	-6.585678
O	0.589777	-0.678143	-3.779799
C	-1.615513	-2.507853	-4.921273
H	-1.005175	-2.472638	-5.701768
H	-1.887253	-1.583307	-4.696881
O	-0.948580	-3.102858	-3.811019
N	1.665431	2.154570	0.948047
O	0.420581	2.321552	0.786095
O	2.128173	1.018785	0.672923
O	2.393435	3.033686	1.396832
Ga	-0.990192	-0.921511	3.161335
C	-0.114328	1.812893	4.079783
H	0.761124	2.264319	3.968562
H	-0.673641	2.035296	3.293434
O	0.080062	0.421630	4.136368
N	-1.981217	-2.850136	2.750990
C	-1.655409	-3.773853	3.859293
H	-2.385175	-3.743293	4.526616
H	-1.611159	-4.697377	3.504168
C	-0.305357	-3.442474	4.571494
H	0.447030	-3.851714	4.077831
H	-0.311514	-3.809673	5.492478
O	-0.156050	-2.086165	4.610519
C	-1.413453	-3.331648	1.498296
H	-1.507463	-4.317148	1.457320
H	-1.922860	-2.947811	0.743168
C	0.033537	-2.971089	1.351954
H	0.333707	-3.148762	0.425116
H	0.583275	-3.515763	1.968544
O	0.185467	-1.563455	1.662200
C	-3.416867	-2.469872	2.750990
H	-3.630736	-1.984902	1.913910
H	-3.976848	-3.285943	2.786113
C	-3.729249	-1.587303	3.941245
H	-3.772899	-2.180122	4.745154
C	-5.050391	-0.921433	3.816366
H	-5.205350	-0.351380	4.598812
H	-5.064663	-0.372531	3.004651
H	-5.754406	-1.601695	3.763682
O	-2.687519	-0.668037	4.157832
H	-2.477442	-3.096585	-5.156588
H	-0.589403	2.171957	4.968758

Table S7 Cartesian coordinates (in Å) of the Dy(III) molecular fragment for centre 2.

Atom	x	y	z
Dy	0.000000	0.000000	0.000000
Ga	1.501110	3.049960	0.601180
Ga	1.920530	-2.469210	-1.347720
C	-1.271080	4.022940	1.471820
O	0.095080	3.804380	1.785970
N	3.572000	2.600000	-0.120390
C	4.532380	3.295670	0.724490
H	5.212960	3.723080	0.144980
H	4.997420	2.629230	1.290350
C	3.937870	4.330400	1.598650
H	4.531150	4.496610	2.373290
H	3.841210	5.177360	1.095230
O	2.635550	3.892100	2.068900
C	3.710820	1.133170	-0.046240
H	4.669370	0.885880	-0.015020
H	3.309570	0.715350	-0.850150
C	3.014320	0.637610	1.186940
H	3.023590	-0.352420	1.208400
H	3.473710	0.973990	1.996700
O	1.670550	1.118260	1.145960
C	3.542520	3.140110	-1.492110
H	3.029150	2.528510	-2.077480
H	4.466330	3.200080	-1.845280
C	2.890960	4.544860	-1.507720
H	3.570560	5.202640	-1.187720
C	2.565720	4.871780	-2.897000
H	2.144230	5.755670	-2.934070
H	1.949280	4.199800	-3.256030
H	3.387710	4.880150	-3.431640
O	1.789870	4.594560	-0.625760
N	-1.253120	0.516630	-2.147730
C	-1.623290	1.939830	-2.096990
H	-2.322380	2.079170	-1.410160
H	-1.991690	2.219450	-2.973100
C	-0.415420	2.764850	-1.767240
H	0.194910	2.800070	-2.547730
H	-0.687170	3.689400	-1.542840
O	0.251510	2.169850	-0.656980
C	-0.430100	0.128350	-3.271640
H	-0.546380	0.807620	-3.983840
H	-0.777980	-0.726750	-3.624810
C	1.025230	-0.031990	-3.027730
H	1.416410	-0.588270	-3.747740
H	1.461330	0.856470	-3.055050
O	1.282290	-0.638290	-1.780890
C	-2.531200	-0.250530	-2.118460
H	-3.169590	0.231640	-1.535040
H	-2.912020	-0.254250	-3.031640
C	-2.446530	-1.549080	-1.681380
H	-1.983900	-1.936510	-2.479440
C	-3.553710	-2.506310	-1.599430
H	-3.212400	-3.377190	-1.308690
H	-3.974280	-2.597400	-2.479440
H	-4.217420	-2.183220	-0.953570
O	-1.439320	-1.788700	-0.731130
H	-0.858450	-2.326080	-1.185770
N	2.255880	-4.315390	-0.149660
C	1.709250	-4.115170	1.177180
H	2.460360	-4.021040	1.815240
H	1.203440	-4.926440	1.432790
C	0.835140	-2.964840	1.323520
H	-0.092020	-3.226360	1.093280
H	0.842130	-2.666800	2.265970
O	1.242530	-1.870330	0.476690
C	3.723890	-4.518630	-0.155510
H	4.008410	-4.930120	-1.010160
H	3.990340	-5.119130	0.585960
C	4.376270	-3.148400	0.012290
H	4.157260	-2.781690	0.905960
H	5.360700	-3.233240	-0.056000
O	3.895560	-2.278650	-0.998450
C	1.515110	-5.348220	-0.867720
H	1.424570	-6.153040	-0.296000
H	2.009150	-5.606510	-1.685280
C	0.113900	-4.823360	-1.252110
H	-0.464450	-4.836850	-0.436490
C	-0.553700	-5.667180	-2.337000
H	-1.427630	-5.281550	-2.557490
H	-0.674920	-6.584150	-2.009190
H	0.011880	-5.677700	-3.137000
O	0.242610	-3.477330	-1.691140
C	1.968100	-3.303750	-4.301890
O	2.659230	-2.846040	-3.156520
N	-2.026360	0.162700	2.029870
O	-0.956570	-0.534560	2.240610
O	-2.134770	0.797660	0.921570
O	-2.903290	0.194920	2.904030
H	-1.769850	4.441160	2.321060
H	-1.730360	3.092390	1.210960
H	-1.343370	4.700090	0.646510
H	2.655130	-3.406260	-5.115760
H	1.206820	-2.599150	-4.564370
H	1.519320	-4.252210	-4.092350

Table S8 Cartesian coordinates (in Å) of the Dy(III) molecular fragment for centre 3.

Atom	x	y	z
Dy	0.000000	0.000000	0.000000
Ga	-2.965000	-0.203960	1.743080
Ga	2.341720	-2.407070	0.552240
C	-3.860300	2.233250	0.063060
H	-3.469130	1.676970	-0.656940
H	-3.424200	3.121710	0.035750
O	-3.603240	1.626950	1.309910
N	-2.629660	-2.050150	2.941140
C	-3.176290	-1.849930	4.267980
H	-2.425180	-1.755800	4.906030
H	-3.682090	-2.661200	4.523590
C	-4.050390	-0.699600	4.414320
H	-4.977560	-0.961120	4.184080
H	-4.043410	-0.401560	5.356770
O	-3.643000	0.394920	3.567490
C	-1.161650	-2.253390	2.935290
H	-0.877130	-2.664870	2.080640
H	-0.895190	-2.853890	3.676750
C	-0.509260	-0.883160	3.103090
H	-0.728270	-0.516440	3.996760
H	0.475170	-0.968000	3.034800
O	-0.989970	-0.013410	2.092350
C	-3.370420	-3.082980	2.223080
H	-3.460970	-3.887800	2.794800
H	-2.876380	-3.341270	1.405510
C	-4.771640	-2.558120	1.838690
H	-5.349980	-2.571600	2.654310
C	-5.439230	-3.401930	0.753800
H	-6.313170	-3.016300	0.533310
H	-5.560460	-4.318910	1.081610
H	-4.873660	-3.412460	-0.046210
O	-4.642920	-1.212090	1.399660
N	-0.688440	-0.901140	-2.315490
C	-2.177530	-0.628350	-2.444280
H	-2.322190	0.337800	-2.606230
H	-2.535910	-1.124590	-3.222820
C	-2.917430	-1.038500	-1.211090
H	-3.829650	-0.655280	-1.224750
H	-2.995570	-2.025280	-1.181820
O	-2.226300	-0.580800	-0.065720
C	-0.385060	-2.360340	-2.362320
H	0.472210	-2.493340	-2.838420
H	-1.092560	-2.815820	-2.885250
C	-0.291670	-3.005160	-1.010120
H	-1.200320	-3.116400	-0.633530
H	0.117700	-3.902370	-1.097920
O	0.490010	-2.209670	-0.137910

Atom	x	y	z
C	0.014440	-0.188650	-3.363310
H	-0.353780	0.728410	-3.435500
H	-0.136670	-0.647410	-4.227710
C	1.514730	-0.108860	-3.092090
H	1.916470	-0.999160	-3.298920
C	2.229670	0.968100	-3.979900
H	3.185500	0.986530	-3.763310
H	1.837460	1.848160	-3.806240
H	2.113870	0.738590	-4.926250
O	1.727100	0.165350	-1.734020
H	2.399550	-0.363210	-1.295000
N	4.289030	-1.772850	1.186980
C	4.157000	-0.347400	1.709910
H	4.019000	-0.374750	2.691380
H	4.998740	0.141090	1.536250
C	3.024500	0.382640	1.075760
H	2.833710	1.223800	1.561610
H	3.232860	0.597760	0.131360
O	1.902600	-0.524470	1.159660
C	4.509100	-2.664630	2.402600
H	4.892930	-3.529750	2.115770
H	5.150250	-2.231910	3.021140
C	3.207980	-2.901920	3.112850
H	3.371020	-3.528960	3.860170
H	2.905800	-2.044110	3.503100
O	2.130330	-3.437870	2.281620
C	5.179730	-1.921900	-0.046210
H	6.074870	-1.554930	0.162580
H	5.287760	-2.884190	-0.245230
C	4.652720	-1.234080	-1.265730
H	4.745150	-0.240050	-1.209140
C	5.203280	-1.806610	-2.619890
H	4.837770	-1.287350	-3.367210
H	4.934680	-2.745090	-2.711590
H	6.181970	-1.744790	-2.627690
O	3.362260	-1.659630	-0.974990
C	2.815760	-4.667530	-1.402310
H	3.789780	-4.671750	-1.579880
H	2.389390	-4.036520	-2.032560
O	2.579210	-4.250940	-0.083280
N	0.149640	2.811180	-0.555480
O	0.823840	2.312120	0.347950
O	-0.731620	2.056910	-1.123290
O	0.274330	3.967950	-0.949630
H	2.410850	-5.649420	-1.532070
H	-4.917270	2.331190	-0.071590

Table S9 Cartesian coordinates (in Å) of the Dy(III) molecular fragment for centre 4.

Atom	x	y	z
Dy	0.000000	0.000000	0.000000
Ga	0.328040	3.254530	-1.095290
Ga	2.441630	-1.416090	1.893030
C	-2.305350	2.656440	-2.657640
H	-3.214000	2.545200	-2.281050
H	-1.895980	1.759230	-2.745450
O	-1.523670	3.451930	-1.785440
N	2.275350	3.888750	-0.460550
C	2.143320	5.314210	0.062380
H	2.005320	5.286860	1.043850
H	2.985060	5.802690	-0.111280
C	1.010820	6.044250	-0.571770
H	0.820030	6.885410	-0.085910
H	1.219180	6.259360	-1.516170
O	-0.111080	5.137140	-0.487870
C	2.495420	2.996970	0.755070
H	2.879250	2.131860	0.468240
H	3.136570	3.429700	1.373610
C	1.194300	2.759690	1.465320
H	1.357340	2.132650	2.212640
H	0.892120	3.617500	1.855570
O	0.116650	2.223730	0.634090
C	3.166050	3.739700	-1.693730
H	4.061190	4.106680	-1.484950
H	3.274080	2.777410	-1.892760
C	2.639040	4.427520	-2.913260
H	2.731470	5.421550	-2.856670
C	3.189600	3.854990	-4.267410
H	2.824090	4.374250	-5.014740
H	2.921000	2.916510	-4.359120
H	4.168290	3.916820	-4.275220
O	1.348580	4.001980	-2.622520
N	0.614630	-1.250110	-2.162030
C	0.243310	-0.391570	-3.264480
H	-0.743480	-0.345560	-3.334720
H	0.596480	-0.765240	-4.111310
C	0.802080	0.994080	-3.049840
H	1.776100	0.989850	-3.227400
H	0.375710	1.625080	-3.680090
O	0.565530	1.410670	-1.730810
C	2.084980	-1.552240	-2.097640
H	2.198670	-2.510110	-1.869340
H	2.472730	-1.412510	-2.997160
C	2.850600	-0.754990	-1.135680
H	3.732360	-1.176560	-0.989340
H	3.002850	0.149920	-1.508370
O	2.183500	-0.641120	0.093600
C	-0.239520	-2.441860	-2.267400
H	0.179280	-3.045970	-2.928870
H	-1.105620	-2.150240	-2.647890
C	-0.537120	-3.248830	-1.055680
H	0.192360	-3.928370	-0.999090
C	-1.823270	-4.035410	-1.151290
H	-1.944840	-4.562050	-0.333720
H	-1.783980	-4.635470	-1.923980
H	-2.578310	-3.417350	-1.256660
O	-0.381940	-2.390270	0.150190
H	0.045250	-2.629400	0.998970
N	1.977590	-1.904720	4.011680
C	0.652730	-1.302910	4.390220
H	0.135280	-1.959870	4.919010
H	0.806640	-0.508700	4.959990
C	-0.125000	-0.910690	3.211680
H	-0.642640	-1.691900	2.895580
H	-0.772170	-0.209870	3.475090
O	0.666040	-0.419220	2.128740
C	2.990270	-1.276830	4.819500
H	3.785890	-1.864050	4.868280
H	2.645760	-1.145110	5.738530
C	3.406060	0.121800	4.208760
H	2.666620	0.773170	4.284860
H	4.203550	0.484200	4.669250
O	3.697680	-0.173530	2.819480
C	1.968570	-3.454860	3.896560
H	2.900080	-3.778120	3.818510
H	1.582350	-3.836620	4.723890
C	1.209720	-3.929050	2.762890
H	0.232460	-3.847980	2.963870
C	1.566110	-5.394040	2.587280
H	1.083760	-5.756170	1.814590
H	1.310440	-5.889960	3.393140
H	2.530090	-5.478810	2.440940
O	1.509600	-3.147780	1.660440
C	4.525050	-3.360780	0.663360
H	4.647730	-3.103570	-0.284940
H	3.767430	-3.996080	0.710190
O	4.212480	-2.208720	1.422390
N	-2.850930	-0.227890	0.070190
O	-2.196810	-0.014110	1.098490
O	-2.194730	-0.183460	-1.030310
O	-4.044780	-0.413970	0.089700
H	5.416980	-3.807900	1.049940
H	-2.355430	3.136800	-3.612440

Table S10 Cartesian coordinates (in Å) of the Dy(III) molecular fragment for centre 5.

Atom	x	y	z
Dy	0.000000	0.000000	0.000000
Ga	-3.373310	-0.640580	-0.244160
Ga	1.640480	0.298840	2.950990
C	-2.964340	0.020510	-3.272870
H	-2.082580	-0.401050	-3.126530
H	-2.812090	0.925420	-3.645550
O	-3.631440	0.134390	-2.043590
N	-3.837350	-1.129220	1.874490
C	-5.162210	-0.527410	2.253030
H	-5.679660	-1.184360	2.781820
H	-5.008300	0.266810	2.822800
C	-5.939940	-0.135190	1.074490
H	-6.457580	-0.916400	0.758390
H	-6.587110	0.565640	1.337900
O	-5.148900	0.356290	-0.008450
C	-2.824670	-0.501330	2.682310
H	-2.029050	-1.088540	2.731090
H	-3.169190	-0.369610	3.601340
C	-2.408880	0.897310	2.071570
H	-3.148320	1.548670	2.147670
H	-1.611390	1.259700	2.532060
O	-2.117260	0.601970	0.682290
C	-3.846380	-2.679360	1.759370
H	-2.914860	-3.002620	1.681320
H	-4.232590	-3.061120	2.586700
C	-4.605220	-3.153540	0.625700
H	-5.582480	-3.072470	0.826680
C	-4.248830	-4.618530	0.450090
H	-4.731180	-4.980670	-0.322600
H	-4.504500	-5.114460	1.255950
H	-3.284850	-4.703310	0.303750
O	-4.305340	-2.372280	-0.476750
N	0.920710	-2.413480	-0.447480
C	-0.103940	-3.225610	-1.001630
H	-0.370920	-3.886150	-0.312840
H	0.285240	-3.732440	-1.756760
C	-1.289890	-2.585270	-1.473830
H	-1.167220	-2.328070	-2.422130
H	-2.047510	-3.220580	-1.427000
O	-1.602460	-1.433220	-0.714800
C	1.414250	-2.926560	0.816920
H	2.379860	-2.714820	0.877410
H	1.330680	-3.913200	0.801310
C	0.764610	-2.435560	2.032550
H	-0.110840	-2.886990	2.143770
H	1.323920	-2.657970	2.818890
O	0.570220	-1.044300	1.975960
C	2.016800	-2.310460	-1.341140
H	1.653850	-2.255100	-2.260180
H	2.534590	-3.151360	-1.282610
C	2.953640	-1.195940	-1.163580
H	3.604140	-1.578680	-0.506010
C	3.829360	-0.790590	-2.244570
H	4.404820	-0.056540	-1.944080
H	3.290160	-0.491340	-3.007500
H	4.385900	-1.550890	-2.517740
O	2.402740	-0.097150	-0.420160
H	2.806940	0.111230	0.383750
N	2.631500	2.227470	3.361340
C	2.305690	3.151180	2.253030
H	3.035460	3.120620	1.585710
H	2.261440	4.074710	2.608160
C	0.955640	2.819810	1.540830
H	0.203250	3.229050	2.034500
H	0.961800	3.187000	0.619850
O	0.806330	1.463500	1.501810
C	2.063740	2.708980	4.614030
H	2.157750	3.694480	4.655010
H	2.573140	2.325140	5.369160
C	0.616750	2.348420	4.760370
H	0.316580	2.526090	5.687210
H	0.067010	2.893090	4.143780
O	0.464820	0.940790	4.450130
C	4.067150	1.847200	3.361340
H	4.281020	1.362230	4.198420
H	4.627130	2.663270	3.326220
C	4.379530	0.964630	2.171080
H	4.423180	1.557450	1.367170
C	5.700670	0.298760	2.295960
H	5.855630	-0.271290	1.513520
H	5.714950	-0.250140	3.107680
H	6.404690	0.979030	2.348650
O	3.337800	0.045370	1.954500
N	0.028230	1.591480	-2.402620
O	0.212670	0.333780	-2.422130
O	-0.122870	2.117430	-1.282610
O	-0.016110	2.250890	-3.438720
C	3.188780	-1.900640	4.596470
H	2.756710	-2.782260	4.471590
H	3.886300	-1.797170	3.901830
O	2.229510	-0.871380	4.467690
H	-3.555070	-0.567790	-3.943560
H	3.624710	-1.839350	5.571720

2 Supplementary Note 2: Toroidal and Magnetic Spectrum

To highlight the fact that most states are endowed with both toroidal and magnetic moment properties, we report in Figure S1 a graphical table representing the toroidal moments and magnetic moments for the six lowest Kramers Doublets as calculated via our ab initio parameterised model.

	E (cm^{-1})	$\tau/a0\mu_B$	$\lvert\tau\rvert/a0\mu_B$	μ/μ_B	$\lvert\mu\rvert/\mu_B$	Figure
1	−71.6703	$\begin{pmatrix} 88.7733 \\ -51.6358 \\ -1361.2 \end{pmatrix}$	1365.07	$\begin{pmatrix} 0 \\ 0 \\ 0 \end{pmatrix}$	0	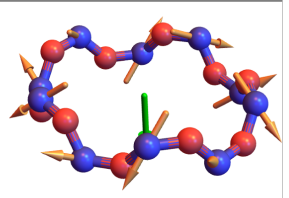
2	−71.6703	$\begin{pmatrix} -88.7733 \\ 51.6358 \\ 1361.2 \end{pmatrix}$	1365.07	$\begin{pmatrix} 0 \\ 0 \\ 0 \end{pmatrix}$	0	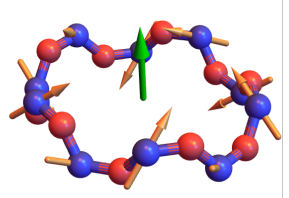
3	−71.4111	$\begin{pmatrix} 0 \\ 0 \\ 0 \end{pmatrix}$	0	$\begin{pmatrix} 77.5757 \\ 6.59177 \\ 29.6229 \end{pmatrix}$	83.3004	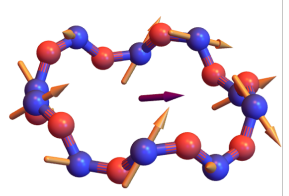
4	−71.4111	$\begin{pmatrix} 0 \\ 0 \\ 0 \end{pmatrix}$	0	$\begin{pmatrix} -77.5757 \\ -6.59177 \\ -29.6229 \end{pmatrix}$	83.3004	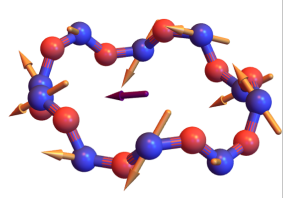
5	−71.1603	$\begin{pmatrix} 334.488 \\ 157.238 \\ -242.502 \end{pmatrix}$	442.056	$\begin{pmatrix} 70.5043 \\ -12.5991 \\ 7.20025 \end{pmatrix}$	71.9822	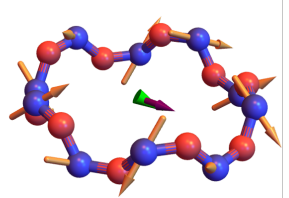
6	−71.1603	$\begin{pmatrix} -334.488 \\ -157.238 \\ 242.502 \end{pmatrix}$	442.056	$\begin{pmatrix} -70.5043 \\ 12.5991 \\ -7.20025 \end{pmatrix}$	71.9822	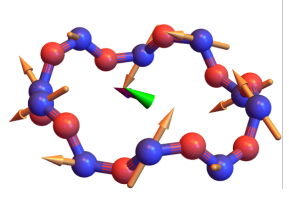
7	−71.1603	$\begin{pmatrix} -334.488 \\ -157.238 \\ 242.502 \end{pmatrix}$	442.056	$\begin{pmatrix} 70.5043 \\ -12.5991 \\ 7.20025 \end{pmatrix}$	71.9822	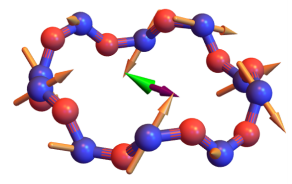
8	−71.1603	$\begin{pmatrix} 334.488 \\ 157.238 \\ -242.502 \end{pmatrix}$	442.056	$\begin{pmatrix} -70.5043 \\ 12.5991 \\ -7.20025 \end{pmatrix}$	71.9822	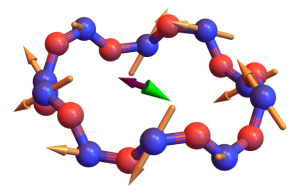
9	−70.9095	$\begin{pmatrix} 0 \\ 0 \\ 0 \end{pmatrix}$	0	$\begin{pmatrix} -63.4328 \\ 31.7899 \\ 15.2224 \end{pmatrix}$	72.5675	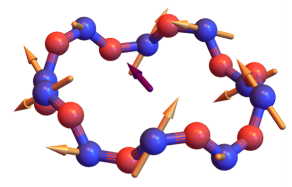
10	−70.9095	$\begin{pmatrix} 0 \\ 0 \\ 0 \end{pmatrix}$	0	$\begin{pmatrix} 63.4328 \\ -31.7899 \\ -15.2224 \end{pmatrix}$	72.5675	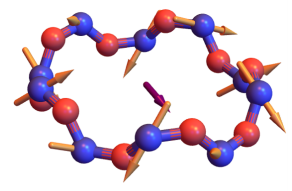
11	−70.8716	$\begin{pmatrix} -37.1652 \\ 275.709 \\ 867.921 \end{pmatrix}$	911.418	$\begin{pmatrix} 27.32 \\ 41.1455 \\ 5.68247 \end{pmatrix}$	49.7154	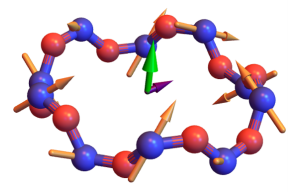
12	−70.8716	$\begin{pmatrix} 37.1652 \\ -275.709 \\ -867.921 \end{pmatrix}$	911.418	$\begin{pmatrix} -27.32 \\ -41.1455 \\ -5.68247 \end{pmatrix}$	49.7154	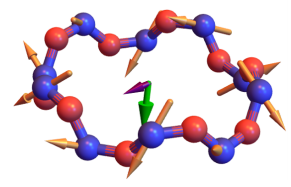

Fig. S1 The six lowest energy Kramers doublets of $Fe_{10}Dy_{10}$ computed using our parameter free theoretical model. We report the energy E, three-dimensional toroidal moment $\boldsymbol{\tau}$, its magnitude $|\boldsymbol{\tau}|$, the three-dimensional magnetic moment $\boldsymbol{\mu}$ and its magnitude $|\boldsymbol{\mu}|$ for each state. The Dy^{III} magnetic moment orientations for each state are shown as orange arrows at the site of each Dy^{III} atom (blue spheres) with the toroidal moment and magnetic moment depicted as green and purple arrows, respectively. Note that most states support both toroidal and magnetic moments simultaneously, however the largest toroidal and magnetic moments appear in the ground and first excited states, respectively, as pure quantities.

3 Supplementary Note 3: Synthesis

$[Fe_{10}Dy_{10}(Me\text{-}tea)_{10}(Me\text{-}teaH)_{10}(NO_3)_{10}]$ (see Figure S2) can be synthesised after a procedure some of us published previously [3], and the crystal structure was deposited at the Cambridge Crystallographic Data Centre (CCDC) with deposition number 885249. However, we found that using the following adapted procedure results in higher yields and better crystallinity of the product.

$Fe(NO_3)_3 \cdot 9H_2O$ (0.25 mmol) and $Dy(NO_3)_3 \cdot 6H_2O$ (0.25 mmol) were dissolved in 5 ml MeOH. A solution of Me-teaH_3 (1 mmol) and Et_3N (0.7 mmol) in 10 ml MeCN was added dropwise under stirring. After 10 minutes additional 80 μl of Et_3N (0.6 mmol) were added and the solution stirred for another 10 minutes. This resulted in a clear light brown solution which was left undisturbed for crystallisation. After two weeks the product was isolated as colourless block shaped crystals in a yield of 77% (based on Dy).

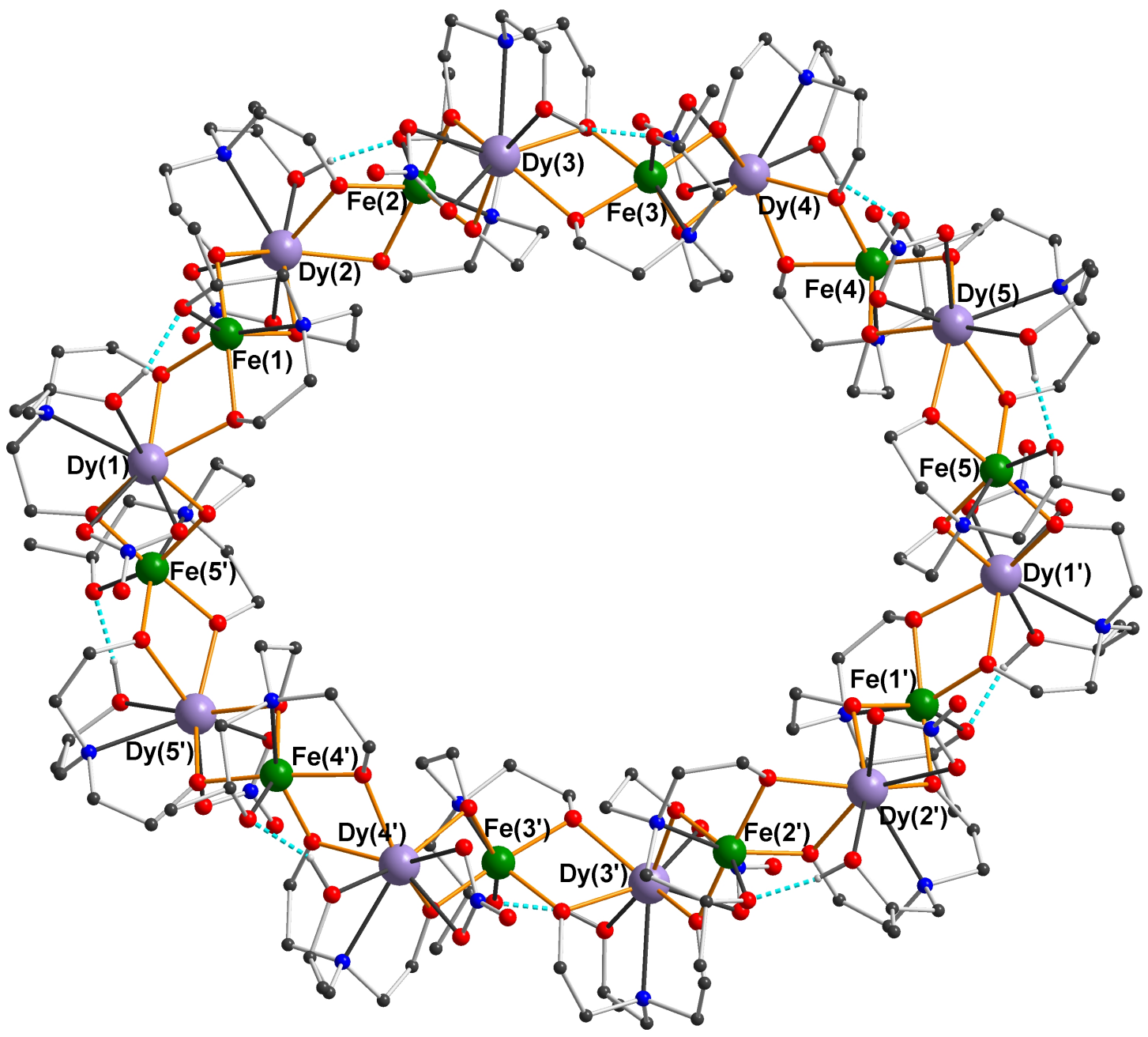

Fig. S2 Molecular structure of $Fe_{10}Dy_{10}$.

4 Supplementary Note 4: AC SQUID Magnetometry

A randomly oriented powder sample of $Fe_{10}Dy_{10}$ was immobilised in eicosane and the sample tested for slow relaxation of magnetisation using AC susceptometry. The compound shows slow relaxation with maxima in the out-of-phase component of the susceptibility below 1.8 K and above 1500 Hz and thus outside of our measurement window (see Figure S3).

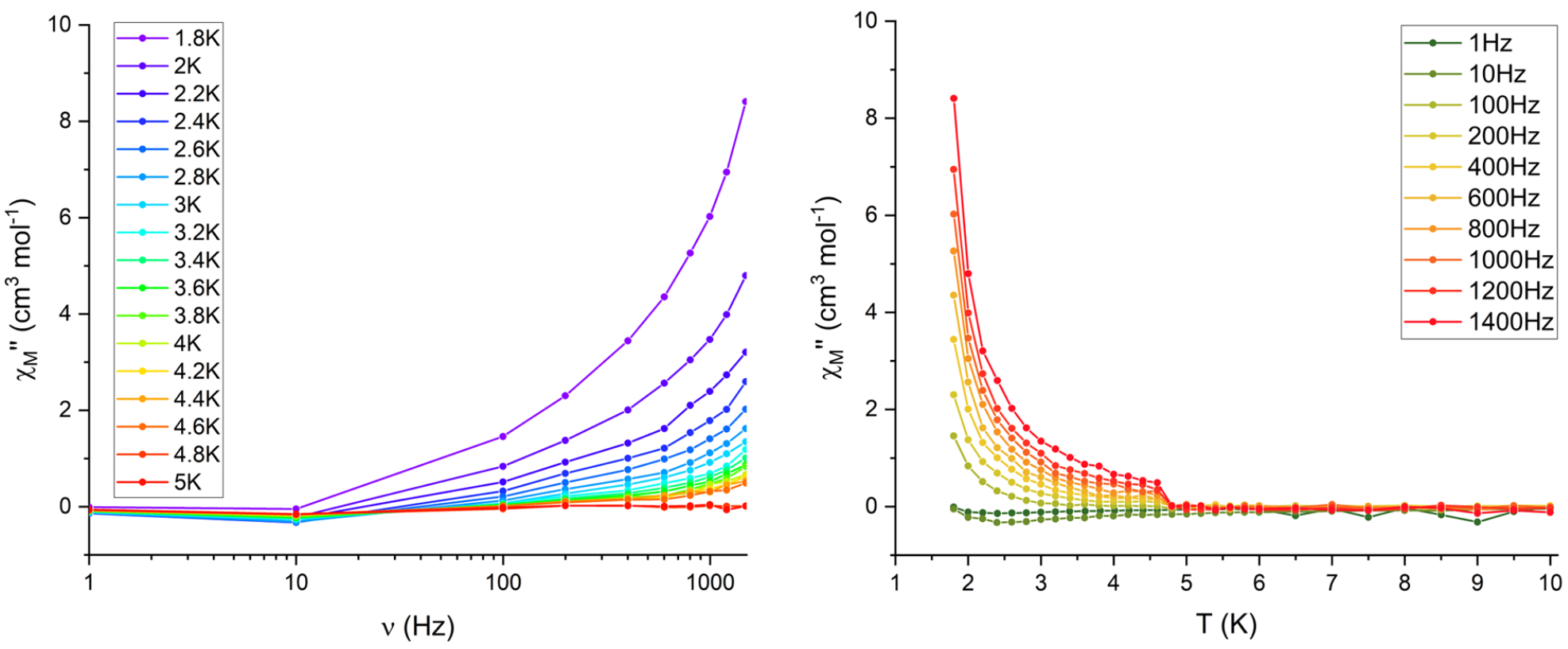

Fig. S3 Frequency dependence (left) and temperature dependence (right) of the out-of-phase component of the susceptibility indicating slow relaxation of magnetisation at low temperatures. Source data are provided as a Source Data file.

5 Supplementary Note 5: MicroSQUID

In order to assess the magnetic behavior below 1.8 K microSQUID measurements were conducted on a single crystal of $Fe_{10}Dy_{10}$ (see Figure S4). While these measurements

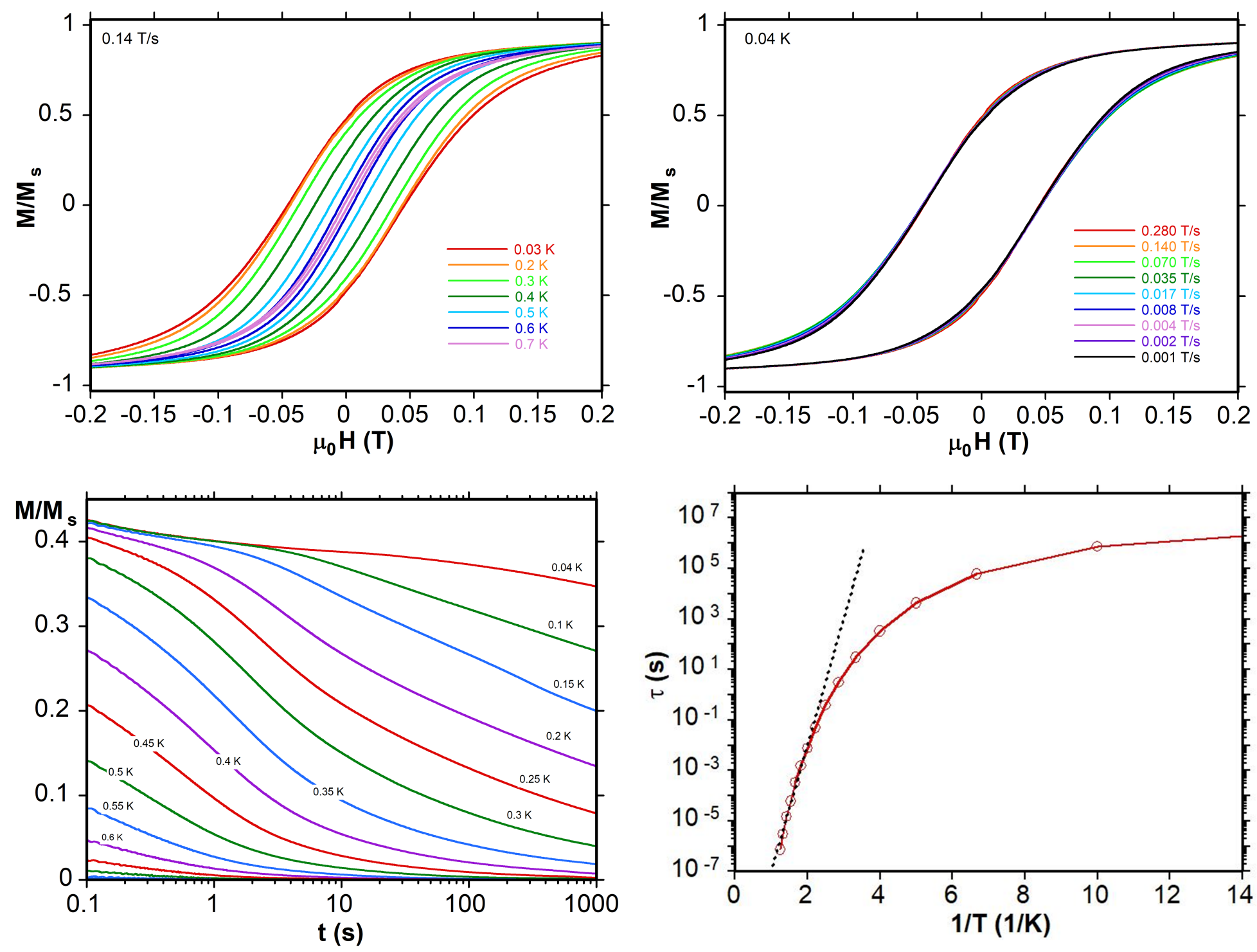

Fig. S4 Single crystal microSQUID data. Top left: Temperature dependence between 0.03 and 0.7 K at a field sweep rate of 0.14 T/s. Top right: Sweep rate dependence at 0.04 K. Bottom left: Magnetisation decay between 0.04 and 0.6 K. Bottom right: Relaxation times extracted from magnetisation decay plotted against 1/T. The linear fit results in an energy barrier $U = 11.5$ K and $\tau_0 = 8 \cdot 10^{-13}$ s. Source data are provided as a Source Data file.

are not directly relevant to assess the toroidal character of the low-energy states, the measured magnetic hysteresis with a cohercive field of ∼50mT observed here in Figure S4 is consistent with what was obtained in our single-crystal simulations of the low-temperature static equilibrium magnetisation reported in Figure 5a of the main text. In our simulations in fact, the level crossings between the non-magnetic zero-temperature ground state (i.e. the maximally toroidal s-wave Ising vortex state), and a magnetic excitation, occurs for fields between 6mT and 40mT, according to the specific orientation of the external magnetic field. The fact that the cohercive field observed in the experiment is somewhat larger is not surprising for this kind of systems. In fact, as shown previously by some of us for the $CrDy_6$ wheels [4, 5],

magnetic relaxation in systems with a toroidal ground state formally resulting from the ferrotoroidic coupling of an upper and a lower toroidal moiety (imagine here the 5 Dy ions living above (below) the median plane as the upper (lower) toroidal moiety), typically involve a dense cascade of excited magnetic states with very large magnetic moments giving rise to a dense succession of level crossings mediating the population transfer between the maximally magnetic states, stable at large values of the sweeping magnetic field. The dense character of the magnetic excitations (see also Figure 2c), some of which very low-lying in energy, can thus explain the apparent absence in the single-crystal dynamical magnetization profile of any signature of the non-magnetic zero-field ground state with maximal toroidal moment. This picture is also consistent with the smooth character of the observed dynamical magnetic hysteresis in Figure S4 when compared to the steep nature of the simulated low-temperature equilibrium magnetization in Figure 5a, and with the somewhat larger cohercive fields observed on average in the dynamical experiments.

6 Supplementary Note 6: Calculation of the Magnetoelectric Effect Tensor in $Fe_{10}Dy_{10}$

To probe the possibility of manipulating spin degrees of freedom via electric field (spin-electric effect) we need to investigate the energy variation of the target system ($Fe_{10}Dy_{10}$ in this case) to the combined application of an electric and a magnetic field, and isolate the various order of polynomial dependence of such response on the components of the applied fields. This defines a hierarchy of magneto-electric tensors, i.e. mixed derivatives of the ensuing energy change with respect to the components of the external electric and magnetic fields. Such tensors provide a quantitative description of the ensuing Magneto Electric Effect (MEE).

In particular, a system with a non-zero toroidal polarization is expected to display a *linear* magneto-electric tensor, i.e. spin-electric coupling in the lowest possible order of perturbation with respect to electric and magnetic fields, hence the strongest MEE possible [6, 7]. In polynuclear Rare-Earth quantum magnets such as SMTs, coupling to an electric field can only occur in non-centrosymmetric systems, in principle via either single-ion or pair-ion mechanisms. In single-ion mechanisms the electric field can couple local 4f states of each single Ln(III) ion to the 5d manifold of the same ion, via the odd-harmonics of the effective crystal field, like in the effective Judd-Ofelt theory used to rationalise the spectroscopic properties of lanthanide materials. In pair mechanisms of electric polarization, on the other hand, the electric field causes displacement of neighbouring Ln(III) ions which, in turn, modulates the exchange coupling energy, in what is known as an electrostrictive mechanism [7]. However as argued in [7], while in transition metal d-ions electrostriction is a dominant mechanism, in rare earth systems due to the the much weaker 4f-4f exchange interactions the dominant mechanism is by far the single-ion mechanism, so here we will set up a model based on the single-ion electric polarization mechanism.

Our approach here to the approximate calculation of MEE tensors while similar to that presented in [7], presents some significant differences. In that work in fact, like in standard Judd-Ofelt theory, extensive use of coefficients of fractional parentage is made to account for the specificity of the transitions between 4f and 5d spaces, which makes the calculations somewhat more intricate with no gain in accuracy. Moreover, in the model presented in [7] the focus is only on crystal field odd harmonic tensor operators of rank one (thus neglecting contributions from ranks three and five), and the ensuing electric potential is evaluated using an idealised symmetric triangular model that is not possible in the present much less symmetric case, and which neglects altogether the contributions from charges representing the ligand environment.

Here, on the other hand, we build a similar model showing that it is possible to avoid coefficients of fractional parentage if the focus is solely on the calculation of matrix elements of the electric dipole operator within the 4f-ground spin-orbit multiplet derived from the Hund's rule ground Russel-Saunders term of the ion. Moreover, we include in the model all odd harmonics compatible with the development of an effective Hamiltonian acting within the 4f quasi-degenerate ground $2J+1$-manifold of the multiplet $^{2S+1}L_J$. Finally, in our expanded model we calculate the the multi-ion effects both by accounting for effective point charges distributed over the full $Fe_{10}Dy_{10}$

nanoring in the spirit of the Magellan approach to the electrostatic minimization of the single-ion magnetic anisotropy energy [8], and also by projecting the single-ion results on the target polynuclear toroidal wavefunctions, under further perturbation by an external magnetic field.

6.1 The effective single-ion electric Hamiltonian

Since we will build here an effective theory for the open-shell electrons only, let us consider a perturbative electric field Hamiltonian acting on the N electrons occupying the 4f-shell of a single lanthande ion, as:

$$V = V_1 + V_2 \tag{S1}$$

consisting of two terms, both of which have in fact null matrix elements if evaluated using multielectronic states arising from the $4f^N$ configuration only, due to the fact that both V_1 and V_2 are odd under a parity transformation.

In particular, the first term, V_1, describes the coupling of the 4f electrons to an external electric field $\boldsymbol{E}^{\text{ext}}$ via the electric dipole moment of the 4f electrons $\boldsymbol{\mu} = \{\mu_1, \mu_0, \mu_{-1}\}$, where the spherical components of the electric dipole are $\mu_1 = -e\sum_{i=1}^{N} r_i C_1^{(1)}(\mathbf{r_i})$, $\mu_0 = -e\sum_{i=1}^{N} r_i C_0^{(1)}(\mathbf{r_i})$, and $\mu_{-1} = -e\sum_{i=1}^{N} r_i C_{-1}^{(1)}(\mathbf{r_i})$, where $C_q^{(1)}(\mathbf{r_i})$ are the Wybourne irreducible first-rank tensor operators built from the position of the ith 4f-electron, defined in terms of the Spherical Harmonics functions Y_{kq} as $C_q^{(k)}(\mathbf{r}_i) = \sqrt{\frac{4\pi}{2k+1}} Y_{kq}(\theta_i, \phi_i)$. The full Hamiltonian $V_1 = -\boldsymbol{\mu} \cdot \boldsymbol{E}^{\text{ext}}$ thus reads:

$$V_1 = e \sum_{i=1}^{N} \sum_{q=-1}^{+1} (-1)^q E_{-q}^{\text{ext}} r_i C_q^{(1)}(\mathbf{r_i}) \tag{S2}$$

where the spherical components of the electric field vector read $E_1^{\text{ext}} = -2^{-1/2}(E_x^{\text{ext}} + iE_y^{\text{ext}})$, $E_{-1}^{\text{ext}} = 2^{-1/2}(E_x^{\text{ext}} - iE_y^{\text{ext}})$, and $E_0^{\text{ext}} = E_z^{\text{ext}}$.

The second term, V_2, describes the odd harmonics component of the crystal field potential:

$$V_2 = \sum_{k}^{1,3,5} \sum_{q=-k}^{+k} A_{kq}^{\text{CF}} \sum_{i=1}^{N} r_i^k C_q^{(k)}(\mathbf{r_i}) \tag{S3}$$

where, for the case of a crystal field modelled as an external potential generated by a collection of N_{ch} point charges representing the molecular environment (ligands and other metal ions), the expansion coefficients A_{kq} consist of the kth- derivative with respect to the charge's positions of the simple Coulomb potential generated by the N_{ch} point charges evaluated at the position of the lanthanide ion, and is given by the well-known expression:

$$A_{kq}^{\text{CF}} = e \sum_{n=1}^{N_{ch}} \frac{Q_n}{R_n^{k+1}} C_q^{(k)^*}(\mathbf{R_n}) \tag{S4}$$

where $\boldsymbol{R}_n$ is the position of the nth point charge eQ_n.

Degenerate perturbation theory is best expressed in terms of the second quantization formalism. Since V_1 and V_2 are both first quantization one-electron operators, we can write their second quantization counterparts $\hat{V}_1$ and $\hat{V}_2$ as:

$$\hat{V}_i = \sum_{b,c} v_{bc}^{(i)} a_b^\dagger a_c, \quad i = 1, 2 \tag{S5}$$

where $a_b^\dagger$ (a_c) are the creation (annihilation) operators varying the occupation number of the associated one-electron spin-orbital basis $\{\phi_b\}$, while the one-electron matrix elements $v_{bc}^{(1)}$ read:

$$v_{bc}^{(1)} = \sum_{q=-1}^{+1} (-1)^q E_{-q}^{\text{ext}} \left\langle \phi_b \left| r C_q^{(1)}(\mathbf{r}) \right| \phi_c \right\rangle, \tag{S6}$$

and, likewise, the matrix elements $v_{bc}^{(2)}$ read:

$$v_{bc}^{(2)} = \sum_{k}^{1,3,5} \sum_{q=-k}^{+k} A_{kq}^{\text{CF}} \left\langle \phi_b \left| r^k C_q^{(k)}(\mathbf{r}) \right| \phi_c \right\rangle \tag{S7}$$

.

The operators $\hat{V}_i$ couple the 4f space to angular momentum shells of opposite parity i.e. with an even angular momentum quantum number, and included in the Clebsh-Gordan triangular rule. So, in particular, $\hat{V}_1$ and the $k = 1$ component of $\hat{V}_2$ can couple f orbitals to d or g orbitals, while the $k = 3$ ($k = 5$) component of $\hat{V}_2$ can couple the f space to s, d, g, i (d, g, i, k) angular momentum shells. Since the $5d$ excited states are the closest in energy to the $4f$ states in lanthanides, we will limit our model to describe the coupling between $4f$ and $5d$ shells.

Next, we use quasi-degenerate perturbation theory to construct an effective Hamiltonian in the spirit of Judd–Ofelt theory. Approximating the excitation energies from the $4f$ states to the $5d$ states by a single average gap W_{fd}, and retaining only the cross terms between $\hat{V}_1$ and $\hat{V}_2$ in order to remain linear in the electric field, one obtains:

$$\hat{H}_{\text{eff}} = -\frac{1}{W_{fd}} \left(\hat{P}_0 \hat{V}_1 \hat{Q}_{fd} \hat{V}_2 \hat{P}_0 + \hat{P}_0 \hat{V}_2 \hat{Q}_{fd} \hat{V}_1 \hat{P}_0 \right). \tag{S8}$$

Within the truncated configuration space $4f^N \bigoplus 4f^{N-1}5d^1$ considered here, we define the effective identity operator as $\hat{I} = \hat{P}_0 + \hat{Q}_{fd}$. The projector onto the model space is then $\hat{P}_0 \equiv \hat{P}_{4f^N} = \hat{P}_f(N)\hat{P}_d(0)$, while its complement is $\hat{Q}_{fd} \equiv \hat{P}_{4f^{N-1}5d} = \hat{I} - \hat{P}_0 = \hat{P}_f(N-1)\hat{P}_d(1)$. Here we have introduced the projectors $\hat{P}_\ell(n)$ onto fixed occupation-number sectors of the angular momentum shell ℓ occupied by n electrons. These projectors are expressed as polynomials in the number operator $\hat{N}_\ell = \sum_{b\in\ell} a_b^\dagger a_b$, constructed as products of factors $\left(\hat{N}_\ell - m\hat{I}\right)$ that annihilate all eigenspaces except

the one with eigenvalue n, namely:

$$\hat{P}_\ell(n) = \prod_{m=0, m\neq n}^{g_\ell} \left\{ \frac{\hat{N}_\ell - m\hat{I}}{n-m} \right\}$$

where the product runs over all possible electron occupation sectors of the shell ℓ, i.e. $g_\ell = \{0, 1, 2, \ldots, 2(2\ell+1)\}$, except for n.

Using the relations $[\hat{N}_\ell, a_b^\dagger] = a_b^\dagger$ and $[\hat{N}_\ell, a_b] = -a_b$, valid for creation and annihilation operators $a_b^\dagger$ and a_b with $b \in \ell$, one readily obtains

$$\hat{P}_\ell(n)\, a_b^\dagger = a_b^\dagger\, \hat{P}_\ell(n-1), \qquad \hat{P}_\ell(n)\, a_b = a_b\, \hat{P}_\ell(n+1), \tag{S9}$$

reflecting the unit change in occupation number induced by $a_b^\dagger$ and a_b.

We now proceed to eliminate the intermediate-space projector $\hat{Q}_{fd}$ explicitly. Focusing on the first term in Eq. (S8), and considering that the operators $P_\ell(n)$ are idempotent and commute with each other:

$$\hat{P}_0 \hat{V}_1 \hat{Q}_{fd} \hat{V}_2 \hat{P}_0 = \hat{P}_f(N)\hat{P}_d(0)\, \hat{V}_1\, \hat{P}_f(N-1)\hat{P}_d(1)\, \hat{V}_2\, \hat{P}_d(0)\hat{P}_f(N), \tag{S10}$$

Taking a generic excitation operator $a_b^\dagger a_c$ making up the operator $\hat{V}_1$, whose explicit expression in second quantisation is given by Eq (S5), one easily obtains, using shift commutation relationships for $\hat{P}_\ell(n)$ (Eq. (S9)), that the only surviving terms in the sum over spin-orbital indices b and c in Eq. (S5) are:

$$\begin{aligned}
\hat{P}_f(N)\hat{P}_d(0)a_b^\dagger a_c \hat{P}_f(N-1)\hat{P}_d(1) &= \hat{P}_d(0)\hat{P}_f(N)\left[\hat{P}_f(N)a_{b\in 4f}^\dagger\right]\left[a_{c\in 5d}\hat{P}_d(1)\right]\hat{P}_d(1)\hat{P}_f(N-1) \\
&= \hat{P}_0\left[a_{b\in 4f}^\dagger \hat{P}_f(N-1)\right]\left[\hat{P}_d(0)a_{c\in 5d}\right]\hat{P}_f(N-1)\hat{P}_d(1) \\
&= \hat{P}_0\left[a_{b\in 4f}^\dagger \hat{P}_d(0)\hat{P}_f(N-1)a_{c\in 5d}\right]\hat{P}_f(N-1)\hat{P}_d(1) \\
&= \hat{P}_0\ \left[a_{b\in 4f}^\dagger a_{c\in 5d}\right]\hat{P}_f(N-1)\hat{P}_d(1)
\end{aligned}$$

Likewise, for $\hat{V}_2$ in the first term of Eq (S5), one obtains:

$$\hat{P}_f(N-1)\hat{P}_d(1)a_b^\dagger a_c \hat{P}_f(N)\hat{P}_d(0) = \hat{P}_f(N-1)\hat{P}_d(1)\ \left[a_{b\in 5d}^\dagger a_{c\in 4f}\right]\hat{P}_0$$

.

Once such restrictions are imposed on the range of summation over creation and annihilation operators, for this particular truncated space we can proceed to the elimination of $\hat{Q}_{fd}$ and obtain:

$$\hat{H}_{eff} = -\frac{1}{W_{fd}}\hat{P}_0\left(\hat{V}_1^- \hat{V}_2^+ + \hat{V}_2^- \hat{V}_1^+\right)\hat{P}_0, \tag{S11}$$

where:

$$\hat{V}_i^{(-)} = \sum_{\sigma}^{\alpha,\beta} \sum_{m_f \in 4f} \sum_{m_d \in 5d} v^{(i)}_{m_f m_d}\, a^{\dagger}_{m_f,\sigma} a_{m_d,\sigma}$$

$$\hat{V}_i^{(+)} = \sum_{\sigma}^{\alpha,\beta} \sum_{m_f \in 4f} \sum_{m_d \in 5d} v^{(i)}_{m_d m_f}\, a^{\dagger}_{m_d,\sigma} a_{m_f,\sigma}$$

We can now proceed to derive explicitly the resulting effective one-electron operator acting solely within the $4f^N$ subspace, noting that:

$$\begin{aligned}
\hat{V}_1^{(-)}\hat{V}_2^{(+)} &= \sum_{\sigma_1,\sigma_2} \sum_{m_1,m_2 \in 4f} \sum_{m_{d_a},m_{d_b} \in 5d} v^{(1)}_{m_1 m_{d_a}}\, v^{(2)}_{m_{d_b} m_2}\, a^{\dagger}_{m_1,\sigma_1} a_{m_{d_a},\sigma_1} a^{\dagger}_{m_{d_b},\sigma_2} a_{m_2,\sigma_2} \\
&= \sum_{\sigma_1,\sigma_2} \sum_{m_1,m_2} \sum_{m_{d_a},m_{d_b}} v^{(1)}_{m_1 m_{d_a}}\, v^{(2)}_{m_{d_b} m_2}\, a^{\dagger}_{m_1,\sigma_1} \left(\delta_{m_{d_a},m_{d_b}} \delta_{\sigma_1,\sigma_2} - a^{\dagger}_{m_{d_b},\sigma_2} a_{m_{d_a},\sigma_1}\right) a_{m_2,\sigma_2} \\
&= \sum_{\sigma} \sum_{m_1,m_2 \in 4f} \left(\sum_{m_d \in 5d} v^{(1)}_{m_1 m_d}\, v^{(2)}_{m_d m_2} \right) a^{\dagger}_{m_1,\sigma} a_{m_2,\sigma} + \hat{O}_2.
\end{aligned}$$

The result is an effective one-electron operator acting solely within the $4f^N$ model space, corrected by a bielectronic term $\hat{O}_2$ that generates excitations solely within the $4f^{N-1}5d$ space, so that all terms $\hat{P}_0 a^{\dagger}_{m_1,\sigma_1} a^{\dagger}_{m_{d_b},\sigma_2} a_{m_{d_a},\sigma_1} a_{m_2,\sigma_2} \hat{P}_0$ are clearly zero.

Since the perturbations Eq. (S2) and (S3) are spin-independent, we can also factor out the sum over the spin degrees of freedom, by defining the singlet excitation operators $\hat{E}_{m_1 m_2} = a^{\dagger}_{m_1,\alpha} a_{m_2,\alpha} + a^{\dagger}_{m_1,\beta} a_{m_2,\beta}$, and write the final second-order effective Hamiltonian describing the linear coupling of the $4f^N$ space to an external electric field as:

$$\hat{H}_{\text{eff}} = \sum_{m_1,m_2 \in 4f} G_{m_1,m_2} \hat{E}_{m_1 m_2} \tag{S12}$$

where the integrals representing the effective one-electron operator within the $4f^N$ model space, and containing all information about the coupling to the integrated out $4f^{N-1}5d$ space, read:

$$G_{m_1,m_2} = -\frac{1}{W_{fd}} \sum_{m_d} \left(v^{(1)}_{m_1,m_d} v^{(2)}_{m_d,m_2} + v^{(2)}_{m_1,m_d} v^{(1)}_{m_d,m_2} \right). \tag{S13}$$

6.1.1 Electric Hamiltonian in the coupled Irreducible Tensor Representation

Starting from Eq. (S13) for the effective one-electron operator within the uncoupled $4f$ basis, and using the explicit expressions for the intervening matrix elements (S6)

and (S7), we write

$$G_{m_1 m_2} = \sum_{\mu=-1}^{+1} (-1)^\mu E^{\rm ext}_{-\mu} \sum_{g=1,3,5} \sum_{\gamma=-g}^{+g} G^{(g)}_{fd} A^{\rm CF}_{g\gamma} \; \left(O^{1,g}_{\mu\gamma}(m_1, m_2) + O^{g,1}_{\gamma\mu}(m_1, m_2) \right), \tag{S14}$$

where

$$G^{(g)}_{fd} = -e^2 \frac{\langle r \rangle_{fd} \langle r^g \rangle_{fd}}{W_{fd}} \tag{S15}$$

is the effective coupling constant associated with the product of the radial transition dipole $\langle r \rangle_{fd}$ and the radial transition multipoles $\langle r^g \rangle_{fd}$ associated to the odd crystal harmonics of rank $g = 1, 3, 5$, divided by the average excitation energy gap W_{fd} between $4f$ and $5d$ one-electron states, and

$$O^{1,g}_{\mu\gamma}(m_1, m_2) = \sum_{m_d=-2}^{+2} \langle 3m_1 | C^{(1)}_\mu | 2m_d \rangle \; \langle 2m_d | C^{(g)}_\gamma | 3m_2 \rangle \,, \tag{S16}$$

with an analogous definition for $O^{g,1}_{\gamma\mu}(m_1, m_2)$.

Focusing first on the ordered contribution $O^{1,g}_{\mu\gamma}$, application of the Wigner–Eckart theorem gives

$$\begin{aligned} O^{1,g}_{\mu\gamma}(m_1, m_2) &= \langle 3 \| C^{(1)} \| 2 \rangle \, \langle 2 \| C^{(g)} \| 3 \rangle \sum_{m_d=-2}^{+2} (-1)^{2-m_d} \begin{pmatrix} 3 & 1 & 2 \\ -m_1 & \mu & m_d \end{pmatrix} \begin{pmatrix} 2 & g & 3 \\ -m_d & \gamma & m_2 \end{pmatrix} \\ &= \sqrt{3} \, \langle 2 \| C^{(g)} \| 3 \rangle \, \frac{(-1)^{m_1+g}}{\sqrt{35}} \sum_{m_d} \langle 1 \, \mu \, 2 \, m_d | 3 \, m_1 \rangle \langle g \, \gamma \, 3 \, m_2 | 2 \, m_d \rangle. \end{aligned} \tag{S17}$$

Using the Racah recoupling identity [9]:

$$\sum_{m_d} \langle 1 \, \mu \, 2 \, m_d | 3 \, m_1 \rangle \langle g \, \gamma \, 3 \, m_2 | 2 \, m_d \rangle = \sum_{KQ} \begin{Bmatrix} 1 & g & K \\ 3 & 3 & 2 \end{Bmatrix} \langle 1 \, \mu \, g \, \gamma | K \, Q \rangle \langle K \, Q \, 3 \, m_2 | 3 \, m_1 \rangle$$

Eq. (S17) may be rewritten as

$$O^{1,g}_{\mu\gamma}(m_1, m_2) = \sum_K \Omega^{(K)}_g \sum_{Q=-K}^{+K} \langle 1 \, \mu \, g \, \gamma | K \, Q \rangle (-1)^{3-m_1} \begin{pmatrix} 3 & K & 3 \\ -m_1 & Q & m_2 \end{pmatrix}, \tag{S18}$$

where the coefficients $\Omega^{(K)}_g$ collect the reduced one-electron matrix elements and the corresponding $6j$ recoupling factors. For $g = 1, 3, 5$, the allowed values of the coupled rank are those generated by the Clebsch–Gordan series $1 \otimes g$, namely $K = 0, \ldots, 6$; in the following we retain only the anisotropic even-rank contributions $K = 2, 4, 6$, while the scalar $K = 0$ term merely produces a uniform energy shift.

Substituting this result back into the effective Hamiltonian, and coupling the singlet excitation operators

$$\hat{E}_{m_1 m_2} = a^\dagger_{m_1,\alpha} a_{m_2,\alpha} + a^\dagger_{m_1,\beta} a_{m_2,\beta} \tag{S19}$$

into irreducible tensor operators,

$$\hat{U}^{(K)}_Q = \sum_{m_1,m_2} (-1)^{3-m_1} \begin{pmatrix} 3 & K & 3 \\ -m_1 & Q & m_2 \end{pmatrix} \hat{E}_{m_1 m_2}, \tag{S20}$$

the effective electric Hamiltonian can be cast in the form

$$\hat{H}_{\text{eff}} = \sum_{K=2,4,6} \sum_{Q=-K}^{+K} B^{(12)}_{KQ} \hat{U}^{(K)}_Q, \tag{S21}$$

with

$$B^{(12)}_{KQ} = \sum_{\mu=-1}^{+1} (-1)^\mu E^{\text{ext}}_{-\mu}\, b^{K,(12)}_{Q\mu}, \tag{S22}$$

and

$$b^{K,(12)}_{Q\mu} = \sum_{g=1,3,5} G^{(g)}_{fd} \Omega^{(K)}_g \sum_{\gamma=-g}^{+g} A^{\text{CF}}_{g\gamma}\, \langle 1\, \mu\, g\, \gamma | K\, Q \rangle. \tag{S23}$$

For the present case, the coefficients $b^{K,(12)}_{Q\mu}$ satisfy the tensor reality condition

$$\left(b^{K,(12)}_{Q\mu} \right)^* = (-1)^{Q+\mu}\, b^{K,(12)}_{-Q,-\mu}, \tag{S24}$$

which follows from the reality properties of the crystal-field coefficients $A^{\text{CF}}_{g\gamma}$ and of the Clebsch–Gordan coefficients. As a consequence, the Hermitian effective Hamiltonian may be written simply as

$$\hat{H}^{(\text{el})}_{\text{eff}} = 2 \sum_{K=2,4,6} \sum_{Q=-K}^{+K} B^{(12)}_{KQ} \hat{U}^{(K)}_Q. \tag{S25}$$

6.1.2 Matrix elements of the electric Hamiltonian within the ground spin-orbit multiplet

The aim here is to estimate the spin-electric coupling and the ensuing magnetoelectric effect. We are therefore interested only in the electric-field dependence of the magnetic excitations of the lanthanide ionic centre embedded in the molecular crystal-field potential, i.e. of the states arising from the crystal-field splitting of the lowest-energy J multiplet ${}^{2S+1}L_J$, where ${}^{2S+1}L$ is the ground Russell–Saunders term and, according to Hund's rules, $L = L_{\text{max}}$ and $S = S_{\text{max}}$ are the maximum values compatible with the occupation of the $4f$ shell. The only matrix elements that are relevant at

this stage are therefore those within the $(2J+1)$-dimensional manifold spanned by the N-electron states $|4f^N LS, JM\rangle$, with $M = -J, -J+1, \ldots, J-1, J$.

Since the operators $\hat{U}_Q^{(K)}$ act only on the orbital part of the $4f^N$ wave functions, their matrix elements between the states $|4f^N LS, JM\rangle$ are obtained by first applying the Wigner–Eckart theorem at the J level,

$$\left\langle 4f^N LS; J, M_1 \middle| \hat{U}_Q^{(K)} \middle| 4f^N LS; J, M_2 \right\rangle = (-1)^{J-M_1} \begin{pmatrix} J & K & J \\ -M_1 & Q & M_2 \end{pmatrix} \langle 4f^N LSJ \| \hat{U}^{(K)} \| 4f^N LSJ \rangle \,, \tag{S26}$$

and then reducing the corresponding matrix element from the J-coupled basis to the orbital L space.

Because $\hat{U}^{(K)}$ is a purely orbital operator, standard recoupling theory gives

$$\langle 4f^N LSJ \| \hat{U}^{(K)} \| 4f^N LSJ \rangle = (-1)^{L+S+J+K} (2J+1) \begin{Bmatrix} L & J & S \\ J & L & K \end{Bmatrix} \langle 4f^N L \| \hat{U}^{(K)} \| 4f^N L \rangle \,. \tag{S27}$$

The remaining reduced matrix element in the orbital space may in turn be evaluated by exploiting the fact that the ground Russell–Saunders term corresponds to the maximum orbital angular momentum $L = L_{\max}$. In this case, $\langle 4f^N L \| \hat{U}^{(K)} \| 4f^N L \rangle$ is completely determined by the expectation value of the $Q = 0$ component on the stretched orbital state $|4f^N, L, M_L = L\rangle$, namely

$$\langle 4f^N L \| \hat{U}^{(K)} \| 4f^N L \rangle = \frac{\left\langle 4f^N, L, L \middle| \hat{U}_0^{(K)} \middle| 4f^N, L, L \right\rangle}{\begin{pmatrix} L & K & L \\ -L & 0 & L \end{pmatrix}}. \tag{S28}$$

For the maximum-L state, the spatial part of the wave function is represented by a single Slater determinant, so that only diagonal one-electron occupations contribute. Using the definition of $\hat{U}_Q^{(K)}$ in terms of singlet excitation operators, one finds

$$\left\langle 4f^N, L, L \middle| \hat{U}_0^{(K)} \middle| 4f^N, L, L \right\rangle = \sum_{m=-3}^{3} (-1)^{3-m} \begin{pmatrix} 3 & K & 3 \\ -m & 0 & m \end{pmatrix} n_m^{(L)}, \tag{S29}$$

where $n_m^{(L)}$ is the occupation number of the orbital with magnetic quantum number m in the stretched orbital configuration generating the term $L = L_{\max}$. For the Hund's-rule maximum-L state relevant here, these occupation numbers are fixed by the single-determinant structure of the orbital part of the wave function.

Collecting the above results, the matrix elements of the effective Hamiltonian within the ground spin–orbit multiplet may be written as

$$\left\langle 4f^N LS; J, M_1 \middle| \hat{H}_{\text{eff}} \middle| 4f^N LS; J, M_2 \right\rangle = \sum_{K=2,4,6} \eta_{LSJ}^{(K)} \sum_{Q=-K}^{K} B_{KQ}^{(12)} (-1)^{J-M_1} \begin{pmatrix} J & K & J \\ -M_1 & Q & M_2 \end{pmatrix}, \tag{S30}$$

where

$$\eta_{LSJ}^{(K)} = (-1)^{L+S+J+K}(2J+1)\begin{Bmatrix} L & J & S \\ J & L & K \end{Bmatrix} \frac{\sum_{m=-3}^{3}(-1)^{3-m}\begin{pmatrix} 3 & K & 3 \\ -m & 0 & m \end{pmatrix} n_m^{(L)}}{\begin{pmatrix} L & K & L \\ -L & 0 & L \end{pmatrix}}. \quad \text{(S31)}$$

For the purpose of constructing the magnetoelectric response tensor, it is instrumental to rewrite the effective electric Hamiltonian in a form where the external electric field components are explicitly factorised in a local Cartesian frame attached to each Dy(III) ion.

As discussed above, the effective Hamiltonian for the single Dy(III) site originates from the coupling between the external electric field with components calculated in the local ion's reference frame with $z_{\rm loc}$ axis given by the local magnetic anisotropy easy-axis, and the twentyone spherical components of the odd-parity crystal field generated by the surrounding charge distribution, mediated by $4f$–$5d$ mixing, also calculated with respect to the same local Cartesian reference frame. To express all this we will add from now on an extra label I labelling the specific Dy(III) centre among the ten centres available in $Fe_{10}Dy_{10}$. Hence, after projection onto the ground spin–orbit multiplet, the local effective electric Hamiltonian for the Ith Dy(III) ion can be expressed as a linear function of the external electric field as:

$$\hat{H}_{\rm eff}^{(\rm el)}(I) = \sum_{\alpha=x,y,z} E_\alpha^{\rm ext,loc}(I)\,\hat{P}_\alpha(I), \quad \text{(S32)}$$

where α denotes the Cartesian components defined in the local magnetic frame of the Ith ion.

The operators $\hat{P}_\alpha(I)$ represent the effective electric-dipole operators acting within the ground multiplet $^6H_{15/2}$, and are obtained by combining the spherical tensor components derived in the previous section. Explicitly, they can be written as

$$\hat{P}_\alpha(I) = \sum_{K=2,4,6}\sum_{Q=-K}^{K} C_{\alpha,KQ}(I)\,\hat{U}_Q^{(K)}, \quad \text{(S33)}$$

where the coefficients $C_{\alpha,KQ}(I)$ are:

$$C_{x,KQ}(I) = \sqrt{2}\left(b_{Q,-1}^{K,(12)}(I) - b_{Q,+1}^{K,(12)}(I)\right), \quad \text{(S34)}$$

$$C_{y,KQ}(I) = i\sqrt{2}\left(b_{Q,-1}^{K,(12)}(I) + b_{Q,+1}^{K,(12)}(I)\right), \quad \text{(S35)}$$

$$C_{z,KQ}(I) = 2\,b_{Q,0}^{K,(12)}(I). \quad \text{(S36)}$$

These relations follow from the standard transformation between spherical ($\mu = -1, 0, +1$) and Cartesian components of a vector operator, and they collect all microscopic contributions, including: (i) the odd crystal-field parameters generated by the surrounding charges, (ii) the radial matrix elements associated with $4f$–$5d$ mixing, and (iii) the angular coupling coefficients arising from tensor operator algebra.

In practice, the operators $\hat{P}_\alpha(I)$ are constructed numerically in the $|J, M_J\rangle$ basis of the ground spin–orbit multiplet, using the ab initio single-ion wavefunctions described below.

This Cartesian form provides the natural starting point for the derivation of the magnetoelectric response tensor, since it makes explicit the linear coupling between the external electric field and the effective dipole operators within the low-energy manifold.

6.2 Local and Global Magnetoelectric tensors

In the local magnetic frame of each Dy(III) centre I, the magnetic interaction within the ground spin–orbit multiplet is described by

$$\hat{H}_{\text{mag}}(I) = -\hat{\mathbf{M}}(I) \cdot \mathbf{B}^{\text{ext,loc}}(I), \tag{S37}$$

where $\hat{\mathbf{M}}(I) = -\mu_B g_J \hat{\boldsymbol{J}}$ is expressed in the $|J, M_J\rangle$ local basis defined by the ab initio single-ion anisotropy axes for the Ith ion, g_J the Landé g-factor and μ_B is the Bohr magneton.

Starting from the effective electric Hamiltonian introduced in Eqs. (S32), the magnetoelectric response is obtained from second-order perturbation theory by combining electric and magnetic interactions within the ground spin–orbit multiplet.

The local magnetoelectric tensor is evaluated within the ab initio ground Kramers doublet manifold of a single Dy(III) ion, whose two partners are denoted $|+\text{KD}_0\rangle$ and $|-\text{KD}_0\rangle$. Labeling the excited crystal field KDs generically as $|\text{KD}_n\rangle$, the corresponding local magnetoelectric tensor is obtained as:

$$\alpha_{\alpha\beta}^{\text{ME},\pm}(I) = -\sum_{n\neq 0}\left[\frac{\langle \pm\text{KD}_0|\hat{P}_\alpha(I)|\text{KD}_n\rangle\langle \text{KD}_n|\hat{M}_\beta(I)|\pm\text{KD}_0\rangle}{E_n - E_0} + \text{h.c.}\right], \tag{S38}$$

where $\alpha, \beta = x, y, z$ denote Cartesian components in the local frame with z-axis along the local principal magnetic easy-axis, and where h.c. denotes the Hermitian-conjugate contribution obtained by exchanging $\hat{P}_\alpha(I)$ and $\hat{M}_\beta(I)$.

This expression corresponds to the diagonal ($\pm \to \pm$) response of the Kramers doublet. The tensor associated with the time-reversal partner satisfies

$$\alpha_{\alpha\beta}^{\text{ME},+} = -\alpha_{\alpha\beta}^{\text{ME},-}, \tag{S39}$$

so that the two toroidal ground states correspond to opposite magnetoelectric responses, and in the absence of splitting, there is no net magnetoelectric response.

The local tensors defined above provide the building blocks for the collective magnetoelectric response of the $Fe_{10}Dy_{10}$ wheel. For a given collective Ising

configuration

$$\boldsymbol{\sigma} = (\sigma_1, \sigma_2, \ldots, \sigma_{10}), \qquad \sigma_I = \pm 1,$$

we approximate the corresponding low-energy collective state as a direct product of local ground Kramers-doublet partners,

$$|\boldsymbol{\sigma}\rangle = \prod_{I=1}^{10} |\sigma_I(\mathrm{KD}_0)\rangle. \tag{S40}$$

The excited states entering the perturbative magnetoelectric response are approximated as local crystal-field excitations on top of the same Ising background,

$$|n; I, e\rangle = |\sigma_1(\mathrm{KD}_0)\rangle \cdots |\sigma_I(\mathrm{KD}_e)\rangle \cdots |\sigma_{10}(\mathrm{KD}_0)\rangle, \tag{S41}$$

where KD_e denotes an excited Kramers doublet of the Ith Dy(III) ion.

With these definitions, the collective-state-dependent magnetoelectric tensor can formally be written as

$$\alpha^{\mathrm{ME}}_{\alpha\beta}(\boldsymbol{\sigma}) = -\sum_{I,e} \left[\frac{\langle \boldsymbol{\sigma}|\hat{P}_\alpha|n; I, e\rangle\langle n; I, e|\hat{M}_\beta|\boldsymbol{\sigma}\rangle}{E_{I,e} - E_{\boldsymbol{\sigma}}} + \mathrm{h.c.} \right]. \tag{S42}$$

Since the local crystal-field excitation energies are much larger than the splittings within the low-energy Ising manifold, the denominators are approximated by the corresponding local excitation energies. The expression then reduces to a sum of local contributions whose sign is determined by the orientation of each local Ising moment,

$$\boldsymbol{\alpha}^{\mathrm{ME}}(\boldsymbol{\sigma}) = \sum_{I=1}^{10} \sigma_I \, R_I \, \boldsymbol{\alpha}^{\mathrm{ME}}(I) \, R_I^T. \tag{S43}$$

Here R_I rotates the local magnetic reference frame of the Ith Dy(III) ion into the common molecular inertia frame, defined by the ab initio local g-tensor principal axes. This is the state-resolved tensor used in the nonequilibrium magnetoelectric readout calculation described in the main text.

For physical interpretation, the tensor can be decomposed into irreducible contributions:

$$\alpha^{\mathrm{ME}}_{\alpha\beta} = \frac{1}{3}\mathrm{Tr}(\alpha^{\mathrm{ME}})\,\delta_{\alpha\beta} + \alpha^{\mathrm{ME,sym}}_{\alpha\beta} + \alpha^{\mathrm{ME,anti}}_{\alpha\beta}, \tag{S44}$$

corresponding respectively to the monopolar, symmetric traceless, and antisymmetric (toroidal) components.

The antisymmetric part is equivalently expressed in terms of a dual induced magnetoelectric toroidal vector:

$$\tau^{\mathrm{ME}}_\gamma = \epsilon_{\gamma\alpha\beta}\,\alpha^{\mathrm{ME}}_{\alpha\beta}, \tag{S45}$$

where $\epsilon_{\gamma\alpha\beta}$ is the totally antisymmetric Levi-Civita third-rank tensor, and summation over repeated Greek indices is implied.

Among the collective Ising configurations introduced above, the two maximally toroidal states correspond to the two ground-state Kramers partners of the molecule. In the local frames, the single-ion ground Kramers doublet states are denoted $|+\rangle_I$ and $|-\rangle_I$, corresponding to eigenstates of $\hat{J}_z^{\mathrm{loc}}$ along the local easy axis. The two collective toroidal states are then constructed as

$$|\tau\rangle = |+,+,+,\ldots,+\rangle, \qquad |-\tau\rangle = |-,-,-,\ldots,-\rangle, \tag{S46}$$

where the local quantization axes are chosen consistently along the toroidal direction. These two states are related by time-reversal symmetry and correspond to opposite toroidal polarizations.

In the presence of external electric and magnetic fields, the magnetoelectric interaction energy for a given toroidal state is expressed as

$$E_{\pm\tau}^{\mathrm{ME}} = \sum_{\alpha\beta} E_\alpha \, \alpha_{\alpha\beta}^{\mathrm{ME},\pm\tau} \, B_\beta, \tag{S47}$$

which can be decomposed into irreducible contributions according to the tensor decomposition introduced above.

The monopolar contribution reads

$$E_{\pm\tau}^{\mathrm{mono}} = \frac{1}{3}\mathrm{Tr}(\alpha^{\mathrm{ME},\pm\tau})\,(\mathbf{E}\cdot\mathbf{B}), \tag{S48}$$

while the antisymmetric (toroidal) contribution can be written as

$$E_{\pm\tau}^{\mathrm{anti}} = \boldsymbol{\tau}^{\mathrm{ME},\pm\tau}\cdot(\mathbf{E}\times\mathbf{B}), \tag{S49}$$

where $\boldsymbol{\tau}^{\mathrm{ME}}$ is the magnetoelectric toroidal moment defined as the dual of the antisymmetric tensor. Finally, the symmetric traceless component gives rise to

$$E_{\pm\tau}^{\mathrm{sym}} = \sum_{\alpha\beta} E_\alpha \, \alpha_{\alpha\beta}^{\mathrm{ME,sym},\pm\tau} \, B_\beta, \tag{S50}$$

which includes terms such as $E_x B_y + E_y B_x$ and analogous combinations.

Importantly, all three contributions change sign under time reversal, so that the two toroidal states respond in an opposite manner:

$$E_{-\tau}^{\mathrm{ME}} = -E_{+\tau}^{\mathrm{ME}}, \tag{S51}$$

implying that any of these interactions leads to an energy splitting between the two toroidal states.

We now discuss the observable consequences of the magnetoelectric coupling, and its possible exploitation as a readout mechanism for toroidal states selectively populated and split, e.g., through the pulsed-laser protocol discussed in the previous section.

First, an external electric field induces a magnetic dipole moment according to

$$M_\alpha^{\mathrm{ind}} = \sum_\beta \alpha_{\beta\alpha}^{\mathrm{ME}} E_\beta, \tag{S52}$$

which, for in-plane electric fields, generally results in an induced magnetic moment with a sizeable perpendicular component relative to the applied electric field direction. This electric-to-magnetic conversion constitutes the most direct route for detecting a toroidal polarization in the absence of an externally applied magnetic field.

Conversely, a magnetic field induces an electric dipole moment according to

$$P_\alpha^{\mathrm{ind}} = \sum_\beta \alpha_{\alpha\beta}^{\mathrm{ME}} B_\beta, \tag{S53}$$

providing the reciprocal magnetoelectric response.

Finally, the simultaneous application of electric and magnetic fields produces an energy splitting between the two collective toroidal states,

$$\Delta E = E^{\mathrm{ME}}(|\tau\rangle) - E^{\mathrm{ME}}(|-\tau\rangle) = 2\sum_{\alpha\beta} E_\alpha \, \alpha_{\alpha\beta}^{\mathrm{ME}} B_\beta, \tag{S54}$$

which can be decomposed into monopolar, antisymmetric (toroidal), and symmetric-traceless contributions according to the tensor decomposition introduced above. In particular, the antisymmetric contribution yields a term proportional to $\mathbf{E}\times\mathbf{B}$, while the monopolar and symmetric components contribute to combinations such as $\mathbf{E}\cdot\mathbf{B}$ and $E_xB_y + E_yB_x$.

These expressions parallel those commonly discussed in the context of bulk magnetoelectric materials [6, 7], with the important distinction that here the magnetoelectric response is defined and evaluated at the level of individual molecular Kramers doublets and of their collective toroidal configurations.

6.3 Magnetoelectric tensor: numerical evaluation

The magnetoelectric tensor of the $Fe_{10}Dy_{10}$ wheel was evaluated through our own implementation of the theory developed above in a Mathematica notebook, using a Dy-only approximation and taking as local electronic input the crystal-field-split $^6H_{15/2}$ ground spin–orbit multiplet obtained from our CERES calculations for a representative Dy centre (Dy1), following the methodology described in [10]. In the present implementation, the local magnetic anisotropy axes are those reported for the ground Kramers doublets in Tables (S1-S5), and define the local reference frames used throughout the calculation. The same local $|J, M_J\rangle$ decomposition was transferred to the five symmetry-unique Dy(III) centres, so as to retain the physically relevant crystal-field-induced mixing within the $J = 15/2$ manifold while maintaining the model at a tractable level of complexity.

The sixteen local crystal-field states entering the perturbative summation are partitioned into Kramers-degenerate pairs, and the corresponding $|J, M_J\rangle$ decompositions and excitation energies are reported in Tables S11-S18.

The effective electric dipole operators $\hat{P}_\alpha(I)$, Eq. (S33), and in particular the odd-parity crystal-field terms Eq. (S4) entering the effective $4f$ electric Hamiltonian Eq. (S32) through the coefficients Eq. (S23), were constructed from a point-charge representation of the molecular environment derived from the crystallographic structure. The model includes Dy^{3+}, Fe^{3+}, nitrate groups, and alkoxide oxygens from the $MeTea^{3-}$ and $MeTeaH^{2-}$ ligands, with charges assigned according to standard chemical valence considerations following the same criteria detailed in [8]. For each Dy centre I, all neighbouring charges were first expressed in the common molecular frame and subsequently rotated into the local magnetic frame defined by the corresponding anisotropy axes. The odd crystal-field harmonics entering the effective electric Hamiltonian were therefore evaluated directly in the local frame of each ion, ensuring full consistency between the electric operators $\hat{P}_\alpha(I)$ and the magnetic operators $\hat{M}_\beta(I)$.

The three radial coupling constants Eq. (S15) entering the parity-odd-crystal-field-mediated $4f$–$5d$ coupling were taken as the second-order Judd–Ofelt radial integrals for Dy(III) tabulated by Wybourne and Smentek in Table 21-9 of Ref. [11]. The values used here are $G^{(1)}_{fd} = -0.2949$ a.u., $G^{(3)}_{fd} = -1.0005$ a.u., and $G^{(5)}_{fd} = -4.8183$ a.u.

The resulting state-resolved local magnetoelectric tensors were evaluated from the second-order perturbative expression discussed above, summing over all excited Kramers doublets within the ground $^6H_{15/2}$ manifold. The local tensors were subsequently rotated into a common molecular frame defined by the principal axes of inertia of the Dy ring. Denoting by R_I the rotation matrix whose columns are the local magnetic axes of the Ith Dy ion expressed in the common molecular frame, the contribution of site I reads

$$\alpha^{\text{ME}}_{\text{glob}}(I) = R_I\, \alpha^{\text{ME}}(I)\, R_I^{\text{T}}, \tag{S55}$$

and the total tensor associated with the maximally toroidal state $|\tau\rangle$ is finally obtained as

$$\alpha^{\text{ME,tot}} = \sum_I \alpha^{\text{ME}}_{\text{glob}}(I). \tag{S56}$$

In the inertia frame of the Dy ring, the total magnetoelectric tensor obtained for the toroidal state $|\tau\rangle$ is, in atomic units,

$$\alpha^{\text{ME}}_{\text{raw}} = \begin{pmatrix} 28.185 & -2.10859 & 0.643747 \\ -12.3051 & 14.1911 & 0.156763 \\ 0.984914 & -0.944577 & 8.99839 \end{pmatrix}. \tag{S57}$$

The tensor entries are reported in atomic units, with conversion factor

$$1 \text{ a.u. of } \alpha^{\text{ME}} = 1.8158 \times 10^{-12}\ \text{cm}^{-1}\,\text{T}^{-1}\,\text{V}^{-1}\,\text{m}. \tag{S58}$$

or alternatively, if we express it explicitly also as function of Bohr magneton (μ_B) units:

$$1 \text{ a.u. of } \alpha^{\mathrm{ME}} = 3.8894 \times 10^{-12} \mu_B \, (\mathrm{V/m})^{-1}. \tag{S59}$$

The tensor can be decomposed as

$$\alpha^{\mathrm{ME}}_{\alpha\beta} = \frac{1}{3}\mathrm{Tr}(\alpha^{\mathrm{ME}})\delta_{\alpha\beta} + \alpha^{\mathrm{ME,sym,tr}}_{\alpha\beta} + \alpha^{\mathrm{ME,anti}}_{\alpha\beta}, \tag{S60}$$

where

$$\alpha^{\mathrm{ME,anti}}_{\alpha\beta} = \frac{1}{2}\left(\alpha^{\mathrm{ME}}_{\alpha\beta} - \alpha^{\mathrm{ME}}_{\beta\alpha}\right), \tag{S61}$$

and

$$\alpha^{\mathrm{ME,sym,tr}}_{\alpha\beta} = \frac{1}{2}\left(\alpha^{\mathrm{ME}}_{\alpha\beta} + \alpha^{\mathrm{ME}}_{\beta\alpha}\right) - \frac{1}{3}\mathrm{Tr}(\alpha^{\mathrm{ME}})\delta_{\alpha\beta}. \tag{S62}$$

The monopolar magnetoelectric contribution of the ground molecular toroidal doublet is

$$\frac{1}{3}\mathrm{Tr}(\alpha^{\mathrm{ME}}) = 17.12483. \tag{S63}$$

The antisymmetric contribution of the ground molecular toroidal doublet reads:

$$\alpha^{\mathrm{ME,anti}} = \begin{pmatrix} 0 & 5.09826 & -0.17058 \\ -5.09826 & 0 & 0.55067 \\ 0.17058 & -0.55067 & 0 \end{pmatrix}, \tag{S64}$$

corresponding to the induced magnetoelectric toroidal dual vector

$$\boldsymbol{\tau}^{\mathrm{ME}} = (0.55067,\ 0.170584,\ 5.09826). \tag{S65}$$

Its dominant component lies along the axis normal to the Dy ring plane, as expected for a toroidal state.

Finally, the symmetric traceless contribution is found to be

$$\alpha^{\mathrm{ME,sym,tr}} = \begin{pmatrix} 11.0602 & -7.20685 & 0.814331 \\ -7.20685 & -2.93373 & -0.393907 \\ 0.814331 & -0.393907 & -8.12644 \end{pmatrix}. \tag{S66}$$

These results show that the magnetoelectric response of the realistic $Fe_{10}Dy_{10}$ wheel contains sizeable antisymmetric, monopolar, and symmetric-traceless contributions, reflecting the combined effects of the molecular geometry, crystal-field distribution, and local magnetic-axis arrangement.

Finally, we stress that in the present implementation only the Dy(III) ions are treated as explicit contributors to the magnetoelectric tensor. The Fe(III) centres, although magnetically more isotropic, are expected to experience coherent dipolar and exchange fields generated by the neighbouring Dy moments, and may therefore provide additional contributions to the total molecular response. The present values should thus be regarded as a lower bound to the actual single-molecule magnetoelectric response.

Table S11 $|J, M_J\rangle$ decomposition of the first Kramers doublet of the Dy(III) $^6H_{15/2}$ manifold. Rows correspond to the basis states $|J = 15/2, M_J\rangle$, ordered from $M_J = -15/2$ to $+15/2$. Energies are given in cm^{-1}.

	KD1 ($E = 0.0\,cm^{-1}$)	
M_J	$\|\Psi_1\rangle$	$\|\Psi_2\rangle$
−15/2	$-0.697128 + 0.660839i$	$-0.152035 + 0.144121i$
−13/2	$-0.000532 - 0.021204i$	$0.001897 - 0.005851i$
−11/2	$0.012269 - 0.005714i$	$0.003604 - 0.001697i$
−9/2	$0.030538 - 0.115705i$	$0.004584 - 0.029889i$
−7/2	$-0.083351 - 0.080713i$	$-0.015745 - 0.017436i$
−5/2	$0.022235 - 0.024713i$	$0.003953 + 0.000500i$
−3/2	$0.007950 + 0.028209i$	$0.003239 + 0.014293i$
−1/2	$0.020115 + 0.010867i$	$0.023714 - 0.014291i$
+1/2	$0.027042 - 0.005943i$	$-0.007122 + 0.021725i$
+3/2	$0.007482 + 0.012601i$	$-0.013637 - 0.025942i$
+5/2	$0.002525 - 0.003082i$	$-0.033139 - 0.002639i$
+7/2	$-0.000568 - 0.023486i$	$-0.004964 + 0.115920i$
+9/2	$0.023890 + 0.018538i$	$-0.101764 - 0.062963i$
+11/2	$-0.003783 + 0.001248i$	$0.012835 - 0.004294i$
+13/2	$0.005402 + 0.002942i$	$-0.014202 - 0.015755i$
+15/2	0.209488	-0.960570

Table S12 $|J, M_J\rangle$ decomposition of the second Kramers doublet of the Dy(III) $^6H_{15/2}$ manifold.

	KD2 ($E = 104.9\,cm^{-1}$)	
M_J	$\|\Psi_3\rangle$	$\|\Psi_4\rangle$
−15/2	$-0.004481 - 0.029866i$	$-0.002813 - 0.018753i$
−13/2	$0.643157 - 0.419129i$	$0.022635 - 0.305640i$
−11/2	$0.109247 - 0.281588i$	$-0.006773 - 0.104819i$
−9/2	$-0.110024 - 0.099315i$	$-0.042040 + 0.024577i$
−7/2	$-0.125465 + 0.136838i$	$-0.023386 + 0.054859i$
−5/2	$0.129821 + 0.266745i$	$0.109124 - 0.019248i$
−3/2	$0.086110 + 0.070860i$	$0.068017 + 0.117862i$
−1/2	$0.061729 - 0.073901i$	$-0.093128 - 0.040925i$
+1/2	$-0.054289 - 0.086026i$	$0.063925 - 0.072010i$
+3/2	$-0.126649 - 0.049778i$	$0.082851 + 0.074645i$
+5/2	$-0.002845 + 0.110772i$	$-0.283054 - 0.088809i$
+7/2	$-0.050783 + 0.031266i$	$0.116709 - 0.144378i$
+9/2	$0.018068 - 0.045221i$	$0.114539 + 0.094072i$
+11/2	$0.104664 - 0.008854i$	$-0.262263 + 0.149815i$
+13/2	$-0.298899 + 0.067730i$	$0.319070 - 0.698222i$
+15/2	0.018963	-0.030200

Table S13 $|J, M_J\rangle$ decomposition of the third Kramers doublet of the Dy(III) $^6H_{15/2}$ manifold.

	KD3 ($E = 159.3\,\mathrm{cm}^{-1}$)	
M_J	$\|\Psi_5\rangle$	$\|\Psi_6\rangle$
−15/2	$-0.003740 + 0.006296i$	$-0.035279 + 0.059383i$
−13/2	$0.117195 + 0.081597i$	$0.254717 + 0.120795i$
−11/2	$0.216147 - 0.038900i$	$-0.132484 + 0.160942i$
−9/2	$0.142598 + 0.117510i$	$-0.071902 + 0.082600i$
−7/2	$-0.112675 + 0.064616i$	$0.139061 + 0.084269i$
−5/2	$0.020346 - 0.185647i$	$0.123740 + 0.068809i$
−3/2	$0.178226 + 0.120272i$	$-0.204377 - 0.397106i$
−1/2	$-0.567080 + 0.126816i$	$-0.152112 - 0.229691i$
+1/2	$-0.119781 - 0.248090i$	$-0.398664 + 0.422764i$
+3/2	$0.237018 + 0.378531i$	$0.012372 + 0.214656i$
+5/2	$-0.004043 + 0.141528i$	$0.169998 + 0.077327i$
+7/2	$-0.001423 - 0.162595i$	$0.113101 - 0.063867i$
+9/2	$0.107738 - 0.019628i$	$-0.028195 - 0.182614i$
+11/2	$-0.206033 + 0.031699i$	$-0.143841 + 0.165960i$
+13/2	$-0.026246 + 0.280684i$	$-0.010294 - 0.142432i$
+15/2	-0.069072	0.007323

Table S14 $|J, M_J\rangle$ decomposition of the fourth Kramers doublet of the Dy(III) $^6H_{15/2}$ manifold.

	KD4 ($E = 245.9\,\mathrm{cm}^{-1}$)	
M_J	$\|\Psi_7\rangle$	$\|\Psi_8\rangle$
−15/2	$-0.041850 - 0.009085i$	$-0.066874 - 0.014517i$
−13/2	$-0.075416 + 0.043071i$	$0.209177 + 0.136287i$
−11/2	$0.141232 - 0.026833i$	$-0.467152 + 0.020266i$
−9/2	$-0.042852 + 0.108022i$	$-0.287003 + 0.175960i$
−7/2	$-0.106381 + 0.093070i$	$-0.115993 + 0.114111i$
−5/2	$-0.008602 - 0.019981i$	$0.088177 - 0.030445i$
−3/2	$0.235320 + 0.133272i$	$-0.005593 - 0.386990i$
−1/2	$-0.367194 - 0.366985i$	$-0.093544 - 0.025406i$
+1/2	$-0.096805 + 0.004983i$	$0.436688 - 0.280735i$
+3/2	$0.087562 - 0.376996i$	$0.258236 - 0.080318i$
+5/2	$0.079712 + 0.048458i$	$0.012645 - 0.017701i$
+7/2	$0.089146 + 0.136121i$	$-0.084216 - 0.113519i$
+9/2	$-0.243143 - 0.232840i$	$0.018961 + 0.114654i$
+11/2	$0.452220 + 0.118907i$	$0.132325 + 0.056184i$
+13/2	$0.233328 - 0.088810i$	$0.064562 + 0.058089i$
+15/2	0.068432	-0.042825

Table S15 $|J, M_J\rangle$ decomposition of the fifth Kramers doublet of the Dy(III) $^6H_{15/2}$ manifold.

	KD5 ($E = 350.9\,\mathrm{cm}^{-1}$)	
M_J	$\lvert\Psi_9\rangle$	$\lvert\Psi_{10}\rangle$
−15/2	−0.004935 − 0.015099*i*	0.017371 + 0.053143*i*
−13/2	−0.251814 − 0.177723*i*	−0.080969 − 0.012662*i*
−11/2	0.385668 − 0.016374*i*	0.179189 + 0.032561*i*
−9/2	−0.118794 − 0.129888*i*	0.033811 + 0.038949*i*
−7/2	−0.026760 − 0.140704*i*	0.245725 − 0.131087*i*
−5/2	−0.195582 + 0.405796*i*	0.111429 + 0.348810*i*
−3/2	−0.142679 − 0.315959*i*	−0.003973 − 0.223343*i*
−1/2	−0.078531 − 0.145641*i*	−0.179912 + 0.119048*i*
+1/2	0.057258 − 0.207996*i*	0.162833 + 0.029393*i*
+3/2	0.213524 − 0.065616*i*	−0.344652 − 0.037450*i*
+5/2	0.366167 − 0.002460*i*	−0.324946 + 0.311983*i*
+7/2	0.048253 − 0.274292*i*	−0.142054 + 0.018281*i*
+9/2	0.047527 + 0.020036*i*	0.160369 + 0.072559*i*
+11/2	−0.086623 − 0.160204*i*	0.104263 + 0.371668*i*
+13/2	−0.037192 − 0.073028*i*	0.247165 + 0.184133*i*
+15/2	−0.055910	−0.015885

Table S16 $|J, M_J\rangle$ decomposition of the sixth Kramers doublet of the Dy(III) $^6H_{15/2}$ manifold.

	KD6 ($E = 430.8\,\mathrm{cm}^{-1}$)	
M_J	$\lvert\Psi_{11}\rangle$	$\lvert\Psi_{12}\rangle$
−15/2	0.002184 − 0.008153*i*	−0.000327 + 0.001220*i*
−13/2	−0.003608 − 0.013342*i*	−0.018757 + 0.055185*i*
−11/2	−0.424505 + 0.253148*i*	0.081048 + 0.092340*i*
−9/2	−0.296844 − 0.065197*i*	−0.144129 − 0.132044*i*
−7/2	−0.031107 − 0.214419*i*	0.173495 − 0.016298*i*
−5/2	−0.117051 + 0.399909*i*	0.203318 + 0.121489*i*
−3/2	0.022460 + 0.448534*i*	0.014247 + 0.018964*i*
−1/2	−0.173165 + 0.244763*i*	0.046561 − 0.070927*i*
+1/2	−0.080561 + 0.026620*i*	−0.281239 + 0.103922*i*
+3/2	−0.014631 − 0.018669*i*	0.427441 + 0.137773*i*
+5/2	0.064733 + 0.227832*i*	−0.416578 + 0.009570*i*
+7/2	0.060642 − 0.163367*i*	−0.199064 − 0.085537*i*
+9/2	−0.090246 − 0.173391*i*	−0.013845 + 0.303604*i*
+11/2	−0.068220 − 0.102184*i*	0.354383 − 0.344530*i*
+13/2	0.058159 − 0.003836*i*	0.011954 + 0.006938*i*
+15/2	−0.001263	−0.008441

Table S17 $|J, M_J\rangle$ decomposition of the seventh Kramers doublet of the Dy(III) $^6H_{15/2}$ manifold.

	KD7 ($E = 471.1\,\mathrm{cm}^{-1}$)	
M_J	$\lvert\Psi_{13}\rangle$	$\lvert\Psi_{14}\rangle$
$-15/2$	$-0.027132 + 0.005132i$	$-0.107056 + 0.020250i$
$-13/2$	$-0.041972 - 0.037415i$	$-0.131371 + 0.074393i$
$-11/2$	$0.144683 + 0.013828i$	$0.102197 - 0.079334i$
$-9/2$	$-0.255875 + 0.156537i$	$-0.148006 + 0.332715i$
$-7/2$	$0.000902 - 0.096746i$	$-0.056749 + 0.624275i$
$-5/2$	$-0.080348 + 0.147175i$	$-0.252517 + 0.206703i$
$-3/2$	$-0.020751 - 0.186703i$	$-0.139975 + 0.203452i$
$-1/2$	$-0.064627 + 0.225262i$	$0.081071 + 0.079340i$
$+1/2$	$0.064913 - 0.093025i$	$0.105367 + 0.209326i$
$+3/2$	$0.175349 + 0.173892i$	$0.014311 + 0.187307i$
$+5/2$	$-0.286534 - 0.156170i$	$0.106301 + 0.129677i$
$+7/2$	$0.171786 + 0.602851i$	$0.018867 + 0.094893i$
$+9/2$	$-0.207265 - 0.299411i$	$0.280510 + 0.106253i$
$+11/2$	$-0.115161 - 0.058958i$	$0.139592 - 0.040478i$
$+13/2$	$-0.142909 - 0.048681i$	$0.034287 - 0.044564i$
$+15/2$	0.108954	-0.027613

Table S18 $|J, M_J\rangle$ decomposition of the eighth Kramers doublet of the Dy(III) $^6H_{15/2}$ manifold.

	KD8 ($E = 508.7\,\mathrm{cm}^{-1}$)	
M_J	$\lvert\Psi_{15}\rangle$	$\lvert\Psi_{16}\rangle$
$-15/2$	$-0.018805 + 0.035838i$	$-0.025802 + 0.049173i$
$-13/2$	$0.018329 + 0.012249i$	$0.119356 - 0.023264i$
$-11/2$	$-0.005215 - 0.194394i$	$0.080322 - 0.207015i$
$-9/2$	$0.028627 + 0.180073i$	$-0.354480 + 0.491680i$
$-7/2$	$0.172138 + 0.023909i$	$0.243889 - 0.436837i$
$-5/2$	$-0.200562 - 0.014231i$	$-0.251022 - 0.172375i$
$-3/2$	$-0.088138 + 0.056945i$	$0.130214 + 0.052657i$
$-1/2$	$0.077109 - 0.172051i$	$0.109413 + 0.017575i$
$+1/2$	$0.035275 - 0.105051i$	$-0.188180 - 0.011662i$
$+3/2$	$-0.013875 + 0.139771i$	$-0.091377 + 0.051587i$
$+5/2$	$0.036002 + 0.302372i$	$0.080588 - 0.184209i$
$+7/2$	$-0.500139 + 0.012990i$	$0.058811 - 0.163537i$
$+9/2$	$-0.600089 + 0.085436i$	$0.146153 + 0.109019i$
$+11/2$	$-0.220632 - 0.025063i$	$0.169712 + 0.094942i$
$+13/2$	$0.076058 - 0.094880i$	$0.002330 + 0.021922i$
$+15/2$	0.055531	-0.040472

7 Supplementary Note 7: Dynamical accumulation of toroidal polarisation

The magnetoelectric tensor for which we developed a computational strategy in the in the previous section, can be used to evaluate the induced magnetic or electric moment that would arise via the magnetoelectric effect for e.g. one of the maximally toroidal ground states of the ring. The corresponding time-reversed toroidal state carries an induced electric or magnetic moment that exactly opposes its degenerate partner. Thus, the actual response measured in experiment depends on the population difference between time-reversed partners which must be induced by an external perturbation. In this supplementary note, we illustrate a mechanism for the accumulation of population in one of the two toroidal ground states (which give the leading order contributions to the toroidal polarisation) via ultra-fast laser driving.

7.1 Effective model for the accumulation of toroidal polarisation

To illustrate the potential for toroidal accumulation in a periodically driven system, we first consider an effective two-level model that highlights the crucial role of the asymmetric drive. The time-evolution of the reduced density operator for the two-level open quantum system is determined by the Lindblad equation

$$\dot{\rho} = -\frac{i}{\hbar}\left[\mathcal{H}(t), \rho\right] + \kappa g(t)\mathcal{L}[\sigma_+] + \kappa h(t)\mathcal{L}[\sigma_-] \tag{S67}$$

where $\mathcal{H}(t) = \boldsymbol{\tau} \cdot \frac{1}{c^2}\frac{\partial \mathbf{E}(t)}{\partial t}\sigma_z = \boldsymbol{\tau} \cdot (\nabla \times \mathbf{B}(t))\sigma_z$ and $\mathcal{L}[O] = O\rho O^\dagger - \frac{1}{2}O^\dagger O\rho - \frac{1}{2}\rho O^\dagger O$ are Lindblad jump operators that transfer population between the two states of the system. We assume time-dependent dissipative rates $g(t)$ and $h(t)$ that obey detailed balance at a given time t and depend on the level splitting induced by $\nabla \times \mathbf{B}(t)$. Assuming $\kappa \ll 1$ at low temperatures, we seek a series solution to Eq. (S67) of the form $\rho(t) = \sum_{r=0}^{\infty} \kappa^r \rho^{(r)}(t)$ where $\rho^{(r)}(t)$ are to be determined. Substituting this ansatz into Eq. (S67) and collecting powers of κ, leads to the following set of coupled differential equations

$$\begin{aligned}
\dot{\rho}^{(0)} &= -\frac{i}{\hbar}\left[\mathcal{H}(t), \rho^{(0)}\right] \\
\dot{\rho}^{(1)} &= -\frac{i}{\hbar}\left[\mathcal{H}(t), \rho^{(1)}\right] + g(t)\mathcal{L}^{(0)}[\sigma_+] + h(t)\mathcal{L}^{(0)}[\sigma_-] \\
&\vdots \\
\dot{\rho}^{(r+1)} &= -\frac{i}{\hbar}\left[\mathcal{H}(t), \rho^{(r+1)}\right] + g(t)\mathcal{L}^{(r)}[\sigma_+] + h(t)\mathcal{L}^{(r)}[\sigma_-] \\
&\vdots
\end{aligned} \tag{S68}$$

where we employ the notation $\mathcal{L}^{(r)}[O] = O\rho^{(r)}O^\dagger - \frac{1}{2}O^\dagger O\rho^{(r)} - \frac{1}{2}\rho^{(r)}O^\dagger O$. Solution of the zeroth order term yields

$$\rho^{(0)}(t) = \begin{pmatrix} \rho_{11}^{(0)}(0) & \rho_{21}^{(0)}(0)e^{\frac{-i}{\hbar}\boldsymbol{\tau}\cdot\int_0^t dt'\nabla\times\mathbf{B}(t')} \\ \rho_{12}^{(0)}(0)e^{\frac{i}{\hbar}\boldsymbol{\tau}\cdot\int_0^t dt'\nabla\times\mathbf{B}(t')} & \rho_{22}^{(0)}(0) \end{pmatrix} \tag{S69}$$

where clearly in the absence of dissipation, only a dynamical phase is accumulated on the coherences. We will always consider the system to be initialised in a maximally mixed state $\rho(0) = \frac{1}{2}\mathbb{I}$ (with $\mathbb{I}$ the 2×2 identity matrix) and, accordingly, since $\rho(0) = \rho^{(0)}(0) + \kappa\rho^{(1)}(0) + \ldots$, we fix $\rho^{(0)}(0) = \frac{1}{2}\mathbb{I}$ and $\rho^{(r\neq 0)}(0) = 0$. Noting the form of $\rho^{(0)}(t) = \frac{1}{2}\mathbb{I}$, it is straightforward to evaluate the Lindblad operators $\mathcal{L}^{(0)}[\sigma_+] = \frac{1}{2}\sigma_z$ and $\mathcal{L}^{(0)}[\sigma_-] = -\frac{1}{2}\sigma_z$. Resulting in the following inhomogeneous differential equation for the linear correction $\rho^{(1)}$

$$\begin{aligned} \dot{\rho}^{(1)} &= -\frac{i}{\hbar}\left[\mathcal{H}(t), \rho^{(1)}\right] + \frac{1}{2}\left(g(t) - h(t)\right)\sigma_z \\ \implies \rho^{(1)}(t) &= \frac{1}{2}\sigma_z \int_0^t dt' \left(g(t') - h(t')\right) \end{aligned} \tag{S70}$$

that, importantly, is found to be linear in σ_z. For $r \geq 2$, we now make the inductive hypothesis

$$\rho^{(r)}(t) = -\frac{(-1)^r}{2}\sigma_z \int_0^t dt_1 \cdots \int_0^{t_{r-1}} dt_r \left(\prod_{j=1}^{r-1} g(t_j) + h(t_j)\right)\left(g(t_r) - h(t_r)\right). \tag{S71}$$

Substituting Eq. (S71) into the equation of motion for $\rho^{(r+1)}$ shown in Eq. (S68) yields

$$\begin{aligned} \dot{\rho}^{(r+1)} &= -\frac{i}{\hbar}\left[\mathcal{H}(t), \rho^{(r+1)}\right] - \frac{(-1)^r}{2}\sigma_z\left(g(t) + h(t)\right) \\ &\times \int_0^t dt_1 \cdots \int_0^{t_{r-1}} dt_r \left(\prod_{j=1}^{r-1} g(t_j) + h(t_j)\right)\left(g(t_r) - h(t_r)\right). \end{aligned} \tag{S72}$$

The solution of Eq. (S72) gives

$$\rho^{(r+1)}(t) = -\frac{(-1)^{r+1}}{2}\sigma_z \int_0^t dt_1 \cdots \int_0^{t_r} dt_{r+1} \left(\prod_{j=1}^{r} g(t_j) + h(t_j)\right)\left(g(t_{r+1}) - h(t_{r+1})\right) \tag{S73}$$

validating the inductive hypothesis. We finally have that the time evolution of the toroidal polarisation accumulated in the two-level system is

$$\begin{aligned}\langle\tau\rangle &= |\boldsymbol{\tau}|\,\mathrm{Tr}\left\{\sigma_z\rho\right\}\\ &= \kappa|\boldsymbol{\tau}|\sum_{r=1}^{\infty}(-\kappa)^{r-1}\int_0^t dt_1\cdots\int_0^{t_{r-1}} dt_r\left(\prod_{j=1}^{r-1} g(t_j)+h(t_j)\right)(g(t_r)-h(t_r))\end{aligned} \tag{S74}$$

where the case $r=1$ in the summand should be understood to reproduce a term similar to Eq. (S70).

We consider an electric field $\mathbf{E}(t)$ that varies periodically in time with period T_l. The field in a time period $nT_l \leq t \leq (n+1)T_l$ with $n \in 0 \cup \mathbb{N}$ is described by

$$\mathbf{E}(t) = \begin{cases} \frac{\epsilon E_0 t}{T_l}\hat{\mathbf{z}} & nT_l \leq t < (n+1/\epsilon)T_l \\ \frac{\epsilon E_0(T_l-2t)}{T_l(\epsilon-2)}\hat{\mathbf{z}} & (n+1/\epsilon)T_l \leq t < (n+1-1/\epsilon)T_l \\ \frac{\epsilon E_0(t-T_l)}{T_l}\hat{\mathbf{z}} & (n+1-1/\epsilon)T_l \leq t < (n+1)T_l \end{cases} \tag{S75}$$

which generates the quasi-static time-dependent curl

$$\frac{1}{c^2}\frac{\partial\mathbf{E}(t)}{\partial t} = \nabla\times\mathbf{B}(t) = \begin{cases} \frac{\epsilon E_0}{c^2 T_l}\hat{\mathbf{z}} & nT_l \leq t < (n+1/\epsilon)T_l \\ \frac{-2\epsilon E_0}{c^2 T_l(\epsilon-2)}\hat{\mathbf{z}} & (n+1/\epsilon)T_l \leq t < (n+1-1/\epsilon)T_l \\ \frac{\epsilon E_0}{c^2 T_l}\hat{\mathbf{z}} & (n+1-1/\epsilon)T_l \leq t < (n+1)T_l \end{cases} \tag{S76}$$

that drives the two-level system. Note that integrating $\mathbf{E}(t)$ over a period T_l gives a net zero electric field on average and that the parameter ϵ controls the degree of asymmetry in the electric field carrier shape. For illustration, we take $g(t)$ and $h(t)$ to be effective Debye-like direct relaxation between the two states

$$g(t) = \frac{\Delta(t)^3}{e^{\beta\Delta(t)}-1} = e^{-\beta\Delta(t)}h(t) \tag{S77}$$

where $\Delta(t) = 2\boldsymbol{\tau}\cdot(\nabla\times\mathbf{B}(t))$ is the time-dependent toroidal splitting induced by the curling field in Eq. (S76). We will relax this assumption in the following subsection in favour of a multi-step spin-phonon and quantum tunnelling model of dissipation. As a consequence of detailed balance, it is useful to note that $g(t)-h(t) = -\Delta(t)^3$ and $g(t)+h(t) = \Delta(t)^3/\tanh(\beta\Delta(t)/2)$. For these functions, Eq. (S74) takes the form

$$\langle\tau\rangle = \kappa|\boldsymbol{\tau}|\sum_{r=1}^{\infty}(-\kappa)^{r-1}\int_0^t dt_1\cdots\int_0^{t_{r-1}} dt_r\left(\prod_{j=1}^{r-1}\frac{\Delta(t_j)^3}{\tanh(\beta\Delta(t_j)/2)}\right)\Delta(t_r)^3 \tag{S78}$$

which is straightforwardly evaluated via piecewise partitioning of the integrals onto domains in which $\nabla\times\mathbf{B}(t)$ is constant.

It is instructive to compute the linear accumulation of toroidal polarisation over $t = N_{\text{cycles}} T_l$. Using Eq. (S74) truncated to linear order, we have

$$
\begin{aligned}
\langle \tau(t) \rangle &= \kappa |\boldsymbol{\tau}| \int_0^t dt_1 \Delta(t)^3 + \mathcal{O}(\kappa^2) \\
&= \sum_{j=1}^{N_{\text{cycles}}} \frac{8\kappa |\boldsymbol{\tau}|^4 \epsilon^3}{T_l^3} \left(\frac{E_0}{c^2} \right)^3 \left(\int_0^{T_l/\epsilon} dt_1 - \left(\frac{2}{\epsilon - 2} \right)^3 \int_{T_l/\epsilon}^{T_l(1-1/\epsilon)} dt_1 + \int_{T_l(1-1/\epsilon)}^{T_l} dt_1 \right) + \mathcal{O}(\kappa^2) \\
&= \frac{16\kappa |\boldsymbol{\tau}|^4}{T_l^3} \left(\frac{E_0}{c^2} \right)^3 \frac{\epsilon^3(\epsilon - 4)}{(\epsilon - 2)^2} t + \mathcal{O}(\kappa^2).
\end{aligned}
\tag{S79}
$$

From Eq. (S79), it is clear that to maximise toroidal accumulation, the asymmetry of the carrier ϵ should also be maximised (with accumulation vanishing in the symmetric limit $\epsilon = 4$), with a large field amplitude E_0 being delivered over a short period of time T_l. It is also noteworthy that the accumulated toroidal polarisation is quartic in $|\boldsymbol{\tau}|$, providing further motivation to seek ring systems with large toroidal moments in the ground state. To second order in κ, the accumulation in the interval $0 \leq t \leq T_l$ is

$$
\begin{aligned}
\langle \tau \rangle &= \frac{16\kappa |\boldsymbol{\tau}|^4 \epsilon^3 E_0^3}{c^6 T_l^2} \frac{\epsilon - 4}{(\epsilon - 2)^2} \\
&\times \left(1 - \frac{8\kappa |\boldsymbol{\tau}|^3 \epsilon^2 E_0^3}{c^6 T_l^2} \left(4 \coth \frac{2\beta\epsilon |\boldsymbol{\tau}| E_0}{c^2 T_l (\epsilon - 2)} + (\epsilon - 2)^2 \coth \frac{\beta \epsilon |\boldsymbol{\tau}| E_0}{c^2 T_l} \right) \right) + \mathcal{O}(\kappa^3)
\end{aligned}
\tag{S80}
$$

where thermal effects serve to reduce toroidal polarisation.

7.2 Dynamical simulations of toroidal polarisation in $Fe_{10}Dy_{10}$

While the above model demonstrates the physical motivation for an asymmetric electric field carrier shape, $Fe_{10}Dy_{10}$ is far from a simple two-level system and direct population transfer between its maximally toroidal ground states is, in general, highly suppressed by requiring multiple simultaneous Dy^{III} flips. At the same time, a full simulation of $Fe_{10}Dy_{10}$ is computationally prohibited by its enormous Hilbert space. Therefore, to develop a faithful but computationally tractable dynamical simulation of toroidal polarisation in $Fe_{10}Dy_{10}$, we make two physically motivated simplifications:

1. We consider a dynamics in the Ising degrees of freedom only i.e. the Fe^{III} spin states are "frozen" in their zero-curling field ground configurations, a reasonable assumption for weak driving,
2. We reduce the remaining 1024 dimensional configuration basis of Dy^{III} magnetic moment orientations to a smaller basis defined by all states with energy ≤ 2.5 cm^{-1} which is justified for low temperatures ($T \leq 3.5$ K) and weak driving.

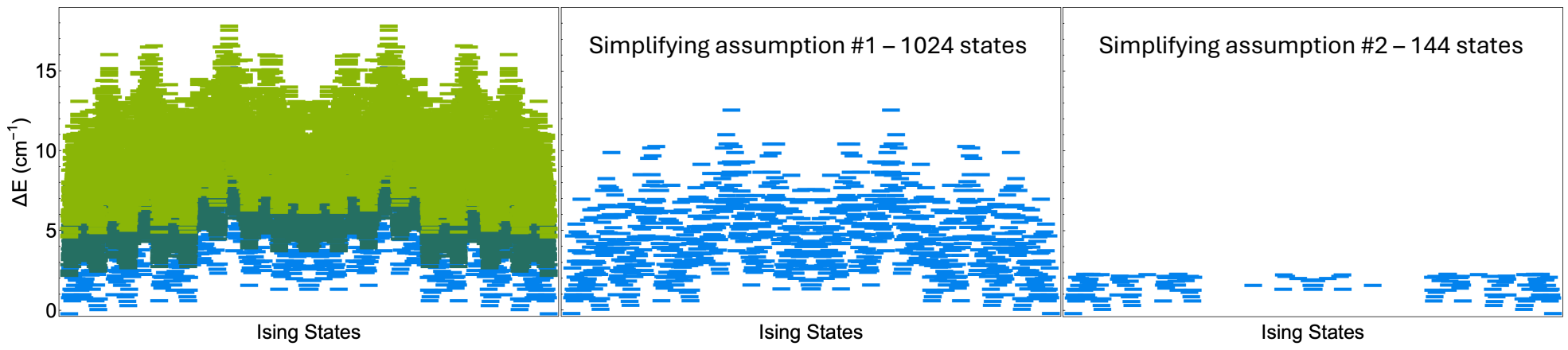

Fig. S5 Comparative representation of the $Fe_{10}Dy_{10}$ basis after the simplifying assumptions taken above with zero applied magnetic field. Source data are provided as a Source Data file.

The effect of these simplifying assumptions on the size of the basis is shown in Figure S5.

The non-equilibrium time-evolution of $Fe_{10}Dy_{10}$ populations in this reduced Hilbert space is governed by the master equations [4, 5, 12–14]

$$\frac{d\rho_m}{dt} = \sum_{k\neq m}\left(W^{k\to m}(t) + \Omega^{k\leftrightarrow m}(t)\right)\rho_k - \rho_m \sum_{k\neq m}\left(W^{m\to k}(t) + \Omega^{k\leftrightarrow m}(t)\right) \tag{S81}$$

where ρ_m are the diagonal matrix elements of the reduced density matrix represented on the restricted Ising basis shown in Figure S5. Population is transferred between states by spin-phonon transitions

$$W^{m\to k}(t) = \left(\Gamma_1\delta_{mk}^{1-\mathrm{Dy}} + \Gamma_2\delta_{mk}^{2-\mathrm{Dy}} + \Gamma_3\delta_{mk}^{3-\mathrm{Dy}}\right) \times \frac{\left(E_k(t) - E_m(t)\right)^3}{e^{\beta(E_k(t)-E_m(t))} - 1} \tag{S82}$$

and quantum tunnelling

$$\Omega^{m\leftrightarrow k}(t) = \left(\gamma_1\delta_{mk}^{1-\mathrm{Dy}} + \gamma_2\delta_{mk}^{2-\mathrm{Dy}} + \gamma_3\delta_{mk}^{3-\mathrm{Dy}}\right) \times \frac{\xi}{\left((E_k(t) - E_m(t))/\hbar\right)^2 + \xi^2}. \tag{S83}$$

In both Eq. S82 and Eq. S83 we have made use of the symbol $\delta_{mk}^{r-\mathrm{Dy}}$ which is equal to 1 if the Ising configurations m and k can be connected by r Dy magnetic moment flips, and is equal to 0 otherwise. Furthermore, $E_k(t)$ is the semi-classical energy of Dy^{III} configuration k under time-dependent driving of a curling magnetic field $(\nabla\times\mathbf{B})(t)$ and $\beta = 1/k_BT$ is the inverse temperature. For our simulations we adopt the leading-order spin-phonon coupling constant $\Gamma_1 = 10^2$ cm^3 s^{-1}, and the quantum tunnelling parameters $\gamma_1 = 10^{16}$ s^{-2} and $\xi = 10^{10}$ s^{-1}, corresponding to an on-resonance tunnelling rate $\gamma_1/\xi \sim 10^6$ s^{-1} [15], compatible with tunnelling scales reported for other dysprosium single-molecule magnets [4, 5, 16]. We further imposed a reasonable hierarchy on the higher-order multi-flip spin-phonon rate constants, $\Gamma_2 = 10^{-2}\Gamma_1$ and $\Gamma_3 = 10^{-4}\Gamma_1$. A similar hierarchy was adopted for the multi-site quantum tunnelling rate constants, $\gamma_2 = 10^{-1}\gamma_1$ and $\gamma_3 = 10^{-2}\gamma_1$, which conservatively represent the fastest identified two-site and three-site tunnelling processes in $Fe_{10}Dy_{10}$. The relative efficiency of such processes was estimated from products of the transverse ab initio g-tensor components reported in Supplementary Tables S1–S5, whose strong variability reflects the broad distribution of local transverse anisotropies and tunnelling efficiencies in this system.

As laid out in the main text and previous subsection, a time-dependent curling magnetic field arises from the temporal variation of an electric field $\mathbf{E}(t)$ according to Maxwell's equations. With a sufficiently temporally-asymmetric electric field ramp, a time-evolving curl will be generated that biases population transfer to just one Ising state of each time- and parity-related doublet (states that carry equal but opposing toroidal moments), thus generating a toroidal polarisation. A realistic set-up for temporally-asymmetric electric field ramp generation was recently proposed and involved the first few harmonics of a 1600 nm near infrared laser [17]. For a general asymmetry parameter ϵ, the Fourier expansion required to approximate the asymmetric sawtooth shape in Eq. (S75) is

$$\mathbf{E}(t) = \frac{\hat{\mathbf{z}}}{\pi^2}\frac{\epsilon}{\epsilon-2}\sum_{k=1}^{\infty}\frac{(-1)^{k+1}}{k^2}\sin\left(\frac{k\pi\,(\epsilon-2)}{\epsilon}\right)\sin\left(\frac{2\pi kct}{\lambda}\right). \tag{S84}$$

We show in Figure S6 that the first 3 excited harmonics corresponding to 800 nm, 533 nm and 400 nm (achievable via optical parametric amplification [17]) already give a faithful approximation to this lineshape (orange dashed). We consider an electric field amplitude $E_0 = 10^7$ V m^{-1} that gives an asymmetric curling magnetic field which varies asymmetrically between -0.7×10^{-4} T nm^{-1} $\leq (\nabla\times\mathbf{B})_z \leq 2.5\times10^{-4}$ T nm^{-1}. We consider pulse envelopes of 107 fs corresponding to $N_{\text{cycles}} = 20$ cycles of the electric field.

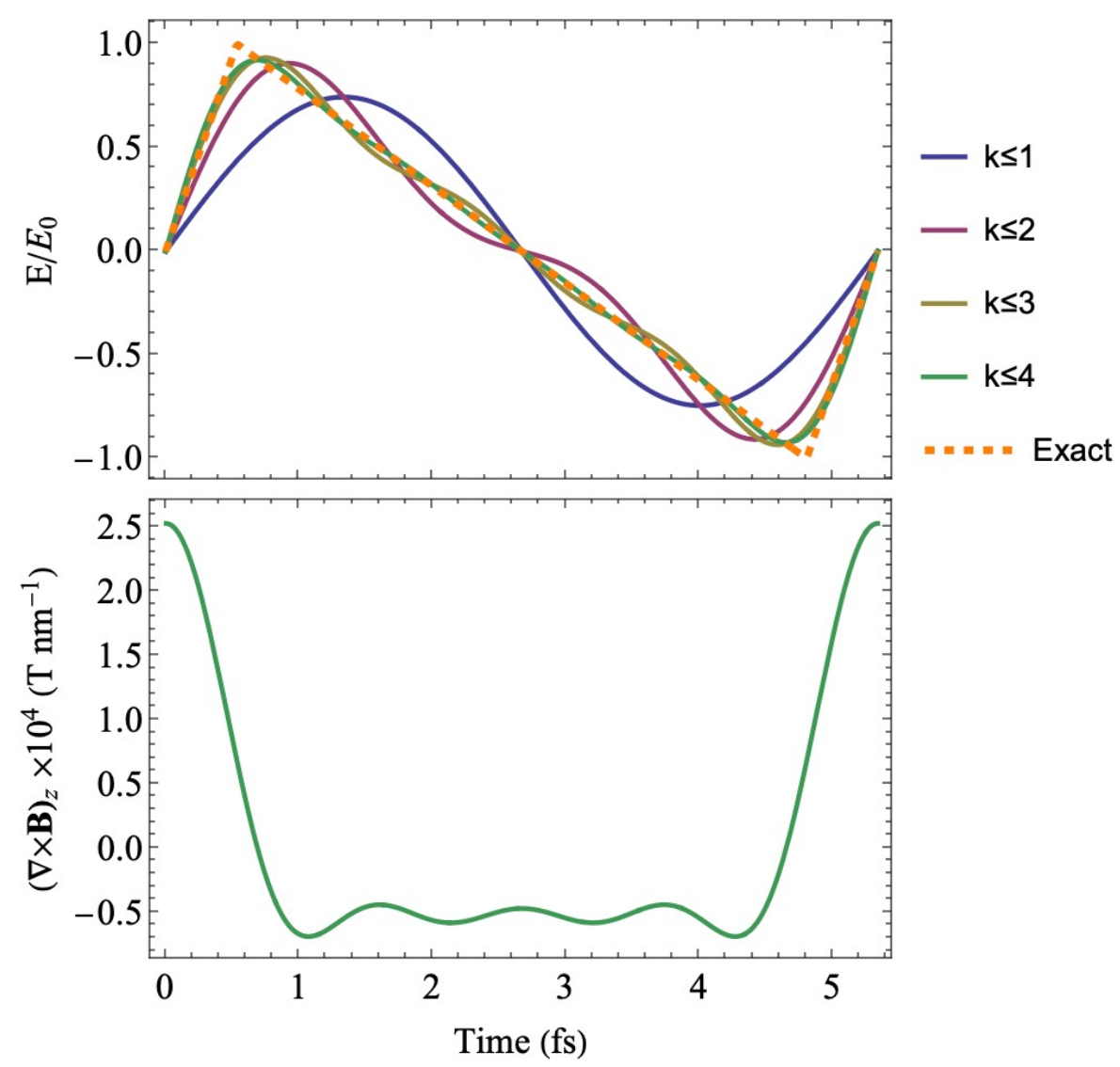

Fig. S6 (Top) Fourier series approximations over one period $T_l = \lambda/c$ to the exact (dashed orange) asymmetric sawtooth in Eq. (S75) with $\epsilon = 10$. (Bottom) Curling magnetic field generated with $k \leq 4$ and $E_0 = 10^7$ V m^{-1}. Source data are provided as a Source Data file.

To quantify the degree of toroidal polarisation in a single molecule after one pulse of radiation, we numerically integrated Eq. (S81) for $N_{\text{cycles}} = 20$ with the time-dependent rates $W^{k\to m}(t)$ and $\Omega^{k\leftrightarrow m}(t)$ modulated by the time-evolving curl $\frac{1}{c^2}\frac{\partial \mathbf{E}(t)}{\partial t}|_z = (\nabla \times \mathbf{B})|_z$ shown in Figure S6.

The number of $Fe_{10}Dy_{10}$ molecules within the laser-irradiated volume is

$$N = \frac{\rho_{\text{Fe}_{10}\text{Dy}_{10}} N_A V_{\text{laser}}}{M_{\text{Fe}_{10}\text{Dy}_{10}}} \approx 4.75 \times 10^{13}. \tag{S85}$$

A microSQUID moment sensitivity of $\sim 10^{-16}$ emu corresponds to $\sim 10^{-19}$ A m^2, or approximately $\sim 10^4\,\mu_B$ [18, 19], which is the detectability threshold used in Figure 6c of the main text.

7.2.1 Laser heating and pulse-train thermal accumulation

We now estimate the temperature increase induced by laser heating. The low-temperature heat capacity extracted from Figure 4 of the main text is approximately $C/k_B \sim 3$ for $T \lesssim 1$ K. The temperature increase $\Delta T = \Delta Q_{\text{abs}}/(mC_s)$ depends on the irradiated sample mass $m = \rho_{\text{Fe}_{10}\text{Dy}_{10}} V_{\text{laser}} = \pi \times 1.716 \times 10^{-7}$ g, where the density of $Fe_{10}Dy_{10}$ is $\rho_{\text{Fe}_{10}\text{Dy}_{10}} = 1.716 \times 10^6$ g m^{-3} and the irradiated volume is $V_{\text{laser}} = \pi \times 10^{-13}$ m^3, corresponding to a sample depth $L_{\text{depth}} = 1$ mm $= 0.1$ cm and a laser spot diameter of 20 μm. The specific heat is estimated as

$$C_s = \frac{C N_A}{M_{\text{Fe}_{10}\text{Dy}_{10}}} \simeq 3.64 \times 10^{-3}\ \text{J}\,\text{g}^{-1}\,\text{K}^{-1},$$

where $M_{\text{Fe}_{10}\text{Dy}_{10}} = 6838.6$ g mol^{-1} is the molecular weight and $N_A = 6.02 \times 10^{23}$ mol^{-1} is Avogadro's number.

For an electric field amplitude $E_0 = 10^7$ V m^{-1}, the cycle-averaged laser intensity is

$$I = \frac{1}{2} c\epsilon_0 E_0^2 \simeq 1.33 \times 10^{11}\ \text{W}\,\text{m}^{-2},$$

corresponding to a fluence of approximately 1.42 μJ cm^{-2} for a pulse duration

$$t_{\text{pulse}} = \frac{N_{\text{cycles}}\lambda}{c} \approx 107\ \text{fs}.$$

Unlike resonant optical absorption experiments, however, the present protocol is intended to operate in a quasi-transparent non-resonant regime, where the time-dependent electric field acts primarily through the induced curling magnetic field $\nabla \times \mathbf{B}(t)$ rather than coupling to transition dipole moments and inducing direct electronic absorption. We therefore estimate the absorbed fraction using a Beer–Lambert attenuation factor

$$f_{\text{abs}} = 1 - \exp(-\varepsilon C L),$$

with a representative weak-absorption coefficient $\varepsilon = 1\ \mathrm{M}^{-1}\mathrm{cm}^{-1}$, sample thickness $L = 0.1$ cm, and molecular concentration

$$C = \frac{\rho_{\mathrm{Fe_{10}Dy_{10}}}}{M_{\mathrm{Fe_{10}Dy_{10}}}} \simeq 0.25\ \mathrm{M}.$$

This gives an absorbance $\varepsilon CL \simeq 0.025$ and therefore an absorbed fraction

$$f_{\mathrm{abs}} \simeq 2.5 \times 10^{-2}.$$

The incident pulse energy is then

$$\Delta Q_{\mathrm{inc}} = IAt_{\mathrm{pulse}} \simeq 4.46 \times 10^{-12}\ \mathrm{J},$$

with $A = \pi \times 10^{-10}\ \mathrm{m}^2$ the laser spot area, while the absorbed heat per pulse becomes

$$\Delta Q_{\mathrm{abs}} = f_{\mathrm{abs}} \Delta Q_{\mathrm{inc}} \simeq 1.11 \times 10^{-13}\ \mathrm{J}.$$

This yields a temperature increase

$$\Delta T_0 = \frac{\Delta Q_{\mathrm{abs}}}{mC_s} \simeq 5.7 \times 10^{-5}\ \mathrm{K} = 0.057\ \mathrm{mK} \tag{S86}$$

per pulse.

The characteristic time required for $1/e$ of the accumulated heat to diffuse from the irradiated spot is estimated as

$$t_{\mathrm{diff}} \approx \frac{L^2}{\alpha},$$

where $L = 10\ \mu$m is the spot radius and

$$\alpha = \frac{\kappa_{\mathrm{therm}}}{\rho_{\mathrm{Fe_{10}Dy_{10}}} C_s}$$

is the thermal diffusivity. The thermal conductivity κ_{therm} of $Fe_{10}Dy_{10}$ is currently unknown; we estimate a conservative low-temperature value $\kappa_{\mathrm{therm}} = 1\ \mathrm{W\ m^{-1}\ K^{-1}}$ based on previously reported molecular systems [20–22], yielding a diffusion time

$$t_{\mathrm{diff}} \approx 630\ \mathrm{ns}.$$

To estimate the thermal accumulation induced by repeated ultrafast pulses, we adopt the standard linear superposition framework commonly used in high-repetition-rate laser-heating problems [23, 24]. Following the classical heat-diffusion treatment for an initially Gaussian temperature profile [25], the temperature increase generated

by a single absorbed pulse may be written as

$$\Delta T(r,t) = \Delta T_0 \left(\frac{R^2}{R^2 + \alpha_{\text{eff}} t} \right)^{3/2} \exp\left[-\frac{r^2}{R^2 + \alpha_{\text{eff}} t} \right], \tag{S87}$$

where R is the characteristic laser-spot radius and α_{eff} is an effective thermal diffusivity absorbing geometry-dependent numerical factors associated with the approximate three-dimensional diffusion model.

At the centre of the irradiated spot ($r = 0$), this gives

$$\Delta T_{\text{peak}}(t) = \Delta T_0 \left(1 + \frac{t}{t_{\text{diff}}} \right)^{-3/2}, \tag{S88}$$

with

$$t_{\text{diff}} = \frac{R^2}{\alpha_{\text{eff}}}. \tag{S89}$$

For a train of N pulses separated by a waiting time t_{wait}, linearity of the heat equation allows the residual thermal contribution from all previous pulses to be summed:

$$\Delta T(N) = \Delta T_0 \sum_{k=0}^{N-1} \left(1 + k \frac{t_{\text{wait}}}{t_{\text{diff}}} \right)^{-3/2}. \tag{S90}$$

In the experimentally relevant regime considered here,

$$t_{\text{wait}} \ll t_{\text{diff}},$$

the temperature decay changes only weakly between consecutive pulses, so that the discrete sum becomes well approximated by the corresponding continuous-limit integral. Defining

$$\epsilon = \frac{t_{\text{wait}}}{t_{\text{diff}}},$$

and substituting it into Eq. (S90) yields

$$\Delta T(N) \approx \Delta T_0 \int_0^N (1 + \epsilon x)^{-3/2} \, dx. \tag{S91}$$

Using the substitution

$$u = 1 + \epsilon x, \qquad du = \epsilon \, dx,$$

the integral becomes

$$\int_0^N (1 + \epsilon x)^{-3/2} \, dx = \frac{1}{\epsilon} \int_1^{1+\epsilon N} u^{-3/2} \, du = \frac{2}{\epsilon} \left[1 - \frac{1}{\sqrt{1 + \epsilon N}} \right]. \tag{S92}$$

The accumulated temperature increase therefore becomes

$$\Delta T(N) = \Delta T_0 \frac{2t_{\mathrm{diff}}}{t_{\mathrm{wait}}} \left[1 - \frac{1}{\sqrt{1 + N t_{\mathrm{wait}}/t_{\mathrm{diff}}}} \right]. \tag{S93}$$

Equation (S93) provides the thermal accumulation model used for the calculations reported in Figure 6d of the main text. For the pulse numbers required to reach the magnetoelectric detectability threshold, this diffusion-based model yields temperature increases very close to those obtained from a simple exponential relaxation estimate, while providing a more physically appropriate long-time description of heat spreading from the irradiated region.

We stress that Eq. (S93) should be regarded as a local peak-temperature estimate within an approximate three-dimensional diffusion model. Alternative sample geometries, thermal boundary conditions, or interface-limited heat transport may modify the long-time saturation behaviour, but have comparatively little effect on the short pulse-train regime relevant to the detectable toroidal accumulation predicted here.

8 χ^2 analysis of the exchange coupling parameters J_A, J_B and J_C

To assess the robustness of our broken symmetry DFT calculated exchange coupling constants (J_A^i, J_B^i and $J_C^{i,i+1}$) we also considered a non-collinear transfer matrix model of $Fe_{10}Dy_{10}$ magnetic response with J_A^{fit}, J_B^{fit} and J_C^{fit} as three fitting parameters that generically accounted for all Dy_i-Fe_i, Fe_i-Dy_{i+1} and Fe_i-Fe_{i+1} around the ring, respectively. We fit J_A^{fit}, J_B^{fit} and J_C^{fit} by performing a chi-squared minimization [26] of the experimental magnetic susceptibility data across parameter grids $J_A^{\text{fit}} \in [-5, 5]$ cm^{-1}, $J_B^{\text{fit}} \in [-5, 5]$ cm^{-1} and $J_C^{\text{fit}} \in [-1, 1]$ cm^{-1} (subsequent refinements of the search region were made close to the optimal point to give higher resolution).

We calculated the χ^2 value

$$\chi^2 = \sum_{i=1}^{N_{\text{data}}} \left(\frac{\chi_m T_i^{\text{exp}} - \chi_m T_i^{\text{sim}}(J_A^{\text{fit}}, J_B^{\text{fit}}, J_C^{\text{fit}})}{\sigma_i} \right)^2 \tag{S94}$$

as the sum of squared differences between the experimentally determined $\chi_m T^{\text{exp}}$ values and those simulated by the transfer matrix model for a given parameter set $\chi_m T^{\text{sim}}(J_A^{\text{fit}}, J_B^{\text{fit}}, J_C^{\text{fit}})$ (with experimental uncertainty σ_i taken here to be 1% of the experimental value). Dividing by the total number of degrees of freedom $\kappa = N_{\text{data}} - 3$ (where N_{data} is the number of experimental data points) defines the reduced chi-squared value $\tilde{\chi}^2$. The optimal parameter set that minimised the value of $\tilde{\chi}$ was $J_A^{\text{fit}} = 0.8^{0.2}_{-0.55}$ cm^{-1}, $J_B^{\text{fit}} = 0.6^{0.7}_{-0.15}$ cm^{-1} and $J_C^{\text{fit}} = -0.1^{0.1}_{-0.05}$ cm^{-1} which gave $\tilde{\chi}^2_{\text{min}} = 3.32$. We estimated the 68% confidence interval for each exchange coupling constant by the minimum and maximum values of each parameter in the parameter subset bounded by the contour $\tilde{\chi}^2 = \tilde{\chi}^2_{\text{min}} + \frac{\Delta^*}{\kappa}$ where $\Delta^* = 3.51$ for 3 parameters and an error tolerance of 68% [27].

In Figure S7 we plot $\tilde{\chi}^2$ as a function of the fitting parameters J_A^{fit} and J_B^{fit} with J_C^{fit} fixed as the mean value of the bs-DFT-derived exchange couplings (which lies within the J_C^{fit} confidence interval from our optimisation). There we show that the average exchange values from the bs-DFT calculations (green point) lie on the contour that defines the 68% confidence interval of our parameter optimisation. This independent analysis strongly demonstrates that the bs-DFT-derived exchange coupling constants accurately capture the non-dipolar intramolecular exchange interactions, and are thus quantitatively consistent in sign and magnitude for the $Fe_{10}Dy_{10}$ ring.

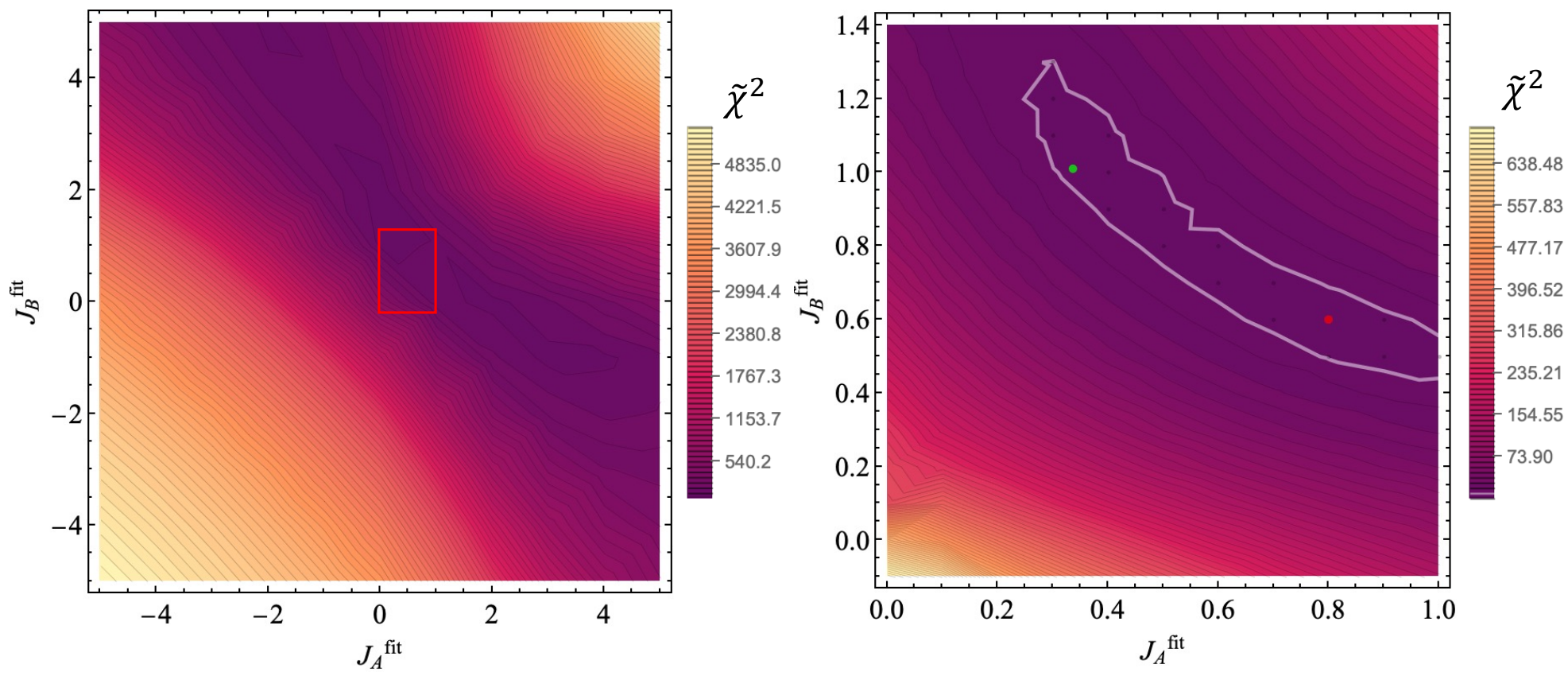

Fig. S7 Reduced chi-squared as a function of the exchange parameters J_A^{fit} and J_B^{fit} with J_C^{fit} fixed to the mean value of J_C from the bs-DFT calculations. (Left) $\tilde{\chi}^2$ during a wide parameter sweep of J_A^{fit} and J_B^{fit} with the red boxed region enclosing the smallest values of $\tilde{\chi}^2$. (Right) Parameter sweep of J_A^{fit} and J_B^{fit} in the red boxed region of the left panel. The red point indicates the parameters which minimise $\tilde{\chi}^2$ with the white contour indicating the 68% confidence interval of the parameters J_A^{fit} and J_B^{fit}. Sampled data points are shown as translucent black points inside this confidence region. The green point indicates a parameter choice of J_A^{fit}, J_B^{fit} and J_C^{fit} that coincides with the mean values of the bs-DFT derived exchange coupling constants. Source data are provided as a Source Data file.

Supplementary References

[1] Van den Heuvel, W., Calvello, S. & Soncini, A. Configuration-averaged 4f orbitals in ab initio calculations of low-lying crystal field levels in lanthanide(III) complexes. *Physical Chemistry Chemical Physics* **18**, 15807–15814 (2016).

[2] Calvello, S., Piccardo, M., Rao, S. V. & Soncini, A. CERES: An ab initio code dedicated to the calculation of the electronic structure and magnetic properties of lanthanide complexes. *Journal of Computational Chemistry* **39**, 328–337 (2018).

[3] Baniodeh, A. *et al.* Unraveling the influence of lanthanide ions on intra- and intermolecular electronic processes in $Fe_{10}Ln_{10}$ nano-toruses. *Advanced Functional Materials* **24**, 6280–6290 (2014).

[4] Vignesh, K. R. *et al.* Ferrotoroidic ground state in a heterometallic $\{Cr^{III}Dy_6^{III}\}$ complex displaying slow magnetic relaxation. *Nature Communications* **8**, 1–12 (2017).

[5] Ashtree, J. M. *et al.* Tuning the ferrotoroidic coupling and magnetic hysteresis in double-triangle complexes $\{Dy_3M^{III}Dy_3\}$ via the M^{III}-linker. *European Journal of Inorganic Chemistry* **2021**, 435–444 (2021).

[6] Spaldin, N. A., Fiebig, M. & Mostovoy, M. The toroidal moment in condensed-matter physics and its relation to the magnetoelectric effect. *Journal of Physics: Condensed Matter* **20**, 434203 (2008).

[7] Popov, A., Plokhov, D. & Zvezdin, A. Anapole moment and spin-electric interactions in rare-earth nanoclusters. *Europhysics Letters* **87**, 67004 (2009).

[8] Chilton, N. F., Collison, D., McInnes, E. J., Winpenny, R. E. & Soncini, A. An electrostatic model for the determination of magnetic anisotropy in dysprosium complexes. *Nature communications* **4**, 2551 (2013).

[9] Varshalovich, D. A., Moskalev, A. N. & Khersonskii, V. K. *Quantum Theory of Angular Momentum* (World Scientific, Singapore, 1988).

[10] Chibotaru, L. F. & Ungur, L. Ab initio calculation of anisotropic magnetic properties of complexes. i. unique definition of pseudospin hamiltonians and their derivation. *The Journal of chemical physics* **137** (2012).

[11] Wybourne, B. G. & Smentek, L. *Optical Spectroscopy of Lanthanides: Magnetic and Hyperfine Interactions* (CRC Press, Boca Raton, 2007).

[12] Yu, K.-X. *et al.* Enhancing magnetic hysteresis in single-molecule magnets by ligand functionalization. *Chem* **6**, 1777–1793 (2020).

[13] Hymas, K. *et al.* Discriminating ferrotoroidic from antiferrotoroidic ground states using a 3d quantum spin sensor. *npj Quantum Materials* **9**, 106 (2024).

[14] Lunghi, A. A unified ab initio theory of spin-phonon relaxation and decoherence uncovers fast dephasing in magnetic molecules. *Science Advances* **12**, eaeb3868 (2026).

[15] Ding, Y.-S. *et al.* Field-and temperature-dependent quantum tunnelling of the magnetisation in a large barrier single-molecule magnet. *Nature Communications* **9**, 3134 (2018).

[16] Staab, J. K., Rahman, M. K. & Chilton, N. F. Intramolecular bridging strategies to suppress two-phonon raman spin relaxation in dysprosocenium single-molecule magnets. *Physical Chemistry Chemical Physics* **26**, 17539–17548 (2024).

[17] Martínez, P. G. d. A. *et al.* Boosting terahertz generation in laser-field ionized gases using a sawtooth wave shape. *Physical review letters* **114**, 183901 (2015).

[18] Hasselbach, K., Veauvy, C. & Mailly, D. Microsquid magnetometry and magnetic imaging. *Physica C: Superconductivity* **332**, 140–147 (2000).

[19] Wernsdorfer, W. From micro- to nano-squids: applications to nanomagnetism. *Superconductor Science and Technology* **22** (2009).

[20] Chudnovskii, A., Mogilevskii, B. & Surin, V. Investigation of the thermal conductivity of molecular crystals. *Journal of engineering physics* **19**, 1295–1298 (1970).

[21] Shulumba, N., Hellman, O. & Minnich, A. J. Lattice thermal conductivity of polyethylene molecular crystals from first-principles including nuclear quantum effects. *Physical review letters* **119**, 185901 (2017).

[22] Dahule, R., Oqmhula, K., Maezono, R. & Hongo, K. Physics-informed data-driven discovery of polymer crystals with high thermal conductivity. *ACS Applied Polymer Materials* **7**, 1431–1439 (2025).

[23] Ready, J. F. *Effects of High-Power Laser Radiation* (Academic Press, 1971).

[24] Eaton, S. M. *et al.* Heat accumulation effects in femtosecond laser-written waveguides with variable repetition rate. *Optics Express* **13**, 4708–4716 (2005).

[25] Carslaw, H. S. & Jaeger, J. C. *Conduction of Heat in Solids* 2 edn (Oxford University Press, 1959).

[26] Quach, J. Q. *et al.* Superabsorption in an organic microcavity: Toward a quantum battery. *Science advances* **8**, eabk3160 (2022).

[27] Wall, J. V. & Jenkins, C. R. *Practical statistics for astronomers* (Cambridge University Press, 2012).
