# Supplementary material for: Finite-temperature toroidal moment amenable to direct observation in an Fe10Dy10 molecular ring
Source: Nat Commun. 2026 Jul 24;17:9030. doi: 10.1038/s41467-026-75612-6 (PMC13507247; doi:10.1038/s41467-026-75612-6)
Supplement: Supplementary file 2 [file 41467_2026_75612_MOESM2_ESM.pdf]

nature portfolio

Peer Review File

Finite-Temperature Toroidal Moment Amenable to Direct Observation in an $Fe_{10}Dy_{10}$ Molecular Ring

Corresponding Author: Professor Alessandro Soncini

This file contains all reviewer reports in order by version, followed by all author rebuttals in order by version.

Version 1:

Reviewer comments:

Reviewer #1

(Remarks to the Author)
In the manuscript, Soncini et al, report synthesis and magnetic characterization of Fe10Dy10 molecular magnets, focusing on their toroidal ground state and providing an in-depth theoretical study of them. Toroidal states in magnetism attract a lot of attention because of their magnetoelectric properties and correspondence to the fourth vectorial ordering of matter characterized by broken time and inversion symmetries. The family of ring-shaped molecular magnets is a famous playground for such studies, offering pure toroidal states and demonstrating a lot of exciting quantum physics. The authors motivated the study by a potentially large toroidal moment provided by Fe10Dy10 rings. The manuscript provides DFT analysis of the equilibrium states of the magnet. Predicted temperature and field characteristics are in good agreement with the experimental data, suggesting Fe10Dy10 to be a promising platform for further studies of ferrotoroidic states generally looking correct. The results sound interesting, but I would like to clarify certain points before drawing a recommendation for Nature Communications.

Firstly, the abstract sounds a bit overselling. Namely, I expected direct evidence of the toroidal state measured in Fe10Dy10 and demonstration of the access to the toroidal susceptibility \xi via femtosecond laser. The first point I will discuss below. The second point currently belongs only to the outlook, and regarding low temperatures of the magnetic ordering, it is not apparent whether it is experimentally feasible or not without additional references/discussions.

In the abstract and the main text, the authors name toroidal states as non-magnetic several times. Probably, it is a tradition for the community. However, a more general understanding of the term “non-magnetic” is rather the absence of magnetic ordering at all, while the _compensated_ magnetic ordering is actually in the focus. I would recommend tuning the wording, keeping it more accessible for a wide audience.

Moreover, in the abstract, it is not obvious why a high-dimensional Hilbert space is highlighted. At least, the technical procedure for how to deal with it is not drawn as one of the key results of the study. Without the direct link with the complexity to deal with a large amount of degrees of freedom, it does not sound like something special, e.g., one can always refer to continuous models with the infinite degrees of freedom.

To summarize these aspects, I would like to suggest the authors tune the presentation of messages they address in the manuscript at the very beginning to solidify the introduction.

To continue with the main text, Eq. (1) misses the definition of the origin. The toroidal moment is well-defined only for the compensated part of spin ordering, as it is pointed out in Ref. [3] and the previous publication of its authors [DOI:10.1103/physrevb.76.214404]. In this respect, my understanding of results misses the discussion of how the excitations shown in Fig. 2 and discussed regarding the finite magnetic moment for Fig. 3 alter the total toroidal moment \tau. One can expect that \tau decreases from its maximum at the strictly toroidal state, but how much? Basically, the appearance of the value of toroidal moment and its change under external conditions would substantially strengthen the manuscript.

In Fig. 3, the authors compare parameter-free simulations with the experimental data, and they are in perfect agreement. As far as I understood, this is one of the key points to prove the toroidal state of the substance under investigation. Could the authors discuss the robustness of this comparison? Say, if another more or less toroidal state is considered, will its field and temperature dependence substantially differ from the experimental data to tell that the presented results are the only

possible explanation?

Section 1.6 currently looks incomplete. It's only theoretical, without comparison with the experimental support. Of course, generating curl of B at the nanoscale is quite a challenging task, but if these messages cannot be incorporated into the direct proof of the presence of toroidal state in a real sample, then this part rather belongs to the discussion. Also, I'm not sure if the toroidal susceptibility has been introduced in the manuscript for the first time, see, e.g., [DOI:10.2478/muma-2014-0002], where the inverse quantity comes in the Landau theory [Eq. (81)] scaling the magnitude of T. Also, there was a quantum-mechanical calculation of the toroidal moment in [DOI:10.1103/physrevb.97.134423] starting from a similar idea. Potentially, it may be worth noting it in the manuscript for completeness of the literature review.

Lastly, regarding the major questions for the manuscript, I wonder about the uniformity of the single-crystal magnetic state. Do the authors expect that it is in the single-domain toroidal state, and if yes, why? It would be instructive to provide a comment on how to achieve a single-domain toroidal state and what signatures of multiple domains might be expected in measurements.

In the introduction, I'm not sure I got the message of the last sentence of the second paragraph: "Moreover, in chiral nanostructures, where parity symmetry is intrinsically broken, this dual character allows magnetic fields to generate a toroidal polarization directly, with magnetic and toroidal responses superimposed." Taking into account that the manuscript and literature distinguish the uniform magnetic fields and field curls, this sentence sounds rather like a possibility to create the toroidal state with the uniform field.

Could the authors provide an explanation of the elliptical shape of the molecule? Probably, a rather naive thinking about such essentially quantum SMT (still, well quantified in a micromagnetic model) may suggest a strictly circular shape, see, e.g. [DOI:10.1103/PhysRevB.99.014404]. So, what's the mechanism to break the rotational symmetry?

Schematics of the molecule given in different figures miss the Cartesian reference frame. To understand their orientation in space. Also, many panels miss legends, e.g. Fig. 3b, Fig. 4, Fig. 5a.

I would suggest making the x axis in Fig. 2a,c more clear.

Reviewer #2

(Remarks to the Author)
Reviewer Report

Summary

This manuscript presents a compelling and largely well-substantiated framework for finite-temperature toroidal physics in a large 3d–4f molecular ring, and introduces a useful linear-response quantity, the toroidal susceptibility ξ, to rationalise how a formally non-magnetic toroidal ground state can nonetheless yield an appreciable macroscopic toroidal polarisation at experimentally accessible temperatures. The combination of ab initio parameterisation with an efficient transfer-matrix treatment is technically strong, and the manuscript is generally clear and well organised.

In my view, the core claims are supported at the current level, and the work is suitable for publication after **minor revisions** that mainly tighten phrasing, improve "measurement-readiness" of the proposal, and clarify a few modelling and electromagnetic assumptions.

Recommendation

Minor revision. No fundamental re-analysis or new experiments are required, but the authors should address the points below to align the strength of the wording with what is quantitatively demonstrated and to improve accessibility for a broad Nature Communications readership.

Essential minor revisions

1) Calibrate the "direct observation" wording and add a concise readout statement

The manuscript motivates an experimentally realistic route to lifting the degeneracy of opposite toroidal chiralities via $\nabla \times B$ under ultrafast driving. This is convincing as a feasibility argument. To avoid overstating the outcome, I suggest the authors slightly soften or qualify "direct observation" in the title/abstract/main text unless a specific readout is clearly articulated.

A minimal, minor-revision-compliant fix is to add a short paragraph stating:

* what physical observable is expected to change upon inducing toroidal polarisation (even if only proposed, not yet implemented), and
* what qualitative signature would distinguish toroidal polarisation from conventional magnetic or thermal effects.

This can be done without adding new data, for example by naming a primary candidate observable (optical probe, symmetry-based nonlinear response, or magnetoelectric-type signal) and explaining, at a high level, what signature is expected.

2) Make ξ operational, clarify units, and provide one “worked example” mapping $\nabla\times B$ to $\langle\tau\rangle$

ξ is a strong conceptual contribution, but readers will benefit from one explicit bridge from definition to predicted magnitude. Please add:

* a brief dimensional analysis or unit convention for ξ, and
* one representative calculation, using your own estimated $\nabla\times B$, to report the predicted induced $\langle\tau\rangle$ (or polarisation fraction relative to saturation) at a few temperatures.

This can be presented as a short inset in the main text or a short Supplementary Note. It should not require additional computations beyond what is already in hand, since it is essentially a re-expression of existing results.

3) Refine “parameter-free” statements for precision

Because the lattice heat-capacity background includes fitted coefficients, I recommend revising “parameter-free” phrasing to something more precise, such as: “magnetic Hamiltonian parameters obtained from ab initio calculations without fitting to thermodynamic data; lattice background captured with a minimal empirical correction.”
This is a wording correction only, but it matters for reviewer and editor perception.

4) Add a brief robustness statement on the ab initio exchange parameterisation

The broken-symmetry DFT strategy with lanthanide substitution and scaling is reasonable and common for 4f systems. To pre-empt concerns, please add either:

* a short sensitivity statement (for example, qualitative invariance of the main conclusions under modest percent-level perturbations of the leading exchanges), or
* a concise justification of why the extracted ξ and the key low-energy features are not fine-tuned to a narrow parameter set.

This can be brief and qualitative, but it should be explicitly stated.

5) Clarify the electromagnetic driving assumptions (carrier vs envelope) and experimental boundary conditions

The ultrafast-pulse estimate is an important part of the “realistic conditions” message. A minor revision should clarify:

* whether $\partial E/\partial t$ is treated at the carrier level or via the pulse envelope, and why cycle-to-cycle cancellation does not wash out the effective driving of toroidal chirality, and
* what sample form is assumed (single crystal, oriented ensemble, pellet), including a short note on orientation averaging and how it impacts the expected macroscopic polarisation signal.

If a fully oriented sample is required, please state that explicitly and add one or two sentences suggesting a practical alignment route. This can remain at a proposal level.

6) Promote one key supporting result from the Supplementary Information into the main text

Some of the most convincing material that reconciles the toroidal ground state picture with dynamical magnetisation behaviour appears to be in the Supplementary Information. I suggest moving a representative panel (or adding a short “SI summary” paragraph) into the main text to reinforce the physical interpretation and make the narrative more self-contained.

Minor edits and presentation suggestions (non-essential but recommended)

1. Standardise unit conversions and explicitly relate quoted splittings to k_BT at the temperatures emphasised in the discussion.
2. Add a compact parameter table (Hamiltonian terms, numerical settings) and ensure it is easy to locate.
3. Consider a short “Data and code availability” note indicating where scripts or inputs for the transfer-matrix calculations can be accessed, if feasible.
4. Proofread the Supplementary Information for typographical issues and consistent notation.

Final assessment

The manuscript is conceptually strong, technically sophisticated, and well aligned with Nature Communications scope. Addressing the minor points above will improve precision, reproducibility, and the credibility of the proposed “direct observation” pathway without changing the substance of the results.

Reviewer #3

(Remarks to the Author)
Review of: "Finite-Temperature Toroidal Moment Amenable to Direct Observation in an $Fe_{10}Dy_{10}$ Molecular Ring"
Recommendation: [accept with major revisions]
Overall Assessment:
This manuscript presents a comprehensive theoretical investigation of toroidal moments in an icosanuclear $Fe_{10}Dy_{10}$ molecular ring, introducing the novel concept of toroidal susceptibility ξ and demonstrating the possibility of finite-temperature toroidal polarization. The work combines ab initio calculations (CAHF/CASCI-SO for lanthanide crystal fields, broken-symmetry DFT for exchange couplings) with an elegant transfer matrix approach to handle the ~62-billion-dimensional Hilbert space. The authors achieve good quantitative agreement with experimental magnetic and calorimetric data and propose an experimental pathway for direct observation of molecular toroidal moments using femtosecond laser pulses.
The manuscript makes significant contributions to the field of molecular magnetism and toroidal electrodynamics. The introduction of toroidal susceptibility as a thermodynamic response function is very useful, providing a quantitative framework for understanding finite-temperature toroidal properties. However, the manuscript has several weaknesses that should be addressed before publication.
1. Missing Magnetoelectric Coupling Analysis: A critical weakness is the absence of magnetoelectric (ME) coupling investigation despite the manuscript's emphasis on toroidal moments. The fundamental importance of toroidal moments lies in enabling linear ME coupling—electric control of magnetism—as correctly noted in the introduction [Spaldin et al., J. Phys.: Condens. Matter 20, 434203 (2008)]. However, the manuscript provides no analysis of ME coupling in $Fe_{10}Dy_{10}$: no calculation of the ME tensor α, no investigation of electric field effects on toroidal states, and no discussion of electric-field control as an alternative to the proposed magnetic field curl approach.
This omission is particularly significant given that: (1) recent studies have demonstrated ME coupling in related molecular systems [Yazback et al., J. Phys. Chem. A 127, 3814 (2023); Lewkowitz et al., Sci. Rep. 13, 2769 (2023)]; (2) electric field control would be more experimentally accessible than the proposed ultrafast laser configuration; and (3) ME coupling represents the primary technological advantage of toroidal systems. The manuscript would be substantially strengthened by adding computational investigation of ME effects and discussing electric-field manipulation of toroidal states.
2. Literature Coverage and Contextualization: The manuscript claims (page 3) that "toroidal properties have only been identified and discussed for specific molecular microstates, while the collective role of toroidal excitations within a molecular system and their contribution to a finite-temperature toroidal polarization have largely been ignored." This statement overlooks recent systematic theoretical investigations of toroidal moments in molecular systems. Specifically: Yazback et al. [J. Phys. Chem. A 127, 3814-3823 (2023)] performed comprehensive first-principles studies of toroidal moments in transition-metal trimers (Cr_3 and Cu_3), demonstrating: 1) Finite ground-state toroidal moments in Cu_3 (M_t = 0.895 ÅμB, 32% of maximum; 2) Toroidal moments in both ground and excited states; 3) Systematic parameter-space exploration identifying >194,000 spin models with high toroidal moments (>99% of maximum); 4) Design principles showing most high-Mt models require specific exchange coupling patterns ($J_{xx} > 0$, $J_{yy} < 0$, $J_{zz} < 0$) and antisymmetric exchange; and 5)Magnetoelectric coupling effects on toroidal moments. This work directly addresses the claimed gaps regarding collective excitations and design principles for molecular toroidal moments and should be cited and discussed.
2. Experimental Validation of Main Claims: The manuscript title and abstract claim the toroidal moment is "amenable to direct observation." The proposal relies on order-of-magnitude estimates (Eq. 10, page 12) and several unverified assumptions. Specific concerns: a) Laser-induced heating effects: The estimated fluence (0.03 J/cm²) and 10 fs pulse duration could cause significant heating. Issues need to be considered include: temperature rise during the pulse, thermal damage threshold for the molecular crystal, effects on the delicate $Fe_{10}Dy_{10}$ structure, etc. b) Toroidal relaxation time: The authors assume "sufficiently slow toroidal relaxation times" (page 12) but provide no estimate or discussion. What is the expected relaxation time? How does it compare to the pulse duration and measurement timescale? c) Signal detection: How would the toroidal polarization be measured experimentally? What is the expected signal-to-noise ratio? What detection methods are proposed?
The authors should consider: substantially expand the discussion of experimental challenges, provide more rigorous estimates of heating/damage, relaxation times, and detection schemes, and appropriately temper claims from "amenable to direct observation" to "predicted to be observable under specific conditions pending experimental verification"
Minor issues:
1. Authors shows that as temperature approaches to zero, toroidal moment approaches a maximum value. The zero temperature moment can be evaluate by applying a perturbation that lifts the energy degeneracies. Is there a particular reason to use the method in the manuscript?
2. Metastability: Is the toroidal ground state truly the global ground state or a metastable configuration?

Version 2:

Reviewer comments:

Reviewer #1

(Remarks to the Author)
The manuscript is substantially improved after the revision, and I can recommend it for publication in Nature Communications after several minor updates:

- The abstract and the introduction should be formulated in such a way that micro-SQUID will not come as the method used in this work. To my feeling, it is more suitable for the Discussion section.
- I'm not convinced that the toroidal susceptibility is a new quantity that has nothing in common with the previously discussed

quantities (Refs. 52, 53): all of them are naturally introduced as in the Landau theory (see Landau, Theoretical Physics, Vol. 8). But of course, a reader of the manuscript appreciates the calculation specific for the given physical system, which is an important achievement.
- The magnetoelectric tensor, which is given in Eq. (14), seems to have too many significant digits that are far beyond the accuracy of the previously used estimates, and, probably, not relevant for any potential experimental access. As a minor remark, it would be useful to mention the purely toroidal antisymmetric part of the magnetoelectric tensor in the spirit of discussion by Spaldin et al, JPCM 20 (2008), Eqns. (40-41).

(Remarks on code availability)

Reviewer #2

(Remarks to the Author)
General assessment
The revised manuscript is substantially improved, and my primary concerns from the first review have been addressed in a technically meaningful way. In particular, the authors have (i) introduced an explicit magnetoelectric (ME) response framework with an ab initio–informed ME tensor and a concrete ME readout channel, and (ii) provided a quantitatively closed "prepare–accumulate–read out" protocol for nonequilibrium toroidal polarisation driven by a temporally asymmetric near-infrared waveform, including detectability and heating estimates. The new Section 1.7 and Figure 6 materially strengthen the "amenable to direct observation" narrative.
I consider the manuscript close to publishable. I recommend minor revision, limited to a small number of clarifications and one bibliographic correction.
Point-by-point evaluation of the authors' responses to my main comments
1) Magnetoelectric coupling analysis (previously missing)
Assessment: This item is satisfactorily addressed. The added ME framework is a major improvement and is conceptually well aligned with the technological and fundamental motivation for toroidal states.
Minor remaining clarification (optional): The adopted static readout field magnitude is very large; a brief note on practical realisation (for example micro/nano-electrode gap geometry, thin sample, pulsed readout to avoid breakdown) and explicit mention of linear scaling with the readout field would improve experimental transparency.
2) Literature positioning and scope claims
Assessment: The literature positioning is improved and largely acceptable. However, one remaining sentence in the Introduction still reads overly absolute (stating that the collective finite-temperature role of toroidal excitations "has largely been ignored"), which could be interpreted as dismissive of quantitative efforts in adjacent subfields.
Minor request: Please soften or qualify this statement explicitly to the specific regime addressed here (strong SOC, non-frustrated SMTs, discrete many-state manifolds, and an operational finite-temperature linear-response/readout framework).
3) Direct observability and feasibility (heating, relaxation, detectability)
Assessment: This item is largely addressed and is now presented as a quantitative proof-of-principle protocol. A brief sensitivity note on the heating estimate (for example, scaling of ΔT with absorbed fraction) would further strengthen the "realistic conditions" claim but is not essential if space is limited.
Additional required correction (bibliography)
The revised manuscript already cites the recent Nature Chemistry paper on homochiral toroidal spin states in Dy(III)-based SMTs (currently listed as Ref. 16). Please add the DOI to the reference entry to match standard citation practice and to facilitate direct access to this key, very recent result:
DOI: 10.1038/s41557-026-02070-4
Recommendation
Minor revision. After (i) adding the DOI for Ref. 16 and (ii) making the small wording adjustment in the Introduction to avoid overly absolute scope claims, I would support acceptance.

(Remarks on code availability)

Reviewer #3

(Remarks to the Author)
In response to my original report ("accept with major revisions, requires re-review"), the authors have carried out a substantial body of genuinely new work rather than cosmetic edits. Every change described in the response letter is supported by content I was able to locate in the revised manuscript and Supplementary Information: an entirely new Section 1.7 with a new Figure 6, two new Methods subsections (3.6 and 3.7), and three new Supplementary Notes (6, 7 and 8) containing complete derivations, tables and figures.
All three of my major concerns and both of my minor points are now substantively engaged. The magnetoelectric coupling analysis I requested has been implemented from first principles; the experimental-viability concerns (heating, relaxation, detection) are addressed with quantitative, multi-step estimates that go well beyond the original order-of-magnitude argument; and the literature contextualisation has been corrected. A small number of residual caveats remain, but these are largely inherent to a proof-of-principle theoretical proposal and are transparently disclosed by the authors. In my view the manuscript is now suitable for publication.

Remaining suggestion (minor, no further review needed)
A single sentence acknowledging the sensitivity of the heating estimate to the assumed near-IR extinction coefficient would make the analysis more complete. This is the only point I would still raise, and it can be handled at the authors' discretion.
Summary and Recommendation
Scientific Quality: VERY GOOD — strengthened by the revision.
Appropriateness for Nature Communications: GOOD FIT.

(Remarks on code availability)
The author claims --

The code is currently undergoing active development… For this reason, we prefer not to maintain a public release version at this stage. Nevertheless, we are happy to provide… reasonable access to scripts or input files upon request.

Open Access This Peer Review File is licensed under a Creative Commons Attribution 4.0 International License, which permits use, sharing, adaptation, distribution and reproduction in any medium or format, as long as you give appropriate credit to the original author(s) and the source, provide a link to the Creative Commons license, and indicate if changes were made.
In cases where reviewers are anonymous, credit should be given to 'Anonymous Referee' and the source.
The images or other third party material in this Peer Review File are included in the article's Creative Commons license, unless indicated otherwise in a credit line to the material. If material is not included in the article's Creative Commons license and your intended use is not permitted by statutory regulation or exceeds the permitted use, you will need to obtain permission directly from the copyright holder.
To view a copy of this license, visit https://creativecommons.org/licenses/by/4.0/

Response to referees: Finite-Temperature Toroidal Moment Amenable to Direct Observation in an $Fe_{10}Dy_{10}$ Molecular Ring

Alessandro Soncini,[1,*] Kieran Hymas,[2] Jonas Braun,[3,4,5] Yannik F. Schneider,[3] Simone Calvello,[1] Amer Baniodeh,[3] Yanhua Lan,[3] Wolfgang Wernsdorfer,[5,6] Marco Affronte,[7] Christopher E. Anson,[3] and Annie K. Powell[3,4,5,*]

[1]*Department of Chemical Sciences, University of Padova, Via Marzolo 1, Padova, 35123, Italy*
[2]*Commonwealth Scientific and Industrial Research Organisation, (CSIRO), Clayton, Victoria, 3168, Australia*
[3]*Institute of Inorganic Chemistry, Karlsruhe Institute of Technology (KIT), Kaiserstr. 12, Karlsruhe, 76131, Germany*
[4]*Institute of Nanotechnology, Karlsruhe Institute of Technology (KIT), Kaiserstr. 12, Karlsruhe, 76131, Germany*
[5]*Institute for Quantum Materials and Technologies, Karlsruhe Institute of Technology (KIT), Kaiserstr. 12, Karlsruhe, 76131, Germany*
[6]*Physikalisches Institut, Karlsruhe Institute of Technology (KIT), Kaiserstr. 12, Karlsruhe, 76131, Germany*
[7]*Dipartimento di Scienze Fisiche, Informatiche e Matematiche, University of Modena and Reggio Emilia, Via Campi 213A, Modena, 41125, Italy*

(Dated: May 22, 2026)

We would like to extend our thanks to all three referees for providing their time and expertise in reviewing our paper. The referees' comments are reproduced below in black, our responses are given in blue, and the corresponding modifications/additions to the manuscript are highlighted in red.

REVIEWER 1

In the manuscript, Soncini et al, report synthesis and magnetic characterization of $Fe_{10}Dy_{10}$ molecular magnets, focusing on their toroidal ground state and providing an in-depth theoretical study of them. Toroidal states in magnetism attract a lot of attention because of their magnetoelectric properties and correspondence to the fourth vectorial ordering of matter characterized by broken time and inversion symmetries. The family of ring-shaped molecular magnets is a famous playground for such studies, offering pure toroidal states and demonstrating a lot of exciting quantum physics. The authors motivated the study by a potentially large toroidal moment provided by $Fe_{10}Dy_{10}$ rings. The manuscript provides DFT analysis of the equilibrium states of the magnet. Predicted temperature and field characteristics are in good agreement with the experimental data, suggesting $Fe_{10}Dy_{10}$ to be a promising platform for further studies of ferrotoroidic states generally looking correct. The results sound interesting, but I would like to clarify certain points before drawing a recommendation for Nature Communications.

We thank the Reviewer for the careful reading of the manuscript and for the very constructive comments. We are pleased that the Reviewer finds the results interesting and recognizes the $Fe_{10}Dy_{10}$ ring as a promising platform for finite-temperature toroidal physics. We have carefully revised the manuscript to clarify the physical interpretation, improve accessibility for a broader readership, and strengthen the discussion of finite-temperature toroidal response and of a detailed protocol for the generation and readout of toroidal polarization which now addresses the issue of direct experimental observability in a more focused and quantitative manner.

Firstly, the abstract sounds a bit overselling. Namely, I expected direct evidence of the toroidal state measured in $Fe_{10}Dy_{10}$ and demonstration of the access to the toroidal susceptibility ξ via femtosecond laser. The first point I will discuss below. The second point currently belongs only to the outlook, and regarding low temperatures of the magnetic ordering, it is not apparent whether it is experimentally feasible or not without additional references/discussions.

We agree that in the original version of the manuscript the discussion of "direct observability" was still somewhat compressed relative to the scope of the underlying proposal. Rather than substantially softening the central claim, however, we have responded by significantly expanding the quantitative framework supporting it.
In particular, the revised manuscript now contains a substantially developed treatment of the ultrafast-driven nonequilibrium toroidal accumulation mechanism and of the associated magnetoelectric readout protocol (see in particular: new Section 1.7 of main text, Subsections 3.6 and 3.7 of the Methods section, and Supplemenatry Notes 6 and 7). These additions include: (i) many-state dissipative dynamics simulations under a temporally asymmetric near-infrared pulse train, (ii) quantitative magnetoelectric readout signal estimates, (iii) experimentally realistic detectability estimates, and (iv) quantitative estimates of thermal accumulation under repeated-pulse driving.
As a result, we believe that the revised manuscript now provides a significantly more quantitatively grounded proof-of-principle framework for the preparation, accumulation and experimental detection of nonequilibrium toroidal polarisation

in $Fe_{10}Dy_{10}$.
At the same time, we have carefully revised several passages in the abstract, introduction and conclusions in order to better distinguish between experimentally established results and the current proposal.

Changes:

- New Section 1.7 devoted to ultrafast-driven toroidal accumulation and magnetoelectric readout, including new Figure 6
- Completely rewritten "Discussion and Conclusions" section
- New Methods subsection 3.6
- New Methods subsection 3.7
- New Supplementary Note 6 on the microscopic magnetoelectric response framework
- New Supplementary Note 7 on ultrafast dynamics, thermal accumulation and detectability estimates

In the abstract and the main text, the authors name toroidal states as non-magnetic several times. Probably, it is a tradition for the community. However, a more general understanding of the term "non-magnetic" is rather the absence of magnetic ordering at all, while the compensated magnetic ordering is actually in the focus. I would recommend tuning the wording, keeping it more accessible for a wide audience.

We agree that the expression "non-magnetic" may potentially be misleading for a broader readership, since the relevant states correspond to magnetically compensated toroidal configurations rather than to an absence of magnetic ordering altogether. We have therefore revised the wording throughout the manuscript in order to clarify this distinction more explicitly. In particular, expressions such as "non-magnetic ground state" and "non-magnetic toroidal doublet" have been replaced by formulations explicitly referring to compensated magnetic configurations or vanishing net magnetic dipole moment.

Changes:

- Revised the wording throughout the manuscript to replace potentially ambiguous expressions such as "non-magnetic toroidal state" or "non-magnetic ground state" with more explicit formulations referring to magnetically compensated toroidal configurations or vanishing net magnetic dipole moment.

Moreover, in the abstract, it is not obvious why a high-dimensional Hilbert space is highlighted. At least, the technical procedure for how to deal with it is not drawn as one of the key results of the study. Without the direct link with the complexity to deal with a large amount of degrees of freedom, it does not sound like something special, e.g., one can always refer to continuous models with the infinite degrees of freedom.
To summarize these aspects, I would like to suggest the authors tune the presentation of messages they address in the manuscript at the very beginning to solidify the introduction.

We agree that in the original version of the manuscript the relevance of the very large Hilbert space dimension was not articulated sufficiently clearly in the abstract and introductory discussion. The point we intended to emphasize is twofold. First, the unusually large number of low-energy states is physically central to the finite-temperature toroidal behaviour discussed in this work. In $Fe_{10}Dy_{10}$, toroidal character is not restricted to a single isolated molecular microstate, but extends across a very large manifold of thermally accessible states. As a consequence, billions of many-body states contribute collectively to the finite-temperature toroidal response and toroidal susceptibility. To the best of our knowledge, this aspect has not previously been analysed quantitatively for a molecular toroidal system.
Second, the treatment of such a large correlated many-body space is itself technically nontrivial in a finite molecular system lacking translational symmetry. While formally infinite Hilbert spaces can often be handled analytically or numerically in effective one-electron or translationally invariant models, the explicit treatment of strongly correlated non-collinear spin systems of this size is substantially more challenging in the molecular context.
Although the decorated-Ising transfer-matrix framework itself builds on previous literature already cited in the manuscript, the present work required substantial system-specific methodological developments. In particular, we introduced an ab initio parameterisation of the full model together with perturbative corrections accounting explicitly for interactions between neighbouring quantum decorations, which would otherwise be computationally intractable within exact diagonalisation

approaches. As discussed in the revised manuscript and illustrated in Figure 3a and Figure 5b, these corrections play an important quantitative role in reproducing the low-field magnetic response and toroidal properties of $Fe_{10}Dy_{10}$.
To clarify these points, we have revised the abstract and introductory discussion to better explain the physical and methodological significance of the large Hilbert-space dimension in the present study.

Changes:

- The Abstract now explicitly clarifies the physical and methodological significance of the large low-energy manifold, referring to the "$\sim$ 62-billion-dimensional low-energy manifold with pervasive toroidal character, rendered computationally tractable via an *ab initio*-informed transfer-matrix framework with perturbative corrections."
- The Introduction now explicitly highlights the methodological framework through the sentence: "Combining multi-reference *ab initio* calculations with a tailor-made perturbatively corrected transfer-matrix approach describing a quantum-decorated non-collinear Ising chain ..."

To continue with the main text, Eq. (1) misses the definition of the origin. The toroidal moment is well-defined only for the compensated part of spin ordering, as it is pointed out in Ref. [3] and the previous publication of its authors [DOI:10.1103/physrevb.76.214404]. In this respect, my understanding of results misses the discussion of how the excitations shown in Fig. 2 and discussed regarding the finite magnetic moment for Fig. 3 alter the total toroidal moment τ. One can expect that τ decreases from its maximum at the strictly toroidal state, but how much? Basically, the appearance of the value of toroidal moment and its change under external conditions would substantially strengthen the manuscript.

The Reviewer raises two points: the origin dependence of toroidal response, and the evolution of toroidal character under thermal population of excited states.
We now clarify in the revised manuscript that all toroidal matrix elements are evaluated with respect to the molecular inertia frame, and provide a brief general comment on the reasons underlying this choice, also citing relevant literature.
More importantly, the second issue raised by the Reviewer — namely how the toroidal character evolves under thermal excitation — is precisely one of the central motivations for introducing the finite-temperature toroidal susceptibility ξ. In the revised manuscript we now make this connection more explicit by discussing both the temperature dependence of ξT in Fig. 5b and representative induced toroidal moments obtained from the linear-response relation between $\xi_{\alpha\beta}$ and the applied magnetic-field curl. These estimates directly quantify how thermal population of excited states progressively reduces the finite-temperature toroidal polarisation relative to the fully saturated ground-state toroidal moment.
Changes:

- In Section 1.6, soon after Eqs. (6) and (7), we added:

  > "Here all toroidal matrix elements are evaluated with respect to the molecular inertia frame. In general, toroidal moments are strictly origin independent only for magnetically compensated states.[3,9,52] In this system, the maximally toroidal ground-state is thus rigorously origin independent, whereas finite-temperature corrections to $\xi_{\alpha\beta}$ arising from thermally populated excited states can inherit a corresponding origin dependence. The molecular inertia frame nevertheless provides a useful reference frame both for analysing single-molecule toroidal structure-property correlations and for constructing a compact multipolar energy expansion of the linear response to locally constant magnetic-field curls in single-molecule settings, or to globally constant magnetic-field curls in molecular ensembles."

- Same Section, after introducing the constitutive relation Eq. (7) between toroidal polarisation and magnetic-field curl, we now explicitly discuss the magnitude of the induced finite-temperature toroidal polarisation predicted by $\xi_{\alpha\beta}$, including representative estimates at $T = 0.1$, 0.5, and 1 K, together with the corresponding fully saturated ground-state toroidal moment extracted from the $T \to 0$ limit of ξT. See the added discussion:

  > In the limit $T \to 0$, $\xi_{\alpha\alpha} T = |\langle 0|\, \tau_\alpha \,|0\rangle|^2 / k_B$, hence the low-temperature value $\xi_{zz} T \sim 2.5 \times 10^6$ cm^{-1} K a_0^2 T^{-2} yields a ground-state toroidal moment $|\langle 0|\, \tau_\alpha \,|0\rangle| \sim 1.3 \times 10^3\ a_0 \mu_B$.
  > Using the peak magnetic-field curl estimated for the ultrafast near-IR driving protocol discussed in Section 1.7, $(\nabla \times \mathbf{B})_z \sim 5 \times 10^{-4}$ T nm^{-1} $\sim 2.65 \times 10^{-5}$ T a_0^{-1}, Eq. (7) predicts induced toroidal moments $\tau_\alpha(T) \sim 6.1 \times 10^2$, $\sim 5.3 \times 10^1$, and $\sim 1.5 \times 10^1\ a_0 \mu_B$ at $T = 0.1$, 0.5, and 1 K, respectively, showing that even at 0.1 K the induced finite-temperature toroidal polarisation remains about 45% of the fully saturated ground-state toroidal moment, before progressively degrading at higher temperatures.

In Fig. 3, the authors compare parameter-free simulations with the experimental data, and they are in perfect agreement. As far as I understood, this is one of the key points to prove the toroidal state of the substance under investigation. Could

the authors discuss the robustness of this comparison? Say, if another more or less toroidal state is considered, will its field and temperature dependence substantially differ from the experimental data to tell that the presented results are the only possible explanation?

We agree that the robustness of the comparison between the ab initio-parameterised simulations and the experimental data is an important issue.
First, we note that the original version of the manuscript already contained a partial robustness analysis of the magnetic model. In particular, the inset of Fig. 3a explicitly compared the magnetic response obtained with and without the frustrating antiferromagnetic Fe–Fe next-nearest-neighbour exchange interaction. Importantly, this interaction does not reinforce the toroidal regime, but partially destabilises it by favouring low-energy magnetic excitations over the ideal non-collinear ferrotoroidic configuration, as also reflected by the suppression of the low-temperature toroidal susceptibility shown in Fig. 5b. Nevertheless, inclusion of this interaction substantially improves the agreement between theory and experiment. This indicates that the experimentally observed behaviour is not reproduced simply by enforcing an idealised toroidal limit, but rather emerges from a physically realistic competition between toroidal and magnetic low-energy tendencies.
More importantly, the revised manuscript now includes an independent statistical χ^2 robustness analysis of the dominant exchange parameters (new Supplementary Note 8). In this analysis, the leading exchange constants were independently fitted against the experimental magnetic susceptibility data using a three-parameter model. The resulting best-fit values remain fully consistent, within statistical uncertainty, with the broken-symmetry DFT estimates employed in the transfer-matrix simulations.
We therefore believe that the revised manuscript now demonstrates that the experimentally observed thermodynamic behaviour is robustly associated with the physically realistic exchange regime identified by the ab initio parameterisation, rather than arising from a narrowly fine-tuned or artificially constrained toroidal model.

Changes:

- Added a new statistical χ^2 robustness analysis of the dominant exchange parameters in Supplementary Note 8.
- At the end of Section 1.2 (“DFT magnetic coupling constants”) we now write:

  “To further assess the robustness of these exchange parameters, we performed an independent statistical χ^2 analysis based on a three-parameter fit of the magnetic susceptibility data (Supplementary Note 8). The best-fit values, $J_A^{\rm fit} = 0.8^{+0.2}_{-0.15}$ cm^{-1}, $J_B^{\rm fit} = 0.6^{+0.7}_{-0.55}$ cm^{-1} and $J_C^{\rm fit} = -0.10^{+0.10}_{-0.05}$ cm^{-1}, are consistent with the broken-symmetry DFT estimates, which lie within the 68% confidence region of the fit. This independent analysis validates both the magnitude and sign of the dominant exchange interactions used in the *ab initio*-parameterised transfer-matrix model.”

Section 1.6 currently looks incomplete. It’s only theoretical, without comparison with the experimental support. Of course, generating curl of **B** at the nanoscale is quite a challenging task, but if these messages cannot be incorporated into the direct proof of the presence of toroidal state in a real sample, then this part rather belongs to the discussion. Also, I’m not sure if the toroidal susceptibility has been introduced in the manuscript for the first time, see, e.g., [DOI:10.2478/muma-2014-0002], where the inverse quantity comes in the Landau theory [Eq. (81)] scaling the magnitude of T. Also, there was a quantum-mechanical calculation of the toroidal moment in [DOI:10.1103/PhysRevB.97.134423] starting from a similar idea. Potentially, it may be worth noting it in the manuscript for completeness of the literature review.

We thank the Reviewer for pointing out previous literature discussing related finite-temperature toroidal-response quantities. We have now revised the introduction of Section 1.6 accordingly and explicitly discuss the relationship between the present $\xi_{\alpha\beta}$ tensor and earlier Landau-theory free-energy expansions for ordered phases, and spin-toroidization approaches for periodic solid-state systems, citing all the relevant literature.
In particular, we now clarify that the present $\xi_{\alpha\beta}$, while clearly related, differs conceptually from these previous constructions in that it is formulated explicitly as a finite-temperature molecular linear-response tensor describing the response of thermally populated toroidal states to an externally applied magnetic-field curl, rather than the response of a toroidally ordered bulk phase. The present implementation also differs technically from previous bulk approaches in that it explicitly treats a discrete many-state molecular spectrum without relying on translational symmetry or continuum approximations.
We nevertheless appreciate the Reviewer’s concern regarding the balance between theoretical response analysis and experimentally established observables. However, we regard Section 1.6 as an important theoretical contribution of the manuscript rather than material more appropriate for the Discussion section. To the best of our knowledge, this section introduces the first operational finite-temperature toroidal linear-response tensor for a non-interacting ensemble of

molecular units (SMTs) with thermally populated toroidal states, directly analogous to the Van Vleck magnetic susceptibility, while also providing explicit estimates of the induced finite-temperature toroidal polarisation expected under experimentally realistic magnetic-field curls.
At the same time, we agree that the previous version of the manuscript could have more clearly distinguished between experimentally established results and theoretically proposed toroidal-response protocols. The revised manuscript therefore substantially expands and clarifies the discussion of experimentally realistic nonequilibrium toroidal accumulation and magnetoelectric readout mechanisms (new Section 1.7 and associated Methods/Supplementary Notes), while also moderating some aspects of Section 1.6.
For instance we acknowledge that the previous subsection 1.6 title ("Evidence for a maximally toroidal ground state") could have suggested a stronger level of experimental validation than intended in the context of the original submission. We have therefore renamed Section 1.6 to "Finite-temperature molecular toroidal response" in order to better reflect its role as a theoretical finite-temperature response framework.

Changes:

- Renamed Section 1.6 from "Evidence for a maximally toroidal ground state" to: "Finite-temperature molecular toroidal response" in order to better reflect its role as a finite-temperature molecular response framework.
- Added the following discussion immediately before Eq. (5) in Section 1.6:

  > "To probe the toroidal properties of the SMT at finite temperature, we introduce the toroidal susceptibility tensor $\xi_{\alpha\beta}$, describing the linear response of the system to a magnetic-field curl $\nabla \times \mathbf{B}$. While quantities related to finite-temperature toroidal response have previously appeared in Landau-theory expansions of ordered states [49], or in calculations of spin toroidization in periodic systems beyond the linear-response regime [50], the present $\xi_{\alpha\beta}$ is defined as an explicit finite-temperature linear-response tensor, directly analogous to the Van Vleck magnetic susceptibility, and specifically formulated for *molecular* systems with discrete, thermally populated toroidal spectra:"

- Added an explicit operational discussion in Section 1.6 of the constitutive relation between induced toroidal polarisation and magnetic-field curl, including representative finite-temperature estimates of the induced toroidal moment using a realistic magnetic-field curl scale motivated by the ultrafast-driving protocol introduced later in new Section 1.7.

  > "In the limit $T \to 0$, $\xi_{\alpha\alpha} T = |\langle 0| \tau_\alpha |0\rangle|^2 / k_B$... ...showing that even at 0.1 K the induced finite-temperature toroidal polarisation remains about 45% of the fully saturated ground-state toroidal moment, before progressively degrading at higher temperatures."

- Added the entirely new Section 1.7 together with the associated Methods Sections 3.6 and 3.7, and Supplementary Notes 6 and 7, now explicitly focused on experimentally realistic protocols for the generation, accumulation and magnetoelectric readout of finite-temperature molecular toroidal polarisation.

Lastly, regarding the major questions for the manuscript, I wonder about the uniformity of the single-crystal magnetic state. Do the authors expect that it is in the single-domain toroidal state, and if yes, why? It would be instructive to provide a comment on how to achieve a single-domain toroidal state and what signatures of multiple domains might be expected in measurements.

The Reviewer raises an interesting point concerning possible toroidal domains in a bulk crystal. We clarify that the present work does not assume the existence of a spontaneously ordered single-domain ferrotoroidic phase. Rather, the finite-temperature response framework developed here treats the sample as an ensemble of non-interacting single-molecule toroics in the toroidal analogue of a conventional paramagnetic regime. In zero magnetic-field curl the equilibrium ensemble therefore remains globally unpolarised, with equal populations of opposite toroidal branches, while the finite-temperature tensor $\xi_{\alpha\beta}$ quantifies the induced toroidal polarisation generated by an external curl perturbation.
Similarly, the ultrafast protocol introduced in the revised manuscript does not rely on pre-existing toroidal domains, but dynamically generates a nonequilibrium population imbalance between opposite toroidal branches. Possible ferrotoroidic domain formation driven by intermolecular toroidal interactions therefore lies outside the scope of the present work. We have added a short clarification in the revised manuscript in order to avoid suggesting that a spontaneously ordered single-domain toroidal crystal is assumed.

Changes:

- Added the following clarification in Section 1.6 (paragraph following Eq. (6)) in order to explicitly distinguish the finite-temperature molecular toroidal response framework developed here from a spontaneously ordered ferrotoroidic phase/domain picture:

  "Eq. (6) therefore describes the finite-temperature molecular toroidal response of an ensemble of non-interacting SMTs in the toroidal analogue of a conventional paramagnetic regime, rather than a spontaneously ordered ferrotoroidic phase. In zero magnetic-field curl the equilibrium ensemble remains globally unpolarised, while the induced linear toroidal polarisation generated by an external curl perturbation is given by

  $$\tau_\alpha(T) = \sum_\beta \xi_{\alpha\beta}(T)\,(\nabla \times \mathbf{B})_\beta\,.$$

  "

In the introduction, I'm not sure I got the message of the last sentence of the second paragraph: "Moreover, in chiral nanostructures, where parity symmetry is intrinsically broken, this dual character allows magnetic fields to generate a toroidal polarization directly, with magnetic and toroidal responses superimposed." Taking into account that the manuscript and literature distinguish the uniform magnetic fields and field curls, this sentence sounds rather like a possibility to create the toroidal state with the uniform field.

We thank the Reviewer for pointing out that this sentence could be interpreted ambiguously. The intended meaning was not that a magnetic-field curl is unnecessary in general for generating a toroidal polarisation, but rather that in intrinsically chiral molecular systems, where parity symmetry is already broken structurally, a uniform magnetic field can in principle induce a finite toroidal polarisation through a mixed magnetic–toroidal response tensor.
By contrast, in non-chiral systems such as the present $Fe_{10}Dy_{10}$ ring, toroidal polarisation requires a perturbation that itself breaks inversion symmetry, such as a magnetic-field curl. In this sense, only the magnetic-field curl acts as the natural conjugate field to the toroidal moment for any system, whether chiral or not.
We now clarify this distinction explicitly in the Introduction and cite the recent work of Zhu *et al.*, which reported the first homochiral Dy-based single-molecule toroic crystal and experimental observations consistent with a homochiral toroidal ground-state interpretation.

Changes:

- Modified the final sentence of the second paragraph of the Introduction in order to clarify that, in chiral single-molecule toroic systems, uniform magnetic fields can in principle induce toroidal polarisation through mixed magnetic–toroidal response effects:

  "Moreover, in chiral nanostructures based on SMTs [14], where parity symmetry is intrinsically broken, uniform magnetic fields can in principle induce toroidal polarisation through mixed magnetic–toroidal response effects, with magnetic and toroidal responses partially superimposed [9]."

- Added the recent experimental work:

  "[14] Zhu, Z. *et al.* Homochiral toroidal spin state in Dy(III)-based single-molecule toroics. *Nature Chemistry* **18**, 1–10 (2026)."

Could the authors provide an explanation of the elliptical shape of the molecule? Probably, a rather naive thinking about such essentially quantum SMT (still, well quantified in a micromagnetic model) may suggest a strictly circular shape, see, e.g. [DOI:10.1103/PhysRevB.99.014404]. So, what's the mechanism to break the rotational symmetry?

We believe that the driving force towards ellipticity involves weak van der Waals interactions between the organic ligands in the ring. We have been investigating this aspect, but it lies outside the scope of the present paper (both thematically and for space reasons) and will be discussed in a subsequent publication. We note in particular that the ellipticity of the ring is moderate, with a ratio of major to minor axis $\frac{19.4\text{Å}}{15.9\text{Å}} \approx 1.22$. We thank the referee for the interesting reference [Gaididei et al., Phys. Rev. B **99** 014404 (2019)] where vortex magnetisation is studied in a classical model of a similar supramolecular ring system. Notably, Gaididei et al. show that elliptical ring structures can still support vortex magnetisation provided that the circumference of the ring L remains sufficiently large compared to the degree of ellipticity (see bottom-right region of their Fig. 2). In Fig. 2a of the main text, we show a rather small energy gap between the toroidal ground state and maximally magnetic first excited onion state/p-wave excitation of ≈ 0.26 cm^{-1}. In

the context of Gaididei et al.'s work, the proximity of $Fe_{10}Dy_{10}$ to this magnetic phase transition is likely related to the modest elliptical shape of the ring which is now explicitly stated in Section 1.4 of the main text.

Changes:

- Added the following sentence in Section 1.4 "Low-energy spectrum", immediately after the discussion of the non-*h*-wave excitations:

  "The modest ellipticity of the $Fe_{10}Dy_{10}$ ring (major/minor axis ratio ~ 1.22) may contribute to reducing the energy gap (~ 0.26 cm^{-1}) between the toroidal ground state (*s*-wave) and the competing onion-like (*p*-wave) magnetic excitations, consistent with previous studies of vortex states in elliptical magnetic rings. [48]"

- Added the reference:

  "Gaididei, Y. *et al.* Magnetization-induced shape transformations in flexible ferromagnetic rings. Phys. Rev. B **99**, 014404 (2019)."

Schematics of the molecule given in different figures miss the Cartesian reference frame. To understand their orientation in space. Also, many panels miss legends, e.g. Fig. 3b, Fig. 4, Fig. 5a.

We agree with the referee and have added Cartesian axes to relevant parts of Fig. 1 and Fig. 2. Furthermore, we have added missing plot legends to Fig. 3, Fig. 4 and Fig. 5.

Section 1.5—Comparison with experiments: Figure 1.

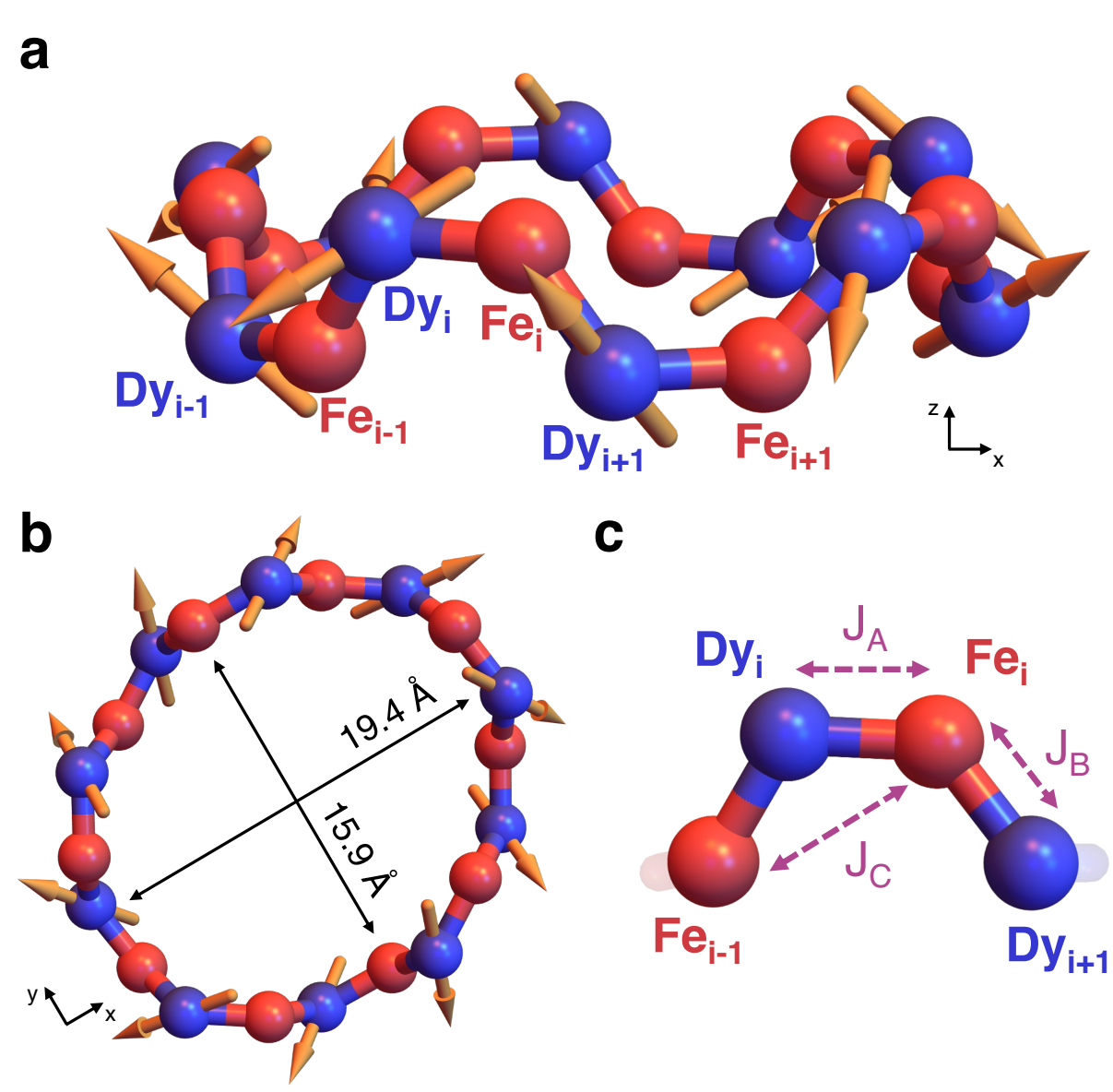

FIG. 1: Schematic depiction of the $Fe_{10}Dy_{10}$ paramagnetic skeleton. (a) Side profile of the wheel. Fe^{III} ions are depicted as red spheres and Dy^{III} ions as blue spheres. The *ab initio*-calculated principal magnetic axis $\mathbf{u}_i$ of each of the Dy^{III} ground doublets (reported in Table 2) are shown as orange arrows. (b) Birds eye view of $Fe_{10}Dy_{10}$ showing the 'long' and 'short' axes of the ellipse. (c) Exchange connectivity of a repeating fragment of the ring. The Dy-Fe exchange coupling between Dy_i^{III} and Fe_i^{III} and between Fe_i^{III} and Dy_{i+1}^{III} are J_A and J_B, respectively. The weaker Fe^{III}-Fe^{III} antiferromagnetic exchange coupling is J_C.

Section 1.5—Comparison with experiments: Figure 2.

Section 1.5—Comparison with experiments: Figure 3.

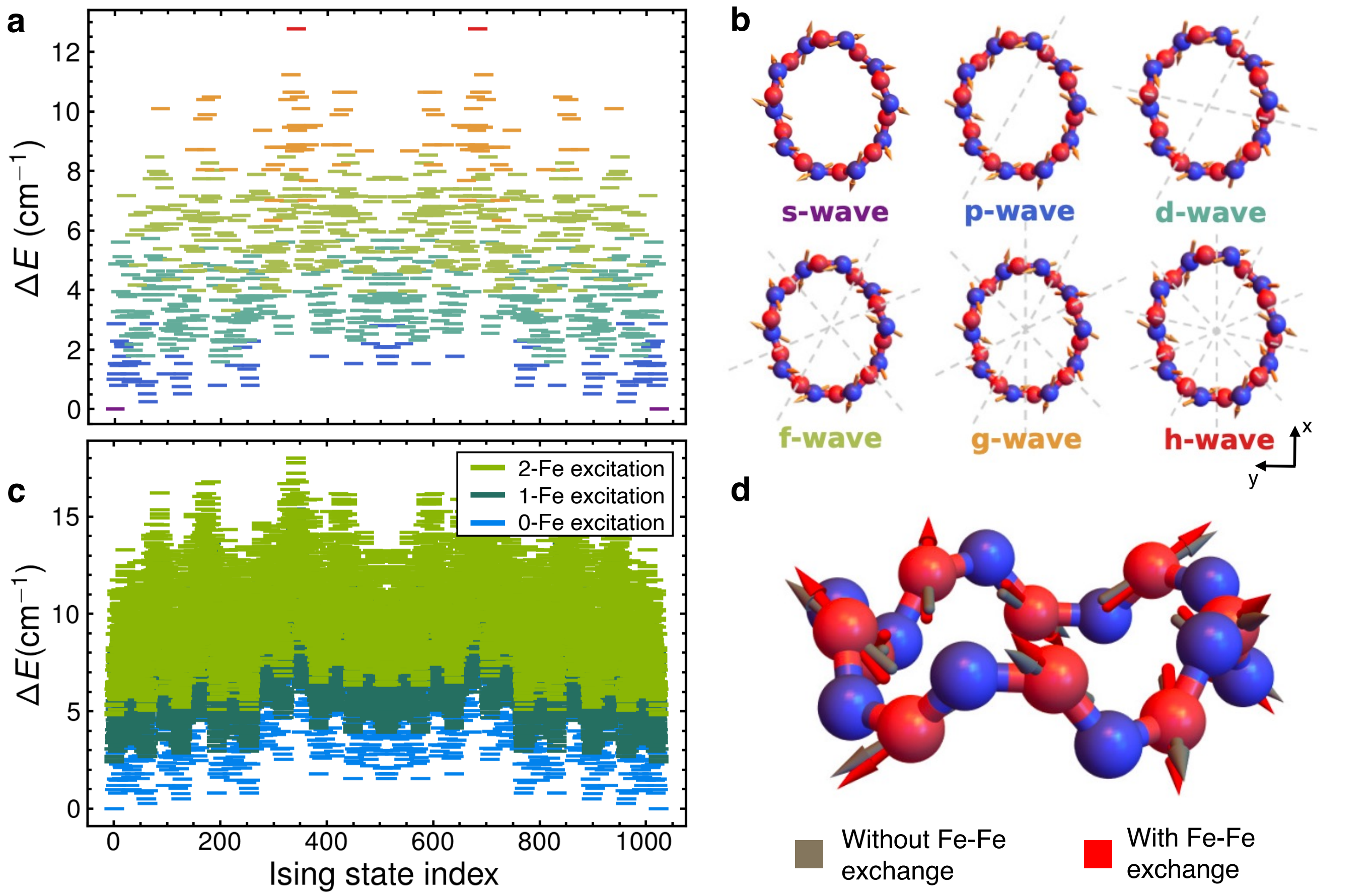

FIG. 2: Low energy spectrum of $Fe_{10}Dy_{10}$.(a) Lowest energy (b) Archetypal examples of s, p, d, f, g and h-wave Ising spin configurations analogous to the spin wave nomenclature. Colour coding corresponds to the band structure shown in (a). (c) Ground and excited state band structure of $Fe_{10}Dy_{10}$ from all possible one and two Fe^{III} excitations around the ring for fixed Dy^{III} Ising spin configurations. The Ising spin configurations are indexed by mapping the vector $\boldsymbol{\sigma}$ to binary representation and straightforwardly converting to a decimal representation offset by 1. (d) Calculated ground state Fe^{III} on-site spin expectation values for the toroidal Dy^{III} Ising spin configuration calculated with (red arrows) and without (grey arrows) Fe^{III}-Fe^{III} antiferromagnetic exchange. For the calculations involving Fe^{III}-Fe^{III} exchange, first order perturbation theory corrections to the wavefunction were considered taking corrections from 1-Fe exchange.

Section 1.5—Comparison with experiments: Figure 4.

Section 1.5—Comparison with experiments: Figure 5.

I would suggest making the x axis in Fig. 2a,c more clear.

We thank the referee for this suggestion. We have updated the x-axis for Fig. 2c (that is shared with Fig. 2a) to explicitly include the Ising state indices. We have mentioned explicitly in the caption how each Ising spin configuration of Dy^{III} $\boldsymbol{\sigma}$ is mapped to the state index represented on the x-axis.

Section 1.4—Low energy spectrum: Figure 2.

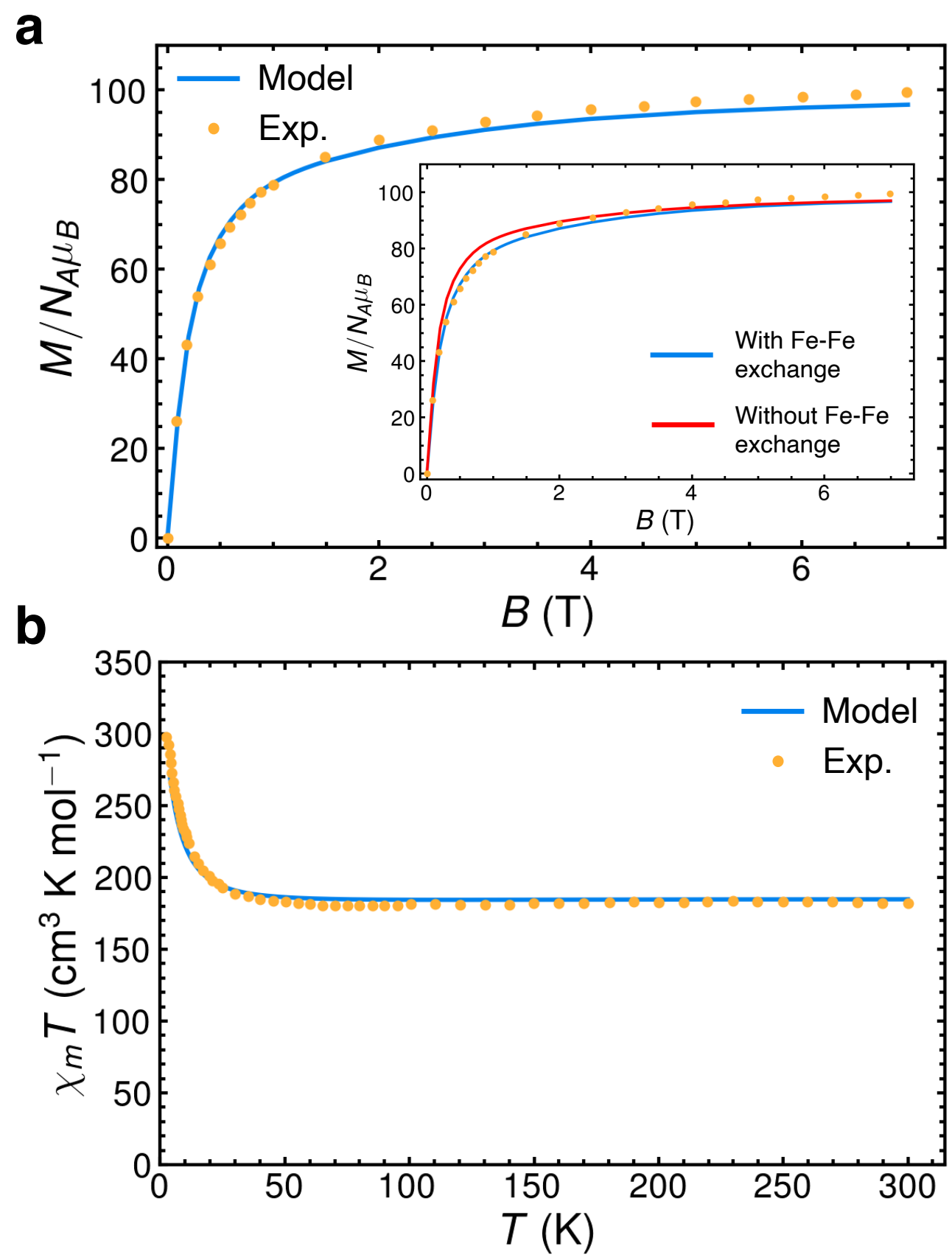

FIG. 3: Magnetic measurements and simulated properties of $Fe_{10}Dy_{10}$. (a) Comparison between experimental measurements (orange circles) and simulated (solid blue line) isothermal powder magnetisation at $T = 2$ K. Inset: comparison between the experimental and simulated $T = 2$ K powder magnetisation when Fe^{III}-Fe^{III} next-nearest neighbour exchange J_C is switched on/off (blue/red curves) in our model. (b) Comparison between experimental (orange circles) and simulated (solid blue line) molar magnetic susceptibility $\chi_m T$ as a function of temperature.

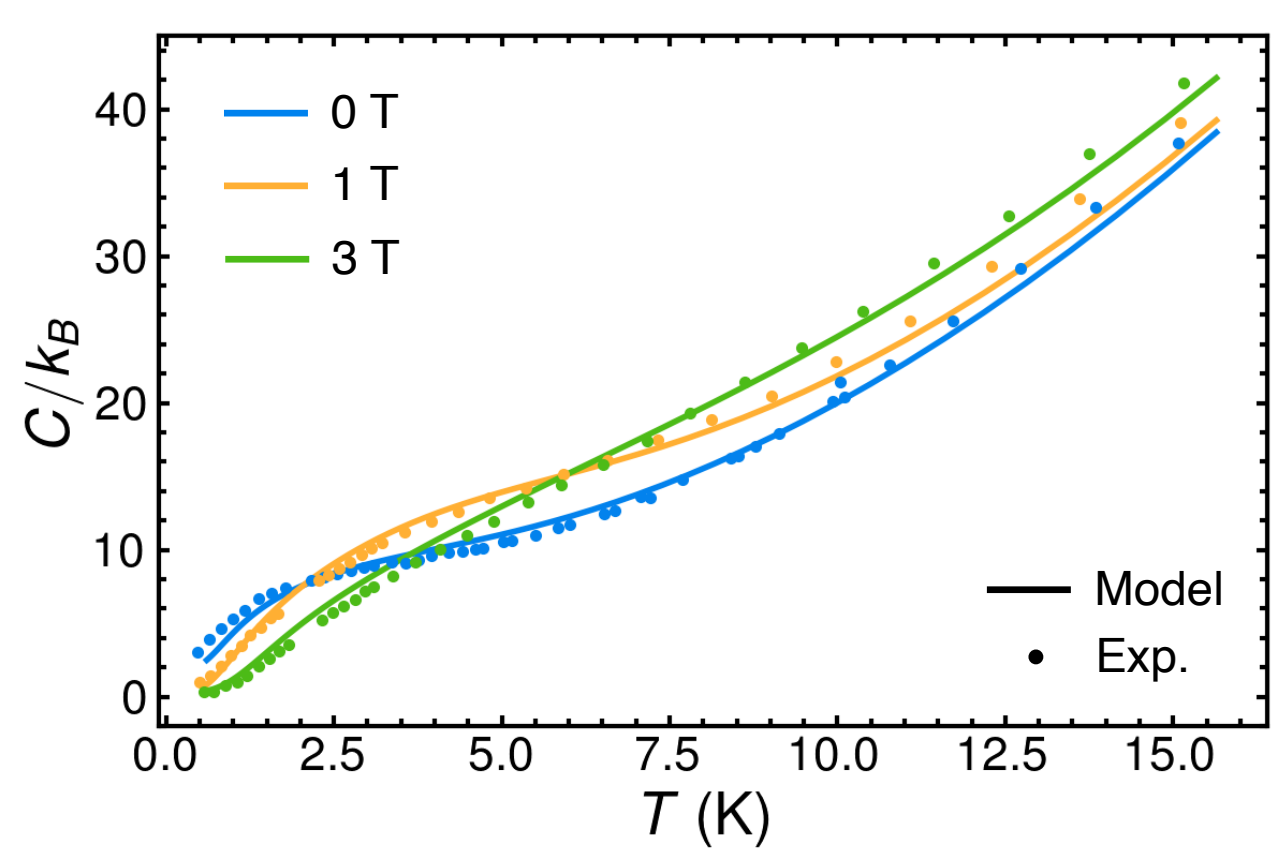

FIG. 4: Specific heat of $Fe_{10}Dy_{10}$. Specific heat measurements (circles) on a $Fe_{10}Dy_{10}$ powder sample as a function of temperature for several values of applied magnetic field $|\mathbf{B}| = 0$ T (blue), 1 T (yellow) and 3 T (green). Simulations (solid lines) using our transfer matrix mode averaged over angular distribution.

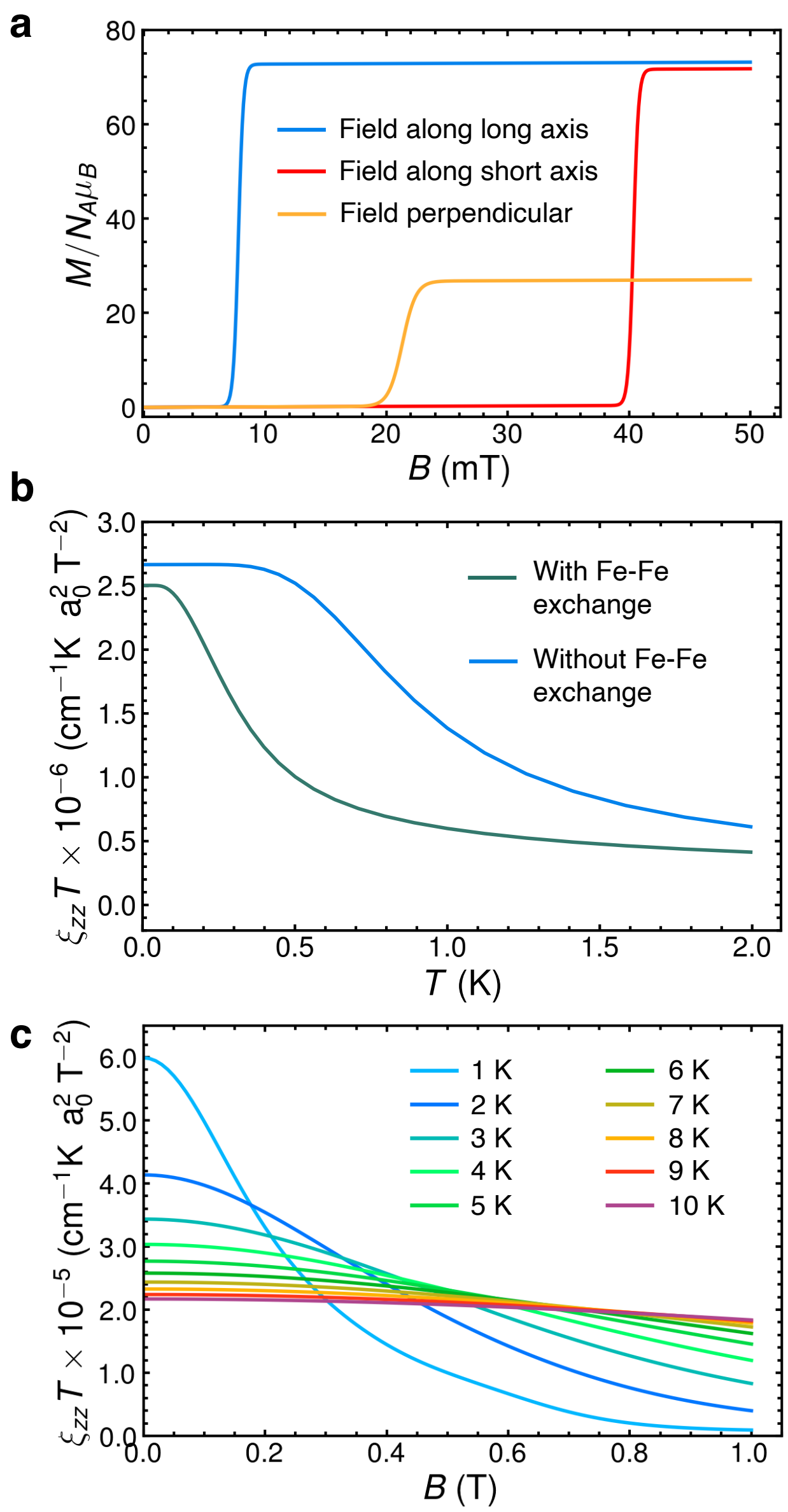

Evidence for a toroidal ground state. (a) $T = 0.01$ K single-crystal magnetisation simulations using our *ab initio*-parameterised transfer matrix model. The field is oriented along the long axis (blue curve), the short axis (red curve) and perpendicular to the $Fe_{10}Dy_{10}$ ring (yellow curve). (b) Low temperature zero-curl toroidal susceptibility simulations with (blue) and without (red) Fe^{III}-Fe^{III} exchange coupling included in the model. (c) Dependence on an applied uniform magnetic field of the toroidal susceptibility, calculated at various temperatures ranging from 1K to 10K.

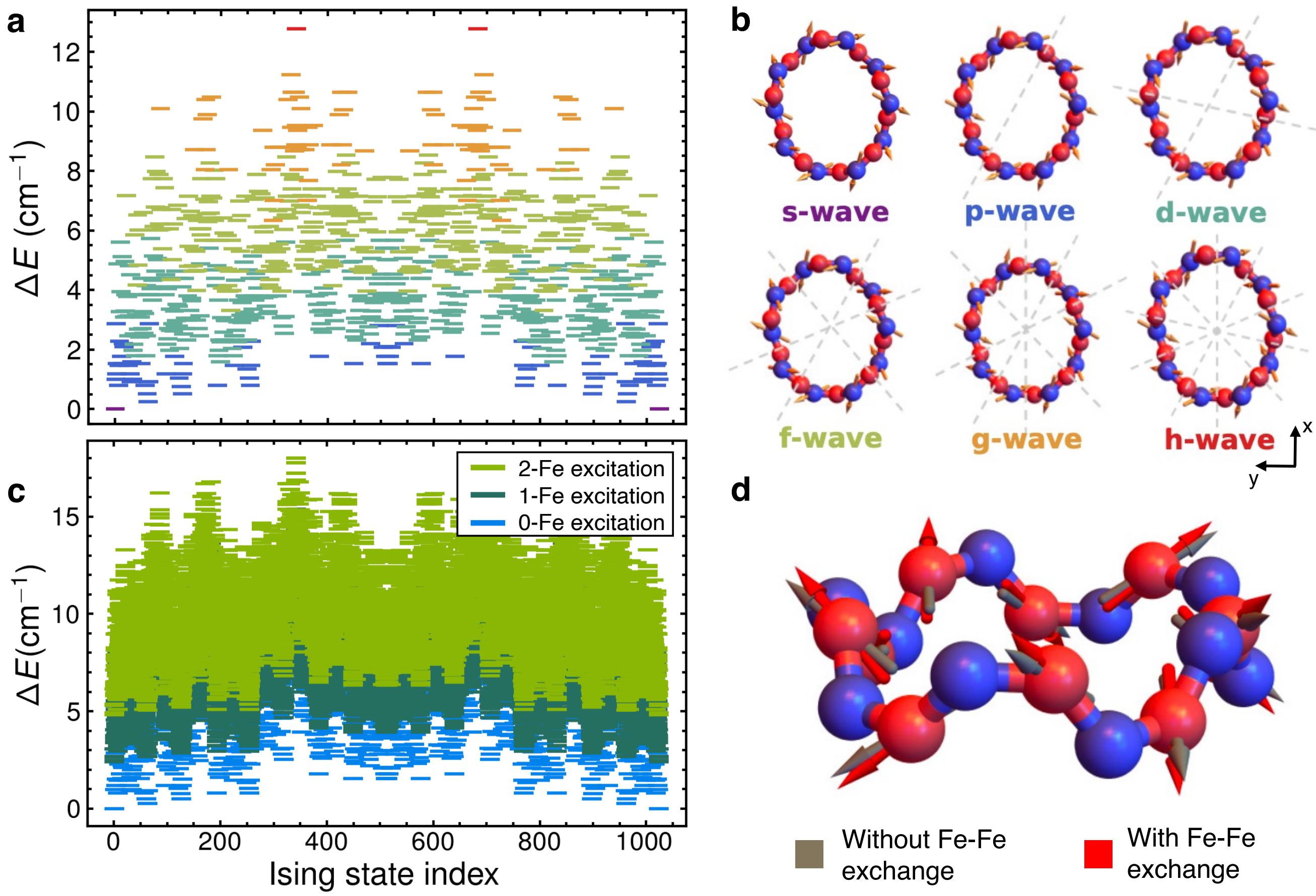

FIG. 2: Low energy spectrum of $Fe_{10}Dy_{10}$.(a) Lowest energy (b) Archetypal examples of s, p, d, f, g and h-wave Ising spin configurations analogous to the spin wave nomenclature. Colour coding corresponds to the band structure shown in (a). (c) Ground and excited state band structure of $Fe_{10}Dy_{10}$ from all possible one and two Fe^{III} excitations around the ring for fixed Dy^{III} Ising spin configurations. The Ising spin configurations are indexed by mapping the vector $\boldsymbol{\sigma}$ to binary representation and straightforwardly converting to a decimal representation offset by 1. (d) Calculated ground state Fe^{III} on-site spin expectation values for the toroidal Dy^{III} Ising spin configuration calculated with (red arrows) and without (grey arrows) Fe^{III}-Fe^{III} antiferromagnetic exchange. For the calculations involving Fe^{III}-Fe^{III} exchange, first order perturbation theory corrections to the wavefunction were considered taking corrections from 1-Fe exchange.

REVIEWER 2

This manuscript presents a compelling and largely well-substantiated framework for finite-temperature toroidal physics in a large $3d$–$4f$ molecular ring, and introduces a useful linear-response quantity, the toroidal susceptibility ξ, to rationalise how a formally non-magnetic toroidal ground state can nonetheless yield an appreciable macroscopic toroidal polarisation at experimentally accessible temperatures. The combination of ab initio parameterisation with an efficient transfer-matrix treatment is technically strong, and the manuscript is generally clear and well organised.

In my view, the core claims are supported at the current level, and the work is suitable for publication after minor revisions that mainly tighten phrasing, improve "measurement-readiness" of the proposal, and clarify a few modelling and electromagnetic assumptions.

No fundamental re-analysis or new experiments are required, but the authors should address the points below to align the strength of the wording with what is quantitatively demonstrated and to improve accessibility for a broad Nature Communications readership.

We thank the referee for their time, careful reading of the manuscript, and positive assessment of our work. We address the points raised below in full.

Essential minor revisions:

1) Calibrate the "direct observation" wording and add a concise readout statement.

The manuscript motivates an experimentally realistic route to lifting the degeneracy of opposite toroidal chiralities via $\nabla \times \mathbf{B}$ under ultrafast driving. This is convincing as a feasibility argument. To avoid overstating the outcome, I suggest the authors slightly soften or qualify "direct observation" in the title/abstract/main text unless a specific readout is clearly articulated.

A minimal, minor-revision-compliant fix is to add a short paragraph stating:

- what physical observable is expected to change upon inducing toroidal polarisation (even if only proposed, not yet implemented), and
- what qualitative signature would distinguish toroidal polarisation from conventional magnetic or thermal effects.

This can be done without adding new data, for example by naming a primary candidate observable (optical probe, symmetry-based nonlinear response, or magnetoelectric-type signal) and explaining, at a high level, what signature is expected.

We thank the referee for this constructive suggestion. We agree that, in the original submission, the discussion of experimental observability and readout mechanisms could have been articulated more explicitly, particularly in relation to the wording surrounding "direct observation" of toroidal polarisation.
In the revised manuscript we have therefore substantially expanded the discussion of experimentally accessible toroidal readout protocols. In particular, we now introduce an explicit nonequilibrium modelling framework describing ultrafast-driven accumulation of finite-temperature toroidal polarisation under temporally asymmetric electric-field pulses, together with its magnetoelectric readout via the induced in-plane magnetic moment generated by an external static electric field. The revised discussion also clarifies the qualitative distinction between this toroidal response and conventional magnetic-field-induced magnetisation effects.
Accordingly, the revised manuscript now goes beyond a purely feasibility-based argument and identifies experimentally realistic dynamical regimes for both the generation and detection of toroidal polarisation.

Changes:

- Added the entirely new Section 1.7, "Ultrafast-driven generation and magnetoelectric readout of toroidal polarisation", explicitly introducing experimentally realistic protocols for the generation, accumulation and readout of finite-temperature toroidal polarisation.

- Completely revised the "Discussion and Conclusions" section in order to better calibrate the scope of the claims concerning experimentally accessible toroidal polarisation and its direct detection.
- Added the entirely new Methods Sections 3.6 and 3.7 describing the nonequilibrium Lindblad dynamics modelling the ultrafast-driven toroidal accumulation, and the *ab initio*–informed methodology developed for the calculation of the magnetoelectric tensor in $Fe_{10}Dy_{10}$.
- Added the entirely new Supplementary Notes 6 and 7 providing additional theoretical and computational details supporting the proposed toroidal generation and readout protocols.

2) Make ξ operational, clarify units, and provide one "worked example" mapping $\nabla \times \mathbf{B}$ to $\langle \boldsymbol{\tau} \rangle$.

ξ is a strong conceptual contribution, but readers will benefit from one explicit bridge from definition to predicted magnitude. Please add:

- a brief dimensional analysis or unit convention for ξ, and
- one representative calculation, using your own estimated $\nabla \times \mathbf{B}$, to report the predicted induced $\langle \boldsymbol{\tau} \rangle$ (or polarisation fraction relative to saturation) at a few temperatures.

This can be presented as a short inset in the main text or a short Supplementary Note. It should not require additional computations beyond what is already in hand, since it is essentially a re-expression of existing results.

We thank the Reviewer for this helpful suggestion. We agree that the operational meaning of $\xi_{\alpha\beta}$ benefits from being made explicit, particularly for readers less familiar with toroidal response functions.
In the revised manuscript we now clarify the units of $\xi_{\alpha\beta}$ immediately after its definition. Since the free energy is expressed in cm^{-1} and the magnetic-field curl in T a_0^{-1}, $\xi_{\alpha\beta}$ is reported in units of $cm^{-1} a_0^2$ T^{-2}. We also added the explicit linear-response relation connecting the induced toroidal polarisation to an applied magnetic-field curl.
Finally, we now provide representative numerical estimates using the peak magnetic-field curl scale motivated by the ultrafast near-IR driving protocol introduced later in Section 1.7. Using the values of $\xi_{zz}T$ reported in Fig. 5b, we estimate the fully saturated ground-state toroidal moment to be $\sim 1.3 \times 10^3\, a_0\mu_B$, while the induced finite-temperature toroidal polarisation is $\sim 6.1 \times 10^2$, $\sim 5.3 \times 10^1$, and $\sim 1.5 \times 10^1\, a_0\mu_B$ at $T = 0.1$, 0.5, and 1 K, respectively. These estimates provide the requested bridge between the definition of $\xi_{\alpha\beta}$ and the expected magnitude of the induced toroidal moment, while also illustrating the thermal degradation of the toroidal response.

Changes:

- Added the following unit convention immediately after the definition of $\xi_{\alpha\beta}$ in Section 1.6:

  "Considering the free energy expressed in cm^{-1} and the magnetic-field curl in T a_0^{-1} (a_0 the Bohr radius), $\xi_{\alpha\beta}$ is reported in units of $cm^{-1} a_0^2$ T^{-2}."

- Added the explicit constitutive relation (new Eq. (7)) between induced toroidal polarisation and magnetic-field curl:

  "In zero magnetic-field curl the equilibrium ensemble remains globally unpolarised, while the induced linear toroidal polarisation generated by an external curl perturbation is given by

  $$\tau_\alpha(T) = \sum_\beta \xi_{\alpha\beta}(T)\, (\nabla \times \mathbf{B})_\beta\, .$$

  "

- Added an operational worked example in Section 1.6 using a realistic magnetic-field curl scale motivated by the ultrafast-driving protocol introduced later in Section 1.7. The new paragraph estimates the fully saturated ground-state toroidal moment from the $T \to 0$ limit of ξT, and reports representative induced finite-temperature toroidal moments at $T = 0.1$, 0.5, and 1 K.

  "Using the peak magnetic-field curl estimated for the ultrafast near-IR driving protocol discussed in Section 1.7, $(\nabla \times \mathbf{B})_z \sim 5 \times 10^{-4}$ T nm^{-1} $\sim 2.65 \times 10^{-5}$ T a_0^{-1}, Eq. (7) predicts induced toroidal moments $\tau_\alpha(T) \sim 6.1 \times 10^2$, $\sim 5.3 \times 10^1$, and $\sim 1.5 \times 10^1$ $a_0\mu_B$ at $T = 0.1$, 0.5, and 1 K, respectively, showing that even at 0.1 K the induced finite-temperature toroidal polarisation remains about 45% of the fully saturated ground-state toroidal moment, before progressively degrading at higher temperatures."

3) Refine "parameter-free" statements for precision.

Because the lattice heat-capacity background includes fitted coefficients, I recommend revising "parameter-free" phrasing to something more precise, such as: "magnetic Hamiltonian parameters obtained from ab initio calculations without fitting to thermodynamic data; lattice background captured with a minimal empirical correction." This is a wording correction only, but it matters for reviewer and editor perception.

We agree with the Reviewer that the previous wording "parameter-free" could be interpreted too broadly given the presence of a fitted lattice heat-capacity background. We have therefore revised the relevant passages in the manuscript in order to distinguish more clearly between the fully *ab initio*-parameterised magnetic Hamiltonian and the minimal empirical treatment of the lattice contribution.

Changes:

- In Section 1.5 "Comparison with experiments" and in the caption of Fig. 5, the wording "parameter-free" has been replaced by formulations explicitly referring to the *ab initio*-parameterised magnetic Hamiltonian and transfer-matrix simulations.

4) Add a brief robustness statement on the ab initio exchange parameterisation.

The broken-symmetry DFT strategy with lanthanide substitution and scaling is reasonable and common for $4f$ systems. To pre-empt concerns, please add either:

- a short sensitivity statement (for example, qualitative invariance of the main conclusions under modest percent-level perturbations of the leading exchanges), or
- a concise justification of why the extracted ξ and the key low-energy features are not fine-tuned to a narrow parameter set.

This can be brief and qualitative, but it should be explicitly stated.

We thank the Reviewer for this important suggestion. In the revised manuscript we now address the robustness of the exchange parameterisation explicitly through an independent statistical χ^2 analysis of the magnetic susceptibility data using a simplified three-parameter exchange model. The resulting confidence intervals confirm that the broken-symmetry DFT exchange parameters lie well within the statistically acceptable region of parameter space.
This analysis demonstrates that the key low-energy toroidal features and finite-temperature response properties discussed in the manuscript are not fine-tuned to a narrow set of exchange parameters, but remain stable across a physically reasonable range of couplings consistent with experiment.

Changes:

- Added a new robustness analysis of the exchange parameterisation in Supplementary Note 8 based on an independent three-parameter χ^2 fit of the magnetic susceptibility data.
- Added the following statement at the end of Section 1.2 "DFT magnetic coupling constants":

> "To further assess the robustness of these exchange parameters, we performed an independent statistical χ^2 analysis based on a three-parameter fit of the magnetic susceptibility data (Supplementary Note 8). The best-fit values, $J_A^{\rm fit} = 0.8^{+0.2}_{-0.15}$ cm^{-1}, $J_B^{\rm fit} = 0.6^{+0.7}_{-0.55}$ cm^{-1} and $J_C^{\rm fit} = -0.10^{+0.10}_{-0.05}$ cm^{-1}, are consistent with the broken-symmetry DFT estimates, which lie within the 68% confidence region of the fit. This independent analysis validates both the magnitude and sign of the dominant exchange interactions used in the *ab initio*-parameterised transfer-matrix model."

5) Clarify the electromagnetic driving assumptions (carrier vs envelope) and experimental boundary conditions.

The ultrafast-pulse estimate is an important part of the "realistic conditions" message. A minor revision should clarify:

- whether $\frac{\partial \mathcal{E}}{\partial t}$ is treated at the carrier level or via the pulse envelope, and why cycle-to-cycle cancellation does not wash out the effective driving of toroidal chirality, and

- what sample form is assumed (single crystal, oriented ensemble, pellet), including a short note on orientation averaging and how it impacts the expected macroscopic polarisation signal.

If a fully oriented sample is required, please state that explicitly and add one or two sentences suggesting a practical alignment route. This can remain at a proposal level.

We thank the Reviewer for these important clarification requests. Relative to the original submission, the revised manuscript now contains a substantially expanded discussion of the experimentally realistic driving and readout protocol (new Section 1.7 together with Methods Sections 3.6–3.7 and Supplementary Notes 6–7). In particular, we now present both a simple two-level model illustrating the toroidal accumulation mechanism under asymmetric ultrafast driving and realistic Lindblad master-equation simulations of toroidal polarisation build-up in $Fe_{10}Dy_{10}$ under near-infrared excitation.
In the present treatment the time-varying electric field $\mathcal{E}(t)$, and thus its time derivative $\frac{\partial \mathcal{E}}{\partial t}$ entering the effective magnetic-field curl, are treated explicitly at the carrier-wave level rather than through a slowly varying pulse-envelope approximation. The key point is that the proposed waveform is temporally asymmetric within each optical cycle, so that opposite toroidal chiralities experience inequivalent nonequilibrium pumping pathways despite the condition $\int_{\text{cycle}} \mathcal{E}(t)\,dt = 0$. As a consequence, the induced toroidal population imbalance does not cancel cycle-by-cycle, but accumulates progressively under the combined action of coherent driving and relaxation. We suggested a sawtooth-like waveform because it can be approximated efficiently using only the first few Fourier harmonics of near-infrared radiation (see e.g. Fig. 1b of González de Alaiza Martínez *et al.*, *Phys. Rev. Lett.* **114**, 183901 (2015), and also our new Fig. 6a/b).
The revised proposal is formulated for an oriented molecular sample/single crystal, with both the ultrafast driving field and the magnetoelectric readout geometry defined relative to the molecular inertia frame in order to maximise the induced toroidal polarisation signal. While we have not attempted to model orientational disorder or powder averaging in the present work, the revised manuscript now makes the assumed experimental geometry and field orientations fully explicit.

Changes:

- Added the entirely new Section 1.7 "Ultrafast-driven generation and magnetoelectric readout of toroidal polarisation", substantially expanding the discussion of experimentally realistic toroidal accumulation and readout protocols.
- Added new Methods Sections 3.6 and 3.7 detailing the ultrafast-driving protocol, Lindblad master-equation simulations, and magnetoelectric response calculations.
- Added new Supplementary Notes 6 and 7 presenting:
  - a two-level toy-model analysis of toroidal accumulation under temporally asymmetric driving, and
  - realistic nonequilibrium simulations of toroidal polarisation build-up in $Fe_{10}Dy_{10}$ under near-infrared sawtooth excitation, including thermal and relaxation effects.
- Revised the Discussion and Conclusions sections in order to integrate the new experimentally realistic nonequilibrium driving and magnetoelectric readout framework into the overall physical interpretation of the manuscript.
- Added a new discussion in Section 1.7 clarifying that the ultrafast driving mechanism is treated explicitly at the carrier-wave level, and that the proposed asymmetric waveform generates a nonequilibrium toroidal population imbalance that does not cancel cycle-by-cycle despite the condition $\int_{\text{cycle}} \mathcal{E}(t)\,dt = 0$.
- Clarified throughout Section 1.7 the experimental geometry assumed for the quantitative estimates, namely an oriented single-crystal setting in which the ultrafast driving field and magnetoelectric readout configuration are defined relative to the molecular inertia frame so as to maximise the induced toroidal polarisation signal.

6) Promote one key supporting result from the Supplementary Information into the main text.

Some of the most convincing material that reconciles the toroidal ground state picture with dynamical magnetisation behaviour appears to be in the Supplementary Information. I suggest moving a representative panel (or adding a short "SI summary" paragraph) into the main text to reinforce the physical interpretation and make the narrative more self-contained.

We thank the Reviewer for this suggestion. We agree that the low-temperature magnetisation dynamics provide useful supporting information regarding the complex low-energy structure of the system. However, we prefer to keep the corresponding analysis in the Supplementary Information rather than promoting it into the main text.

The reason is that conventional magnetic relaxation dynamics driven by a uniform magnetic field do not directly probe the toroidal degree of freedom itself, and their interpretation in large SMT systems such as this one may involve several competing low-lying magnetic manifolds and kinetic relaxation pathways beyond the toroidal sector. We therefore prefer to avoid over-interpreting magnetic hysteresis dynamics as direct evidence of toroidal polarisation.
Instead, in the revised manuscript we substantially expanded the discussion of nonequilibrium toroidal dynamics driven directly by the conjugate field $\nabla \times \mathbf{B}$ (new Section 1.7 together with Methods Sections 3.6–3.7 and Supplementary Notes 6–7). In particular, we now present explicit ultrafast-driving simulations and magnetoelectric readout protocols designed to couple preferentially to the toroidal degree of freedom itself rather than indirectly through conventional magnetic-field-driven relaxation processes. We therefore believe it is conceptually cleaner to retain the magnetisation-dynamics discussion as supporting material, while focusing the main text on experimentally validated static observables used to benchmark the microscopic quantum-state model, together with proof-of-principle simulations of ultrafast toroidal dynamics driven directly by the conjugate field $\nabla \times \mathbf{B}$. The goal of the latter is not to optimise phenomenological relaxation parameters against a specific nonequilibrium experiment, but rather to identify and propose experimentally realistic protocols capable of directly preparing and reading out toroidal polarisation within a physically plausible range of dynamical parameters.

Changes:

- Added a substantially expanded nonequilibrium toroidal-dynamics framework (Section 1.7, Methods 3.6–3.7, Supplementary Notes 6–7) explicitly aimed at identifying experimentally realistic protocols for the direct preparation and magnetoelectric readout of toroidal polarisation within physically plausible dissipative regimes.

- Added the following clarification at the end of Section 1.7:

> "Finally, the precise pulse counts and optimal waiting times should be regarded as relaxation-regime dependent, as they are controlled by plausible but phenomenological relaxation rates within a complex dissipative framework. Rather than attempting exhaustive optimisation of the dynamical model, our simulations show that experimentally detectable toroidal accumulation emerges within physically plausible dissipative regimes."

Minor edits and presentation suggestions (non-essential but recommended).

1. Standardise unit conversions and explicitly relate quoted splittings to kBT at the temperatures emphasised in the discussion.

We thank the Reviewer for this suggestion. Rather than systematically rewriting all energy scales in units of k_BT across the manuscript, which spans several distinct temperature regimes within static and dynamical setups, we instead introduced explicit temperature-energy comparisons and finite-temperature estimates at the key locations where thermal accessibility of low-energy excitations plays an important physical role.

Changes:

- In Section 1.6 we added explicit finite-temperature estimates connecting the calculated toroidal susceptibility $\xi_{\alpha\beta}$, the ultrafast-driving magnetic-field curl amplitudes, and the resulting induced toroidal polarisation at representative temperatures ($T = 0.1$, 0.5, and 1 K), thereby making the relevant thermal energy scales operational within the discussion.

> "In the limit $T \to 0$, $\xi_{\alpha\alpha}T = |\langle 0|\, \tau_\alpha \,|0\rangle|^2/k_B$, hence the low-temperature value $\xi_{zz}T \sim 2.5 \times 10^6$ cm^{-1} K a_0^2 T^{-2} yields a ground-state toroidal moment $|\langle 0|\, \tau_\alpha \,|0\rangle| \sim 1.3 \times 10^3\ a_0\mu_B$.
> Using the peak magnetic-field curl estimated for the ultrafast near-IR driving protocol discussed in Section 1.7, $(\nabla \times \mathbf{B})_z \sim 5 \times 10^{-4}$ T nm^{-1} $\sim 2.65 \times 10^{-5}$ T a_0^{-1}, Eq. (7) predicts induced toroidal moments $\tau_\alpha(T) \sim 6.1 \times 10^2$, $\sim 5.3 \times 10^1$, and $\sim 1.5 \times 10^1\ a_0\mu_B$ at $T = 0.1$, 0.5, and 1 K, respectively, showing that even at 0.1 K the induced finite-temperature toroidal polarisation remains about 45% of the fully saturated ground-state toroidal moment, before progressively degrading at higher temperatures."

2. Add a compact parameter table (Hamiltonian terms, numerical settings) and ensure it is easy to locate.

We thank the Reviewer for this suggestion. The heavily revised version of the manuscript already reports the principal Hamiltonian parameters, exchange couplings, driving amplitudes, and dissipative rates locally within the specific sections where they are physically introduced and discussed (including the detailed exchange-coupling table in Section 1.2 and the numerical settings introduced in Section 1.7 together with the associated Methods and Supplementary sections).

Given the broad range of physical models now treated in the manuscript — spanning equilibrium thermodynamics, transfer-matrix calculations, ultrafast driving, magnetoelectric response, and open-system dynamics — we felt that collecting all numerical quantities into a single global parameter table would reduce rather than improve clarity. We nevertheless carefully revised the manuscript and Supplementary Information to ensure that all relevant parameters and numerical settings are clearly defined and easy to locate within their respective physical context.

3. Consider a short "Data and code availability" note indicating where scripts or inputs for the transfer-matrix calculations can be accessed, if feasible.

We thank the Reviewer for this suggestion. The transfer-matrix, nonequilibrium dynamical, and magnetoelectric simulations presented in this work were performed using in-house research code developed specifically for the present project. While the implementation is substantial, the underlying methodology and numerical framework are described in detail throughout the main text, Methods sections, and Supplementary Notes.
The code is currently undergoing active development and extension for ongoing research projects building upon the present work. For this reason, we prefer not to maintain a public release version at this stage. Nevertheless, we are happy to provide additional implementation details and reasonable access to scripts or input files upon request for the purposes of clarification and reproducibility.

Changes:

- Added a "Data and Code Availability" statement clarifying the status of the in-house transfer-matrix, nonequilibrium dynamical, and magnetoelectric simulation code used in the present work, together with the availability of additional implementation details upon reasonable request.

4. Proofread the Supplementary Information for typographical issues and consistent notation.

We thank the Reviewer for this suggestion. We carefully proofread the revised Supplementary Information in order to improve typographical consistency, notation uniformity, and overall readability throughout the document.

The manuscript is conceptually strong, technically sophisticated, and well aligned with Nature Communications scope. Addressing the minor points above will improve precision, reproducibility, and the credibility of the proposed "direct observation" pathway without changing the substance of the results.

REVIEWER 3

This manuscript presents a comprehensive theoretical investigation of toroidal moments in an icosanuclear $Fe_{10}Dy_{10}$ molecular ring, introducing the novel concept of toroidal susceptibility ξ and demonstrating the possibility of finite-temperature toroidal polarization. The work combines ab initio calculations (CAHF/CASCI-SO for lanthanide crystal fields, broken-symmetry DFT for exchange couplings) with an elegant transfer matrix approach to handle the 62-billion-dimensional Hilbert space. The authors achieve good quantitative agreement with experimental magnetic and calorimetric data and propose an experimental pathway for direct observation of molecular toroidal moments using femtosecond laser pulses.

The manuscript makes significant contributions to the field of molecular magnetism and toroidal electrodynamics. The introduction of toroidal susceptibility as a thermodynamic response function is very useful, providing a quantitative framework for understanding finite-temperature toroidal properties. However, the manuscript has several weaknesses that should be addressed before publication.

We thank the Reviewer for their careful reading of the manuscript and for recognising both the conceptual significance of the toroidal susceptibility framework and the technical advances underlying the present work. In particular, we appreciate the Reviewer's recognition of the transfer-matrix methodology and of the finite-temperature toroidal-response framework introduced in the manuscript. We have carefully addressed the points raised below and substantially revised and expanded the manuscript accordingly.

1. Missing Magnetoelectric Coupling Analysis: A critical weakness is the absence of magnetoelectric (ME) coupling investigation despite the manuscript's emphasis on toroidal moments. The fundamental importance of toroidal moments lies in enabling linear ME coupling—electric control of magnetism—as correctly noted in the introduction [Spaldin et al., J. Phys.: Condens. Matter 20, 434203 (2008)]. However, the manuscript provides no analysis of ME coupling in $Fe_{10}Dy_{10}$: no calculation of the ME tensor α, no investigation of electric field effects on toroidal states, and no discussion of electric-field control as an alternative to the proposed magnetic field curl approach.

This omission is particularly significant given that: (1) recent studies have demonstrated ME coupling in related molecular systems [Yazback et al., J. Phys. Chem. A 127, 3814 (2023); Lewkowitz et al., Sci. Rep. 13, 2769 (2023)]; (2) electric field control would be more experimentally accessible than the proposed ultrafast laser configuration; and (3) ME coupling represents the primary technological advantage of toroidal systems. The manuscript would be substantially strengthened by adding computational investigation of ME effects and discussing electric-field manipulation of toroidal states.

We thank the Reviewer for highlighting the central importance of magnetoelectric (ME) coupling in toroidal molecular systems. We fully agree that the possibility of coupling toroidal degrees of freedom to experimentally accessible electric fields represents one of the main physical motivations underlying toroidal electrodynamics.

In response to this criticism, the revised manuscript has been substantially expanded through the addition of the entirely new Section 1.7, where we now propose an experimentally realistic framework for the dynamical preparation and magnetoelectric readout of toroidal polarisation in $Fe_{10}Dy_{10}$. In particular, we now present explicit calculations of the molecular ME tensor together with a proposed ME readout mechanism for the toroidal polarisation generated via a newly introduced temporally asymmetric ultrafast-driving protocol. The revised manuscript now explicitly evaluates the magnetic moment induced by an external electric field in a toroidally polarised $Fe_{10}Dy_{10}$ system and discusses the role of electric-field-induced magnetic response in relation to the toroidal states.

The optical toroidal accumulation protocol and the associated magnetoelectric readout strategy are now described in detail in the new Section 1.7, while the general implementation framework for the ME tensor calculations is presented in the new Method Section 3.7 and Supplementary Note 6. The revised Discussion and Conclusions section also now compares the estimated ME splittings with previous literature values for molecular toroidal systems.

We also note that the present work focuses specifically on strongly spin–orbit-coupled and non-frustrated SMTs, where toroidal states emerge from noncollinear anisotropic magnetic textures and can exhibit finite-temperature blocking phenomena qualitatively distinct from the weak spin–orbit frustrated-spin regime typically discussed for transition-metal trimers. In the revised Introduction we now explicitly discuss recent studies on spin triangles and magnetoelectric effects [22,23], while clarifying the different physical regimes addressed by these approaches.

Finally, while the revised manuscript now includes explicit magnetoelectric calculations and readout protocols, the primary focus of the nonequilibrium analysis remains the identification of experimentally realistic dynamical preparation and detection schemes for toroidal polarisation in large SMTs. We therefore chose to focus the ultrafast-driving framework on temporally asymmetric near-infrared pulses dynamically generating effective magnetic-field curls directly coupled to the

toroidal degree of freedom, while using the magnetoelectric response primarily as an experimentally accessible readout channel. A broader equilibrium investigation of magnetoelectric response across different SMT classes, including a more systematic comparative analysis of ME mechanisms and scaling behaviour, lies beyond the scope of the present work and is now briefly discussed in the revised Discussion and Conclusions section.

Changes:

- Added the entirely new Section 1.7 "Ultrafast-driven generation and magnetoelectric readout of toroidal polarisation", proposing an experimentally realistic framework for the preparation and detection of toroidal polarisation in strongly spin–orbit-coupled SMTs.
- In Section 1.7 we now explicitly discuss the magnetoelectric effect as a readout mechanism for induced toroidal polarisation, including explicit calculations of the magnetic moment induced by an external electric field in a toroidally polarised $Fe_{10}Dy_{10}$ system.
- Added new Method Section 3.7 and Supplementary Note 6 detailing the implementation and evaluation of the magnetoelectric response calculations in large SMT systems.
- Expanded the Discussion and Conclusions sections to include comparison of the estimated magnetoelectric splittings with previous literature values for molecular toroidal systems.
- Revised the Introduction to include additional discussion of recent literature on molecular toroidal moments and magnetoelectric coupling, including Yazback *et al.* and Lewkowitz *et al.*, while clarifying the distinction between weak spin–orbit frustrated-spin triangles and strongly spin–orbit-coupled non-frustrated SMTs:

> Recent theoretical and experimental studies on transition-metal spin triangles have explored electric-field control of chiral, magnetic and toroidal-like spin states [22,23]. In such weak spin–orbit spin-frustrated systems, toroidal character is closely intertwined with spin chirality and electric-field-dependent exchange interactions, whereas the direct preparation and readout of a finite-temperature toroidal polarisation in strongly spin–orbit-coupled and non-frustrated SMTs remains an open challenge.

2. Literature Coverage and Contextualization: The manuscript claims (page 3) that "toroidal properties have only been identified and discussed for specific molecular microstates, while the collective role of toroidal excitations within a molecular system and their contribution to a finite-temperature toroidal polarization have largely been ignored." This statement overlooks recent systematic theoretical investigations of toroidal moments in molecular systems. Specifically: Yazback et al. [J. Phys. Chem. A 127, 3814-3823 (2023)] performed comprehensive first-principles studies of toroidal moments in transition-metal trimers (Cr_3 and Cu_3), demonstrating:

1. Finite ground-state toroidal moments in Cu_3 ($M_t = 0.895$ Åμ_B, 32% of maximum);
2. Toroidal moments in both ground and excited states;
3. Systematic parameter-space exploration identifying $> 194,000$ spin models with high toroidal moments ($> 99\%$ of maximum);
4. Design principles showing most high-M_t models require specific exchange coupling patterns ($J_{xx} > 0$, $J_{yy} < 0$, $J_{zz} < 0$) and antisymmetric exchange; and
5. Magnetoelectric coupling effects on toroidal moments. This work directly addresses the claimed gaps regarding collective excitations and design principles for molecular toroidal moments and should be cited and discussed.

We thank the Reviewer for pointing out the important recent work by Yazback *et al.* on toroidal moments and electric-field effects in frustrated transition-metal spin triangles. We have now cited and discussed this literature explicitly in the revised Introduction.
After carefully considering these works, we nevertheless believe that the original conceptual distinction made in the manuscript remains valid. While Yazback *et al.* investigate electric-field-dependent changes of effective spin-Hamiltonian parameters and branch-resolved toroidal descriptors for selected low-energy states and frustrated manifolds in weak spin–orbit frustrated triangles, the collective role of thermally populated toroidal excitations and their contribution to a finite-temperature toroidal polarization are not explicitly addressed.
This is complementary to the present work, where the primary focus is the finite-temperature toroidal response of large strongly spin–orbit-coupled and non-frustrated SMT systems together with the dynamical preparation and magnetoelectric readout of finite-temperature toroidal polarisation.

Changes:

- Revised the Introduction to include discussion of recent literature on toroidal states and electric-field effects in transition-metal spin triangles, including Yazback *et al.* and Lewkowitz *et al.*.

> "Recent theoretical and experimental studies on transition-metal spin triangles have explored electric-field control of chiral and toroidal-like spin states [22,23]. In such weak spin–orbit frustrated systems, toroidal character is closely intertwined with spin chirality and electric-field-induced modifications of spin-Hamiltonian parameters, whereas the direct preparation and readout of a finite-temperature toroidal polarisation in strongly spin–orbit-coupled and non-frustrated SMTs remains an open challenge."

- Added Refs. [22,23] to the Introduction and related discussion of toroidal molecular systems and magnetoelectric effects.

3. Experimental Validation of Main Claims: The manuscript title and abstract claim the toroidal moment is "amenable to direct observation." The proposal relies on order-of-magnitude estimates (Eq. 10, page 12) and several unverified assumptions. Specific concerns:

a. Laser-induced heating effects: The estimated fluence (0.03 J/cm^2) and 10 fs pulse duration could cause significant heating. Issues need to be considered include: temperature rise during the pulse, thermal damage threshold for the molecular crystal, effects on the delicate $Fe_{10}Dy_{10}$ structure, etc.

b. Toroidal relaxation time: The authors assume "sufficiently slow toroidal relaxation times" (page 12) but provide no estimate or discussion. What is the expected relaxation time? How does it compare to the pulse duration and measurement timescale?

c. Signal detection: How would the toroidal polarization be measured experimentally? What is the expected signal-to-noise ratio? What detection methods are proposed?

The authors should consider: substantially expand the discussion of experimental challenges, provide more rigorous estimates of heating/damage, relaxation times, and detection schemes, and appropriately temper claims from "amenable to direct observation" to "predicted to be observable under specific conditions pending experimental verification"

We thank the Reviewer for these important comments concerning the experimental viability of the proposed toroidal-polarisation protocol. We agree that the original version of the manuscript only provided an order-of-magnitude estimate for the direct observation proposal. In response, the revised manuscript has been substantially expanded through the addition of the entirely new Section 1.7 together with associated Methods Sections 3.6 and 3.7 and Supplementary Notes 6 and 7.
The revised work now goes significantly beyond the original static estimate by introducing an explicit nonequilibrium framework for the dynamical preparation and magnetoelectric readout of finite-temperature toroidal polarisation in $Fe_{10}Dy_{10}$. In particular, the revised manuscript now directly addresses the three concerns raised by the Reviewer:

- *Laser-induced heating and experimental viability.* We now present explicit estimates of cumulative thermal load and pulse-induced heating under repeated ultrafast excitation, together with discussion of experimentally realistic pulse fluences and repetition protocols compatible with low-temperature operation. In particular, for the two pulse-separation regimes explicitly simulated in the manuscript ($t_{\mathrm{wait}} = 6.3$ ns and 1 ns), starting from a base temperature of 10 mK, the estimated cumulative heating remains limited to approximately 5 mK and 9 mK, respectively, remaining well below both any plausible sample-damage threshold and the characteristic low-energy magnetic excitation scale of $Fe_{10}Dy_{10}$ (of order $\sim 0.26\ \mathrm{cm}^{-1} \approx 370$ mK). The cumulative thermal-load analysis is now explicitly described in Section 1.7, graphically reported in the new Figure 6d, with details of the calculations reported in Methods 3.6 and Supplementary Note 7.

- *Toroidal relaxation times and dissipative dynamics.* The revised manuscript now includes phenomenological tunnelling and spin-phonon relaxation pathways within an open-system master-equation framework describing the nonequilibrium accumulation dynamics of toroidal polarisation under repeated pulse trains. The adopted relaxation parameters are chosen to remain consistent with recent literature estimates for rare-earth molecular magnetic systems while accounting for the expected suppression of collective multi-centre tunnelling processes in a large non-collinear SMT. The dependence of the accumulation efficiency on pulse waiting times explicitly illustrates the competition between toroidal accumulation and relaxation processes within the low-energy manifold.

- *Signal detection and readout protocols.* The revised manuscript now proposes an explicit magnetoelectric readout mechanism for the generated toroidal polarisation. The detectability analysis explicitly includes estimates of the irradiated sample volume, an atomistic calculation of the magnetoelectric response tensor determining the electric-field-induced magnetic signal per molecule, and the nonequilibrium toroidal population accumulation, together with

comparison against literature microSQUID sensitivity thresholds ($\sim 10^4\,\mu_B$). These estimates demonstrate that the predicted nonequilibrium magnetic response lies within experimentally accessible regimes and are now summarised in the new Figure 6c.

The revised manuscript also now explicitly clarifies the intended scope of the nonequilibrium simulations. Rather than attempting an exhaustive optimisation of all dissipative parameters for a specific experimental setup, the purpose of the present framework is to demonstrate that experimentally detectable toroidal accumulation can emerge within physically plausible relaxation regimes in a realistic strongly spin–orbit-coupled SMT.
At the same time, we believe that the revised work now substantially strengthens the original claim that finite-temperature toroidal polarisation in molecular systems can become experimentally accessible through physically motivated preparation and readout protocols directly targeting the toroidal degree of freedom.

Changes:

- Added the entirely new Section 1.7 “Ultrafast-driven generation and magnetoelectric readout of toroidal polarisation”, substantially expanding the discussion of experimentally realistic preparation and detection protocols for toroidal polarisation in $Fe_{10}Dy_{10}$.
- Added new Method Sections 3.6 and 3.7 together with Supplementary Notes 6 and 7, now explicitly discussing:
  - ultrafast-driving conditions and magnetic-field-curl generation,
  - cumulative heating and thermal-load estimates under repeated pulse excitation,
  - phenomenological tunnelling and spin-phonon relaxation dynamics,
  - open-system master-equation simulations of nonequilibrium toroidal accumulation under repeated pulse trains, and
  - magnetoelectric readout mechanisms including atomistic evaluation of the induced magnetic response and comparison with literature microSQUID sensitivity thresholds.
- Added new Figure 6c and Figure 6d explicitly reporting the predicted magnetoelectric detection signal and cumulative pulse-induced heating estimates for the experimentally simulated ultrafast-driving regimes.

Minor issues:

1. Authors shows that as temperature approaches to zero, toroidal moment approaches a maximum value. The zero temperature moment can be evaluate by applying a perturbation that lifts the energy degeneracies. Is there a particular reason to use the method in the manuscript?

We thank the Reviewer for this observation. We agree that in the strict zero-temperature limit the ground-state toroidal moment can be extracted by introducing an infinitesimal symmetry-breaking perturbation lifting the degeneracy of opposite toroidal branches.
The purpose of the present finite-temperature toroidal susceptibility framework, however, is different. Rather than focusing solely on the $T \to 0$ limit, $\xi_{\alpha\beta}$ provides a thermodynamic linear-response description of toroidal polarisation in the presence of thermally populated toroidal excitations and external magnetic-field curls, directly analogous to the role of the Van Vleck magnetic susceptibility in conventional magnetic systems.
We have clarified this point explicitly in the revised manuscript, also providing (i) the value of the zero-temperature toroidal moment associated with the maximally toroidal ground Kramers doublet, and (ii) representative finite-temperature estimates of the induced toroidal moment calculated within the linear-response regime using the toroidal susceptibility tensor $\xi_{\alpha\beta}$.

Changes:

- Added explicit clarification in Section 1.6 that the finite-temperature toroidal susceptibility framework is intended as a thermodynamic linear-response description of toroidal polarisation in the presence of thermally populated toroidal excitations, rather than solely as a zero-temperature symmetry-breaking construction.
- Added explicit zero-temperature and finite-temperature toroidal-moment estimates in Section 1.6:

"In the limit $T \to 0$, $\xi_{\alpha\alpha}T = |\langle 0|\, \tau_\alpha\, |0\rangle|^2/k_B$, hence the low-temperature value $\xi_{zz}T \sim 2.5 \times 10^6$ cm^{-1} K a_0^2 T^{-2} yields a ground-state toroidal moment $|\langle 0|\, \tau_\alpha\, |0\rangle| \sim 1.3 \times 10^3\ a_0\mu_B$.

Using the peak magnetic-field curl estimated for the ultrafast near-IR driving protocol discussed in Section 1.7, $(\nabla \times \mathbf{B})_z \sim 5 \times 10^{-4}$ T nm^{-1} $\sim 2.65 \times 10^{-5}$ T a$_0^{-1}$, Eq. (7) predicts induced toroidal moments $\tau_\alpha(T) \sim 6.1 \times 10^2$, $\sim 5.3 \times 10^1$, and $\sim 1.5 \times 10^1\ a_0\mu_B$ at $T = 0.1$, 0.5, and 1 K, respectively, showing that even at 0.1 K the induced finite-temperature toroidal polarisation remains about 45% of the fully saturated ground-state toroidal moment, before progressively degrading at higher temperatures."

2. Metastability: Is the toroidal ground state truly the global ground state or a metastable configuration?

The toroidal manifold discussed in the manuscript corresponds to the true low-energy ground-state manifold of the effective Hamiltonian rather than a thermodynamically metastable excited configuration. We interpret the Reviewer's question as referring instead to the dynamical persistence and relaxation of nonequilibrium toroidal polarisation. In this respect, the revised manuscript now explicitly includes phenomenological toroidal relaxation pathways within the ultrafast-driven open-system simulations introduced in the new Section 1.7. In particular, the dependence of the accumulated toroidal polarisation on pulse waiting times directly reflects the competition between dynamical toroidal accumulation and relaxation processes within the low-energy manifold. For sufficiently long waiting times, deviations from ideal linear accumulation become visible, signalling the onset of relaxation of the nonequilibrium toroidal state on microsecond timescales within the present dissipative regime. This is clarified in the revised manuscript in Section 1.7, with additional details provided in Methods Section 3.6 and Supplementary Note 7.

---

* Corresponding author: alessandro.soncini@unipd.it
* Corresponding author: annie.powell@kit.edu

Response to referees: Finite-Temperature Toroidal Moment Amenable to Direct Observation in an $Fe_{10}Dy_{10}$ Molecular Ring

Alessandro Soncini,[1,*] Kieran Hymas,[2] Jonas Braun,[3,4,5] Yannik F. Schneider,[3] Simone Calvello,[1] Amer Baniodeh,[3] Yanhua Lan,[3] Wolfgang Wernsdorfer,[5,6] Marco Affronte,[7] Christopher E. Anson,[3] and Annie K. Powell[3,4,5,*]

[1]*Department of Chemical Sciences, University of Padova, Via Marzolo 1, Padova, 35123, Italy*
[2]*Commonwealth Scientific and Industrial Research Organisation, (CSIRO), Clayton, Victoria, 3168, Australia*
[3]*Institute of Inorganic Chemistry, Karlsruhe Institute of Technology (KIT), Kaiserstr. 12, Karlsruhe, 76131, Germany*
[4]*Institute of Nanotechnology, Karlsruhe Institute of Technology (KIT), Kaiserstr. 12, Karlsruhe, 76131, Germany*
[5]*Institute for Quantum Materials and Technologies, Karlsruhe Institute of Technology (KIT), Kaiserstr. 12, Karlsruhe, 76131, Germany*
[6]*Physikalisches Institut, Karlsruhe Institute of Technology (KIT), Kaiserstr. 12, Karlsruhe, 76131, Germany*
[7]*Dipartimento di Scienze Fisiche, Informatiche e Matematiche, University of Modena and Reggio Emilia, Via Campi 213A, Modena, 41125, Italy*

(Dated: June 27, 2026)

We thank the Editor and the three Reviewers for their constructive assessment of our revised manuscript. We are pleased that the manuscript is now considered suitable for publication in *Nature Communications*, subject to minor updates. We have addressed all remaining reviewer comments in the revised manuscript. The reviewers' comments are reproduced below in black, our responses are given in blue, and the corresponding modifications to the manuscript are indicated in red. All new changes are highlighted in red in the revised manuscript.

REVIEWER 1

The manuscript is substantially improved after the revision, and I can recommend it for publication in Nature Communications after several minor updates.

We thank the Reviewer for the positive assessment and have implemented all requested minor changes.

Comment 1

The abstract and the introduction should be formulated in such a way that micro-SQUID will not come as the method used in this work. To my feeling, it is more suitable for the Discussion section.

We agree. We have revised the Abstract and Introduction to avoid implying that micro-SQUID measurements are the method used for the proposed magnetoelectric readout protocol in this work, while retaining the detectability discussion in the readout section.

Changes: The Abstract has been rewritten to describe the preparation-and-readout protocol without mentioning micro-SQUID. In the Introduction, the final readout sentence has been revised to state that the magnetoelectric response is "predicted to yield an electric-field-induced magnetic signal within the sensitivity range of solid-state high-sensitivity magnetometers".

Comment 2

I'm not convinced that the toroidal susceptibility is a new quantity that has nothing in common with the previously discussed quantities (Refs. 52, 53): all of them are naturally introduced as in the Landau theory (see Landau, Theoretical Physics, Vol. 8). But of course, a reader of the manuscript appreciates the calculation specific for the given physical system, which is an important achievement.

We thank the Reviewer for this clarification. We have softened the wording and explicitly connected our molecular linear-response formulation to related finite-temperature toroidal-response quantities.

Changes: In Section 1.6, the paragraph introducing $\xi_{\alpha\beta}$ has been revised. It now acknowledges related quantities in Landau expansions and spin-toroidization calculations, and states that "Building on these related formulations, here we treat $\xi_{\alpha\beta}$ as an explicit finite-temperature molecular linear-response tensor, directly analogous to the Van Vleck magnetic susceptibility, and specifically adapted to discrete, thermally populated toroidal spectra".

Comment 3

The magnetoelectric tensor, which is given in Eq. (14), seems to have too many significant digits that are far beyond the accuracy of the previously used estimates, and, probably, not relevant for any potential experimental access.
We agree and have rounded the components of the magnetoelectric tensor in Eq. (14).
Changes: The numerical entries of the magnetoelectric tensor in Eq. (14) have been rounded, for example from the previously over-specified values to the revised tensor with entries such as 28.2, −2.11, 0.64, etc.

Comment 4

As a minor remark, it would be useful to mention the purely toroidal antisymmetric part of the magnetoelectric tensor in the spirit of discussion by Spaldin et al, JPCM 20 (2008), Eqns. (40-41).
We thank the Reviewer for this suggestion. We have added a concise statement after Eq. (14) identifying the antisymmetric magnetoelectric component with the induced magnetoelectric toroidal moment, consistently with the Methods section.
Changes: A sentence has been added after Eq. (14): "As detailed in the Methods section, the antisymmetric part of this tensor is dual to an induced magnetoelectric toroidal moment [3], here estimated to be nearly aligned with the ground-doublet toroidal moment and of magnitude $|\boldsymbol{\tau}^{\mathrm{ME}}| \simeq 5.13$ a.u.".

REVIEWER 2

The revised manuscript is substantially improved, and my primary concerns from the first review have been addressed in a technically meaningful way. [...] I consider the manuscript close to publishable. I recommend minor revision, limited to a small number of clarifications and one bibliographic correction.
We thank the Reviewer for the positive assessment. We have implemented the remaining minor clarifications and bibliographic correction.

Comment 1

Magnetoelectric coupling analysis (previously missing) Assessment: This item is satisfactorily addressed. The added ME framework is a major improvement and is conceptually well aligned with the technological and fundamental motivation for toroidal states. Minor remaining clarification (optional): The adopted static readout field magnitude is very large; a brief note on practical realisation (for example micro/nano-electrode gap geometry, thin sample, pulsed readout to avoid breakdown) and explicit mention of linear scaling with the readout field would improve experimental transparency.
We have added the requested clarification on the linear scaling with readout field and on the intended high-field readout conditions.
Changes: A clarification has been added after the definition of the static readout field in Section 1.7: "Because the induced magnetic signal scales linearly with the readout field, lower fields require proportionally more pulse–wait sequences to reach threshold. We regard the previously proposed $E_x^{\mathrm{ext}} = 10^9$ V m^{-1} [29,30] as a high-field limit relevant to micro- or nano-electrode, thin-sample, or pulsed-field configurations.".

Comment 2

Literature positioning and scope claims Assessment: The literature positioning is improved and largely acceptable. However, one remaining sentence in the Introduction still reads overly absolute (stating that the collective finite-temperature role of toroidal excitations "has largely been ignored"), which could be interpreted as dismissive of quantitative efforts in adjacent subfields. Minor request: Please soften or qualify this statement explicitly to the specific regime addressed here (strong SOC, non-frustrated SMTs, discrete many-state manifolds, and an operational finite-temperature linear-response/readout framework).
Agreed. We have replaced the previous wording with a more specific statement restricted to the regime and framework addressed here.
Changes: The Introduction has been revised to state: "On the other hand, for strongly spin–orbit-coupled, non-frustrated single-molecule toroics, a quantitative framework connecting the collective contribution of thermally populated toroidal excitations to finite-temperature toroidal linear response and experimental readout has not yet been established.".

Comment 3

Direct observability and feasibility (heating, relaxation, detectability) Assessment: This item is largely addressed and is now presented as a quantitative proof- of-principle protocol. A brief sensitivity note on the heating estimate (for example, scaling of ΔT with absorbed fraction) would further strengthen the "realistic conditions" claim but is not essential if space is limited.
We thank the Reviewer for this suggestion. We have added the requested sensitivity note on the heating estimate.
Changes: A sentence has been added in Section 1.7 after the Beer–Lambert heating estimate: "Heating scales linearly with absorbed fraction, making these estimates proportional to the assumed near-infrared extinction coefficient; even tenfold higher absorption would remain below 0.1 K over the detectability window.".

Comment 4

Additional required correction (bibliography). The revised manuscript already cites the recent Nature Chemistry paper on homochiral toroidal spin states in Dy(III)-based SMTs (currently listed as Ref. 16). Please add the DOI to the reference entry to match standard citation practice and to facilitate direct access to this key, very recent result: DOI: 10.1038/s41557-026-02070-4.
We have updated Ref. 16 with its final bibliographic details and DOI link, as requested.
Changes: Ref. 16 has been updated to include the final bibliographic information and DOI link for the Nature Chemistry article on homochiral toroidal spin states in Dy(III)-based single-molecule toroics.

REVIEWER 3

In response to my original report ("accept with major revisions, requires re-review"), the authors have carried out a substantial body of genuinely new work rather than cosmetic edits. [. . .] A small number of residual caveats remain, but these are largely inherent to a proof-of-principle theoretical proposal and are transparently disclosed by the authors. In my view the manuscript is now suitable for publication.
We thank the Reviewer for the very positive assessment and for considering the manuscript suitable for publication.

Remaining suggestion

A single sentence acknowledging the sensitivity of the heating estimate to the assumed near-IR extinction coefficient would make the analysis more complete. This is the only point I would still raise, and it can be handled at the authors' discretion.
We thank the Reviewer for this helpful suggestion. We have added the requested sentence on the sensitivity of the heating estimate.
Changes: A sentence has been added in Section 1.7 after the Beer–Lambert heating estimate: "Heating scales linearly with absorbed fraction, making these estimates proportional to the assumed near-infrared extinction coefficient; even tenfold higher absorption would remain below 0.1 K over the detectability window.".

* Corresponding author: alessandro.soncini@unipd.it
* Corresponding author: annie.powell@kit.edu
